# PCK2-Mediated PQBP1 Lactylation Promotes Asthmatic Inflammation through PRMT5 Inhibition

**DOI:** 10.34133/research.1321

**Published:** 2026-06-19

**Authors:** Qiaoyun Bai, Ningpo Ding, Rixin Feng, Fengxiang Shang, Zongqi Wang, Liangchang Li, Zhiguang Wang, Yihua Piao, Chongyang Wang, Yilan Song, Guanghai Yan

**Affiliations:** ^1^ Jilin Key Laboratory for Immune and Targeting Research on Common Allergic Diseases, Yanbian University, Yanji 133002, P.R. China.; ^2^ Department of Anatomy, Histology and Embryology, Yanbian University Medical College, Yanji 133002, P.R. China.; ^3^ Department of Respiratory Medicine, Affiliated Hospital of Yanbian University, Yanji 133000, P.R. China.; ^4^ Department of Critical Care Medicine, Affiliated Hospital of Yanbian University, Yanji 133000, P.R. China.; ^5^ Key Laboratory of Natural Medicines of Changbai Mountain, Ministry of Education, Yanbian University, Yanji 133002, P.R. China.

## Abstract

**Background:** Asthma involves chronic inflammation linked to metabolic reprogramming, but how metabolites reshape epigenetics through posttranslational modifications remains unclear. **Methods:** We used house dust mite (HDM)-induced asthmatic mice with multiomics analyses (metabolomics, posttranslational modification-proteomics, and chromatin immunoprecipitation sequencing) and validated findings through gene editing and adeno-associated virus interventions. **Results:** Asthmatic airways showed lactate-driven glutaminolysis, causing lactate/succinate accumulation. Phosphoenolpyruvate carboxykinase 2 (PCK2) succinylation at K100 enhanced stability by antagonizing ubiquitination, creating a lactate-generating feedback loop. Accumulated lactate triggered polyglutamine-binding protein 1 (PQBP1) lactylation at K223, enabling protein arginine methyltransferase 5 (PRMT5)/WD repeat domain 77 complex inhibition. This erased H4R3me2s repressive marks from proinflammatory gene promoters, particularly mitogen-activated protein kinase pathway genes, causing transcriptional derepression. Airway epithelium-specific *Pqbp1* knockout reduced inflammation, goblet cell hyperplasia, and T helper 2 responses. *Pck2*-short hairpin RNA or oxamate treatment ameliorated asthmatic pathology. **Conclusion:** We identified a PCK2–lactate–PQBP1–PRMT5 axis linking metabolic reprogramming to epigenetic dysregulation in asthma. PCK2-K100 succinylation drives lactate accumulation, inducing PQBP1-K223 lactylation that inhibits PRMT5 and activates inflammatory genes, representing a therapeutic target for asthma.

## Introduction

Asthma is a heterogeneous chronic airway disease affecting hundreds of millions of people globally [1,2], with its pathological hallmark being a persistent inflammatory response (www.ginasthma.org; 2024) [3]. While immune cells (e.g., T helper 2 [Th2] cells, and eosinophils) have traditionally been considered the primary effectors, emerging evidence highlights the airway epithelium as the critical “first line of defense” and a central orchestrator of the inflammatory response [4,5]. In recent years, aberrant cellular metabolic reprogramming has emerged as a critical factor driving this tenacious inflammation [6,7].

Upon exposure to allergens such as house dust mite (HDM), airway epithelial cells undergo rapid metabolic reprogramming to meet the bioenergetic demands of host defense [8,9]. Specifically, a shift toward aerobic glycolysis (the Warburg effect) leads to the substantial accumulation of a key metabolite, lactate. This accumulation creates an acidic, proinflammatory microenvironment and establishes a self-sustaining “inflammation-metabolism” vicious cycle [6]. Therefore, elucidating the key molecular events within this cycle, particularly how lactate transcends its role as a metabolic by-product to become a precise signaling molecule regulating inflammatory programs represents a critical frontier in the field.

Lactate and succinate, once regarded merely as metabolic by-products, are now recognized as key signaling molecules, or “immunometabolites”, that regulate inflammatory and immune responses [10–12]. Beyond its role as a metabolic waste product, lactate has recently been identified as a potent signaling molecule that modulates cellular functions through metabolic intermediates and posttranslational modifications (PTMs) [13]. A critical metabolic consequence of high lactate load is the modulation of glutaminolysis, which supports biosynthetic demands by replenishing the tricarboxylic acid (TCA) cycle (TCA anaplerosis) [14,15]. One of the key products of this process is succinate, an established endogenous proinflammatory mediator that amplifies inflammatory signals [12]. Particularly relevant to our study, recent research has confirmed that gut microbiota-derived succinate is a critical pathogenic factor that exacerbates allergic airway inflammation [16]. This strongly suggests that a metabolic cascade in the asthmatic microenvironment, consisting of “lactate-driven glutaminolysis leading to succinate production”, may be a key upstream event that amplifies inflammatory signals. However, the signal transduction mechanisms—specifically, how the accumulation of these metabolites is recognized by specific intracellular molecules and translated into downstream pathophysiological functions—remain a key scientific question in the field. To understand how metabolites regulate complex cellular programs, it is essential to delve into the layers of PTMs and epigenetics [17,18]. PTMs directly driven by metabolites, such as succinylation and lactylation, represent a direct molecular mechanism by which cells sense and transduce their metabolic state [13,19]. These emerging “metabolic” PTMs have been identified as core drivers in numerous diseases, as they can directly alter protein stability, enzymatic activity, and interactions. However, the ultimate effect of these signals often manifests within the nucleus through the regulation of gene expression. Protein arginine methyltransferase 5 (PRMT5) is a key protein for understanding this process [20,21]. As a core epigenetic repressor, PRMT5 exerts a critical transcriptional repressive function by catalyzing the formation of the repressive histone mark H4R3me2s [22,23]. Yet, how the activity and targeting of PRMT5 are modulated by upstream metabolic signals, particularly in airway epithelial inflammatory diseases like asthma, remains largely unknown.

Within this complex regulatory network, some classical proteins may exert important noncanonical functions [24]. Phosphoenolpyruvate carboxykinase 2 (PCK2) is a key mitochondrial metabolic enzyme, conventionally regarded as the central hub connecting the TCA cycle and gluconeogenesis [25]. Its precise localization within the mitochondrial matrix places it at a critical metabolic crossroads, and it utilizes TCA cycle intermediates derived from pathways like glutaminolysis. This positioning provides a molecular basis for its noncanonical functions. While PCK2 is known to regulate gluconeogenesis, its specific role in sensing lactate stress and modulating airway inflammation is largely unexplored. Likewise, polyglutamine-binding protein 1 (PQBP1) is a multifunctional protein best known for its interaction with pathogenic proteins in neurodegenerative diseases and its involvement in transcriptional regulation and pre-mRNA splicing [26,27]. However, whether these proteins can act as signaling nodes in the regulation of immunometabolism has never been reported.

Consequently, a core challenge in the field is to delineate a complete signaling pathway that interlinks metabolic reprogramming, PTMs, and epigenetic regulation. Based on this key scientific question, we proposed and validated a central hypothesis: In the asthmatic inflammatory microenvironment, a self-reinforcing lactate positive feedback loop mediated by the metabolic enzyme PCK2 exists. The lactate signal produced by this loop acts on a key signal transducer protein, PQBP1, through a novel PTM, lactylation. Modified PQBP1, in turn, directly modulates the function of the core epigenetic repressive complex PRMT5, ultimately lifting the transcriptional repression of downstream proinflammatory genes. This study aims to systematically elucidate this complete “metabolism-modification-epigenetics” signaling cascade, thereby providing a new molecular paradigm for understanding the chronic inflammatory mechanisms of asthma and offering precise targets for the development of metabolism-based therapeutic strategies.

## Results

### Lactate–succinate metabolic coupling promotes airway inflammation in asthma

**Fig. 1.**
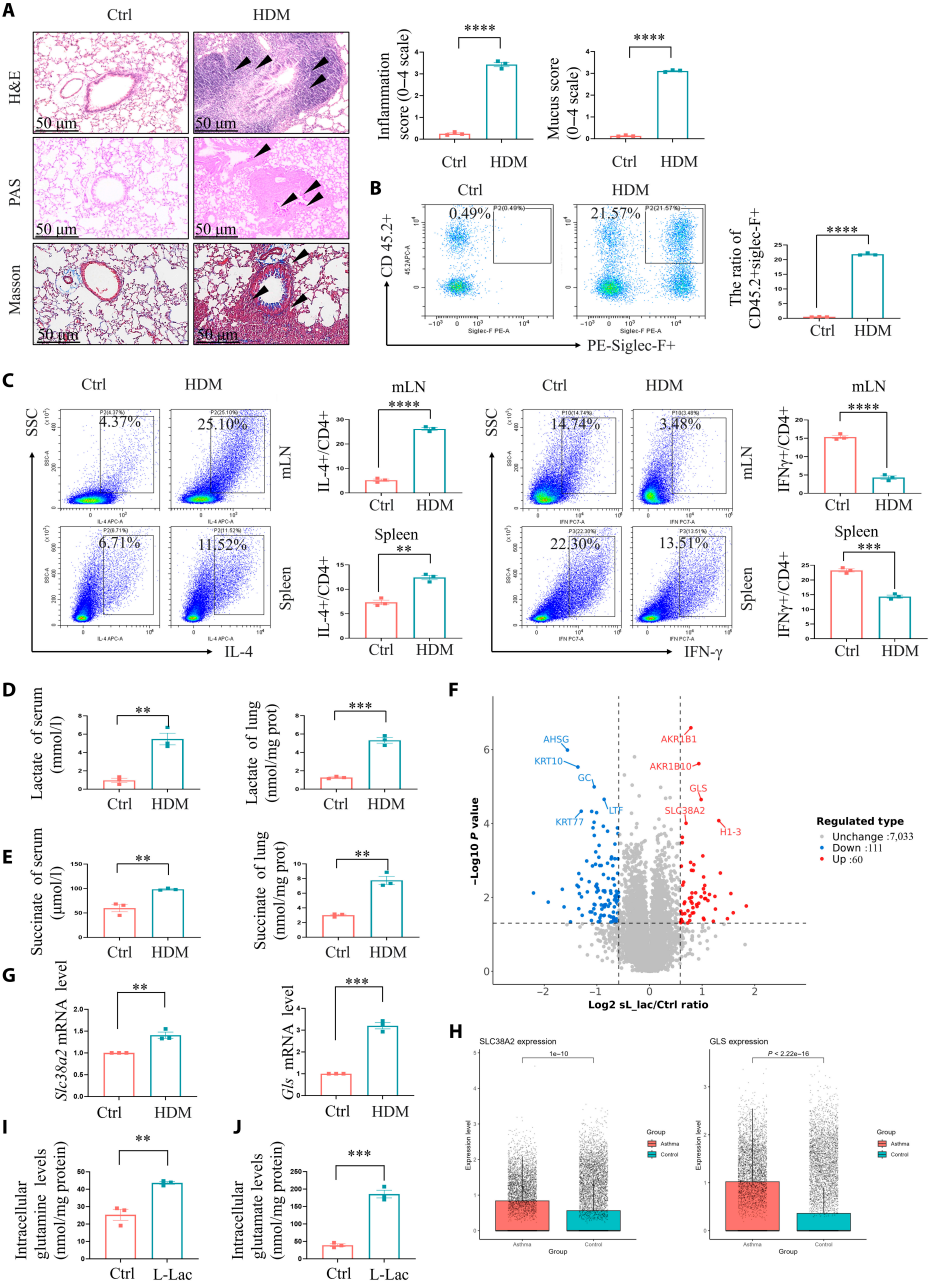
Metabolic reprogramming and airway inflammation occur in the house dust mite (HDM)-induced asthma model. (A) Representative images of hematoxylin and eosin (H&E), periodic acid-Schiff (PAS), and Masson trichrome staining of lung tissues from control (Ctrl) and HDM-challenged mice. Arrows indicate inflammatory cell infiltration, mucus hypersecretion, and subepithelial fibrosis (collagen deposition). Scale bar, 50 μm. (B and C) Flow cytometric quantification of eosinophils in the bronchoalveolar lavage fluid (BALF) (CD45.2^+^ CD11c^−^ Siglec-F^+^) (B) and T helper 1 (Th1)/Th2 cells in the lymph nodes and spleen (C) of the 2 groups. (D and E) Concentrations of lactate (D) and succinate (E) in the serum and lung homogenates of the 2 groups. (F) Volcano plot from the quantitative proteomic analysis of lactate-stimulated BEAS-2B cells. (G) *Slc38a2/Gls* mRNA levels in lung tissues (quantitative polymerase chain reaction [qPCR]). (H) Single-cell RNA sequencing (scRNA-seq) analysis (GSE193816) confirming the significant up-regulation of *SLC38A2* and *GLS* in asthmatic airway epithelial cells (*P* values are indicated, Wilcoxon rank-sum test). (I and J) Quantification of intracellular glutamine and glutamate levels in lactate-treated BEAS-2B cells. Intracellular concentrations of glutamine (I) and glutamate (J) were measured using colorimetric assays and normalized to total protein content (nmol/mg protein). Data are presented as means ± standard error of the mean (SEM) (*n* = 6 to 8 per group). Analyzed by Student *t* test. **P < 0.01, ***P < 0.001, ****P < 0.0001. mLN, mediastinal lymph node.

To investigate the central role of metabolic reprogramming in asthma pathology, we established an HDM-induced mouse model of allergic airway inflammation. This model successfully recapitulated the key pathological features of asthma, including marked inflammatory cell infiltration in lung tissues, as well as periodic acid-Schiff (PAS)-positive goblet cell hyperplasia and mucus hypersecretion, as well as marked subepithelial fibrosis indicated by Masson trichrome staining (Fig. 1A). Concurrently, the eosinophil count in the bronchoalveolar lavage fluid (BALF) was dramatically increased (Fig. 1B), consistent with this inflammatory cell influx, enzyme-linked immunosorbent assay (ELISA) analysis of the BALF supernatant revealed a robust cytokine storm, characterized by significantly elevated levels of type 2 (interleukin-4 [IL-4], IL-5, and IL-13) and type 17 (IL-17) inflammatory cytokines (Fig. S1A). This local immune activation was further accompanied by a significant elevation of the Th2 cytokine (IL-4) and a reduction of the Th1 cytokine (interferon-γ [IFN-γ]) in the lymph nodes and spleen (Fig. 1C).

In this model, we measured key metabolites and found that the concentrations of lactate and succinate were significantly higher in the lung tissues and serum of HDM-challenged mice compared to the control group (Fig. 1D and E). This result reveals a metabolic dysregulation in the asthmatic inflammatory microenvironment characterized by the synergistic and abnormal accumulation of lactate and succinate. Given that succinate is a key product of glutaminolysis, we hypothesized that lactate might indirectly promote succinate accumulation by modulating glutamine metabolism.

To validate this hypothesis at the molecular level and explore the specific mechanism of lactate’s regulatory role, we integrated multidimensional bioinformatics analysis (using the Comparative Toxicogenomics Database and GeneCards database) with quantitative proteomic analysis of lactate-stimulated human airway epithelial cells (BEAS-2B). The results from both approaches converged on glutaminase (GLS) and the glutamine transporter solute carrier family 38 member 2 (SLC38A2) as top candidate genes (Fig. 1F and Fig. S1B). Pathway enrichment analysis further confirmed that GLS and SLC38A2 were highly enriched in glutamine/glutamate metabolism-related pathways (Fig. S1C and D).

Subsequent in vitro experiments validated this regulatory relationship. Crucially, to confirm the relevance of these targets in the context of allergen-induced pathology, we first treated BEAS-2B cells directly with HDM. Real-time quantitative polymerase chain reaction (RT-qPCR) analysis revealed a significant up-regulation of both *Slc38a2* and *Gls* mRNA levels (Fig. 1G), confirming their responsiveness to the primary disease trigger. To further elucidate the metabolic hierarchy, we examined the protein and metabolite profiles following HDM stimulation. As shown in Fig. S1E to H, HDM acted as a potent trigger for a “Warburg-like” glycolytic switch, evidenced by the significant up-regulation of hexokinase 2 (HK2) and lactate dehydrogenase A protein levels and a marked increase in intracellular lactate production. This initial glycolytic burst was accompanied by the activation of the downstream glutaminolysis pathway, characterized by the up-regulation of the glutamine transporter SLC38A2 and GLS, ultimately leading to significant intracellular succinate accumulation. Moreover, single-cell RNA sequencing (scRNA-seq) analysis of clinical samples (GSE193816) further corroborated the specific up-regulation of *SLC38A2* and *GLS* in asthmatic airway epithelium (Fig. 1H). Consistent with this finding, lactate stimulation significantly up-regulated the protein and mRNA expression levels of *SLC38A2* and *GLS* in BEAS-2B cells (Fig. S2A and B) and enhanced GLS enzymatic activity (Fig. S2C). Functionally, lactate stimulation led to a significant increase in intracellular pH (pHi), which is consistent with the activation of the SLC38A2 transporter (Fig. S2D). To exclude osmotic stress, BEAS-2B cells were treated with 10 mM sodium lactate or equimolar NaCl for 24 h. Unlike lactate, which significantly up-regulated Pan-Kla and SLC38A2, NaCl treatment failed to induce these changes (Fig. S2E). This confirms that the observed alterations are driven specifically by lactate, not by osmolarity or ionic strength. Functionally, direct metabolite quantification confirmed that lactate stimulation significantly elevated intracellular glutamine and glutamate levels (Fig. 1I and J), validating the enhanced glutamine uptake and glutaminolysis flux. Untargeted metabolomics analysis confirmed that lactate treatment ultimately resulted in a significant increase in intracellular succinate levels (Fig. S2F and G). To establish a causal link for the SLC38A2/GLS pathway, we performed intervention experiments using short hairpin RNA (shRNA)-mediated knockdown or overexpression. Prior to functional assays, the efficiency of these genetic manipulations was verified by Western blotting, which showed significant reduction or elevation of SLC38A2 and GLS protein levels as expected (Fig. S2H and I). The results showed that knocking down either *SLC38A2* or *GLS* significantly inhibited lactate-induced succinate accumulation and the increase in the extracellular acidification rate (ECAR) (Fig. S3A to C), whereas overexpressing *SLC38A2* or *GLS* mimicked the effects of lactate (Fig. S3D to F).

Taken together, the results from this section systematically reveal that in the asthmatic microenvironment, lactate can promote glutaminolysis by activating the SLC38A2/GLS signaling pathway, thereby leading to the accumulation of succinate.

### Mitochondrial dysfunction contributes to selective PCK2 succinylation

**Fig. 2.**
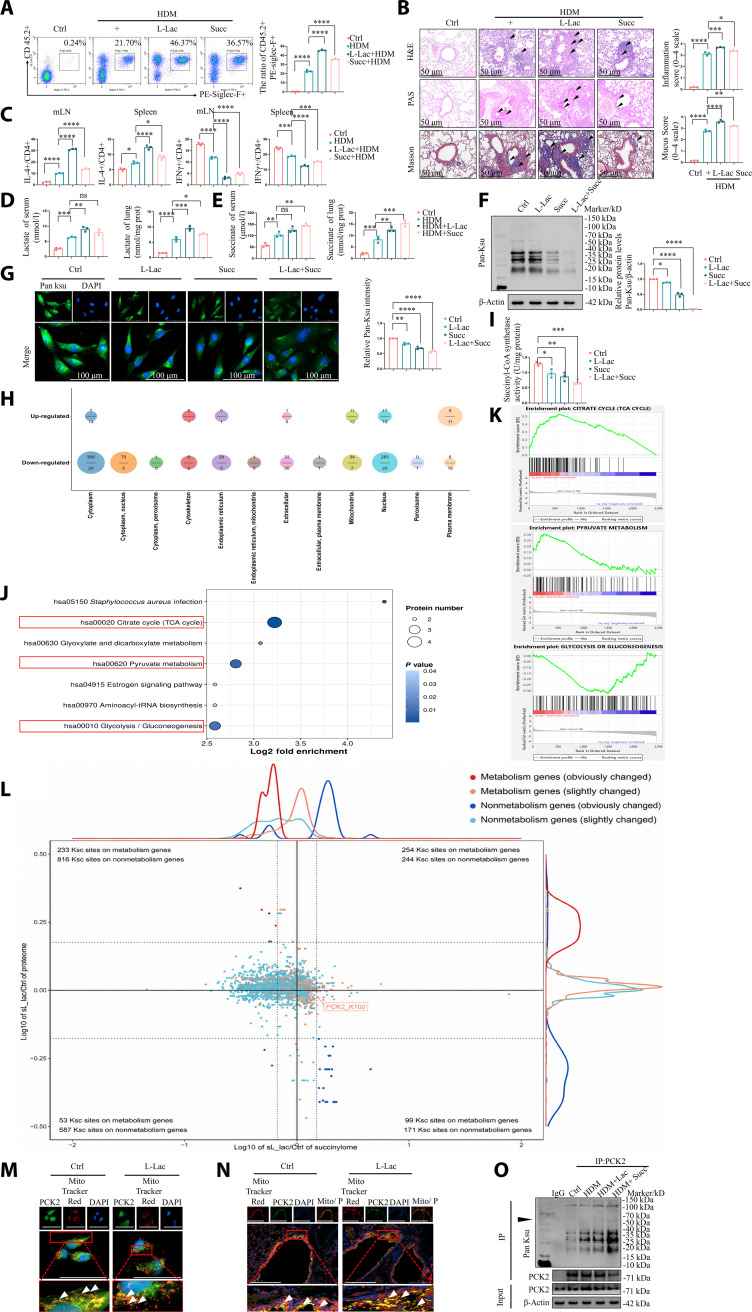
The “succinate paradox” points to mitochondrial dysfunction and identifies phosphoenolpyruvate carboxykinase 2 (PCK2) as a key protein. (A to E) Effects of exogenous supplementation with Lactate (L-Lac) or succinate (Succ) on airway inflammation in the house dust mite (HDM) model, showing analysis of bronchoalveolar lavage fluid (BALF) eosinophils (CD45.2^+^ CD11c^−^ Siglec-F^+^) (A), lung tissue hematoxylin and eosin (H&E), periodic acid-Schiff (PAS) and Masson trichrome staining (B), lymph node and spleen T helper 1 (Th1)/Th2 cells (C), and serum/lung lactate and succinate concentrations (D and E). Scale bar, 50 μm. (F and G) Western blot (F) and immunofluorescence (G) analysis of global protein succinylation (Pan-Ksu) in BEAS-2B cells treated with lactate. Quantitative analysis of relative band intensity (F) and mean fluorescence intensity (MFI) (G) is shown in the right panels. Scale bar, 100 μm. (H, J, K, and L) Proteomic analysis of differentially succinylated proteins (DEKPs), including subcellular localization (H), Kyoto Encyclopedia of Genes and Genomes (KEGG) pathway enrichment (J), Gene Set Enrichment Analysis (GSEA) (K), and the Integrated Proteome-Succinylome Association Plot that identified PCK2 (L). (I) Effect of lactate treatment on intracellular succinyl-coenzyme A synthetase (SCS) activity. (M and N) Immunofluorescence showing the localization of PCK2 (green) and mitochondria (red) in lactate-treated BEAS-2B cells (M) and in the airway epithelium of HDM-challenged mice (N). Scale bars, 40 and 200 μm, respectively. (O) Immunoprecipitation (IP) of PCK2 from mouse lung tissues followed by Western blot for succinylation. All Western blots are representative of ≥3 independent experiments. Data are presented as means ± standard error of the mean (SEM) (*n* = 6 to 8 per group for in vivo, *n* = 3 independent experiments for in vitro). Analyzed by 1-way analysis of variance (ANOVA). *P < 0.05, **P < 0.01, ***P < 0.001, ****P < 0.0001. ns, not significant.

To investigate the in vivo functions of lactate and succinate accumulation, we exogenously supplemented these metabolites in HDM-challenged mice (Fig. S4A). We found that both compounds further exacerbated airway inflammation, goblet cell hyperplasia, and Th2 immune deviation. Specifically, ELISA analysis of BALF confirmed that lactate or succinate supplementation significantly potentiated the cytokine storm, leading to even higher levels of IL-4, IL-5, IL-13, and IL-17 compared to the HDM-only group (Fig. S4B). To assess whether these pathological changes translated into physiological dysfunction, we measured airway hyperresponsiveness (AHR). The results showed that supplementation with either lactate or succinate significantly exacerbated methacholine-induced airway resistance compared to the HDM-only group (Fig. S4C). Notably, this supplementation led to a consistent and even greater accumulation of lactate and succinate in both lung tissues and serum (Fig. 2A to E and Fig. S4D and E), confirming their direct roles in perpetuating the inflammatory vicious cycle.

However, while investigating the underlying molecular mechanism, we uncovered that mitochondrial dysfunction uncouples succinate accumulation from protein succinylation: Despite elevated intracellular succinate levels, the global protein succinylation in lactate-stimulated cells was, contrary to expectations, generally down-regulated (Fig. 2F and G). Notably, this distinct “uncoupling” phenotype stands in sharp contrast to the global hyper-succinylation observed in the lipopolysaccharide-induced general mitochondrial injury model (Fig. S4F), highlighting the metabolic specificity of the lactate-enriched asthmatic microenvironment. To systematically validate this phenomenon, we performed succinyl-proteomics analysis. Quality control assessment of the data showed good biological reproducibility among the experimental sample groups, ensuring the reliability of subsequent analyses (Fig. S4G to I). Among the 800 identified differentially succinylated sites, the vast majority showed significant down-regulation, a finding in high concordance with our Western blot results (Fig. S4J and K). Sequence motif, “hyper-succinylation”, and subcellular localization analyses revealed that these down-regulated modifications primarily occurred on proteins associated with core energy metabolism pathways within the cytoplasm (Fig. 2H and Fig. S4L and M).

To elucidate the mechanism driving this global down-regulation, we first investigated whether it was caused by increased enzymatic removal. We examined the expression of sirtuin 5 (SIRT5), the primary mitochondrial desuccinylase, and found no significant difference in protein levels between lactate-treated and control groups (Fig. S5A). This ruled out desuccinylase up-regulation as a contributing factor.

A contributing factor to this phenomenon may be related to the limited availability of succinyl-coenzyme A (succinyl-CoA), the direct donor for lysine succinylation. We found that lactate stimulation significantly reduced the activity of succinyl-CoA synthetase (SCS) (Fig. 2I), and this inhibitory effect was further exacerbated by Su treatment as well as the combined L-Lac+Su treatment. Given that the direct donor required for lysine succinylation is succinyl-CoA (rather than succinate itself), these results suggest that lactate-induced metabolic dysfunction may impair the energy conversion steps associated with the TCA cycle, thereby reducing the intracellular availability of succinyl-CoA and ultimately leading to a global decline in succinylation levels. Subsequent experiments confirmed that lactate treatment led to a reprogramming of cellular energy metabolism, manifested as impaired mitochondrial oxidative phosphorylation (reduced oxygen consumption rate [OCR]) with a slight increase in glycolysis (increased ECAR), a decreased mitochondrial membrane potential (ΔΨm), damaged mitochondrial morphology (disrupted cristae structures), and significantly elevated mitochondrial reactive oxygen species (mtROS) (Fig. S5B to F). Concurrently, the intracellular level of the oxidative damage marker malondialdehyde (MDA) increased, while antioxidants such as reduced glutathione (GSH), catalase (CAT), and superoxide dismutase (SOD) were depleted (Fig. S5G).

Against this backdrop of metabolic reprogramming, we performed pathway enrichment analysis and Gene Set Enrichment Analysis (GSEA) on the differentially succinylated proteins. We found that they were significantly enriched in core energy metabolism pathways, including glycolysis/gluconeogenesis, the TCA cycle, and pyruvate metabolism (Fig. 2J and K). To pinpoint the key “effector” among the globally down-regulated succinylated proteins, we performed an Integrated Proteome-Succinylome Association Plot. As a result, PCK2 was successfully identified: Its succinylation at the K100 site was, in contrast to the global trend, specifically and significantly up-regulated, while its total protein level slightly decreased (Fig. 2L). Mass spectrometry data further confirmed the significant up-regulation of PCK2-K100 succinylation (Fig. S5H and I).

To further investigate the cell biology context of the identified key protein PCK2, we observed its subcellular localization via immunofluorescence. In control BEAS-2B cells, PCK2 (green fluorescence) perfectly colocalized with the intact, filamentous mitochondrial network (red fluorescence). Under lactate stimulation, however, PCK2 remained localized within the now severely fragmented mitochondrial network (Fig. 2M). This finding was also confirmed in vivo, where PCK2 was similarly localized to fragmented mitochondria in the airway epithelial cells of HDM-asthmatic mice (Fig. 2N). These results visually linked the key protein PCK2 with the lactate-induced mitochondrial pathological phenotype.

Finally, through immunoprecipitation (IP) experiments on mouse lung tissue samples, we confirmed in vivo that exogenous supplementation of either lactate or succinate significantly increased the specific succinylation of PCK2 (Fig. 2O), thereby establishing PCK2 as the key protein responsive to the succinate signal.

### PCK2-K100 succinylation and lactate metabolism

**Fig. 3.**
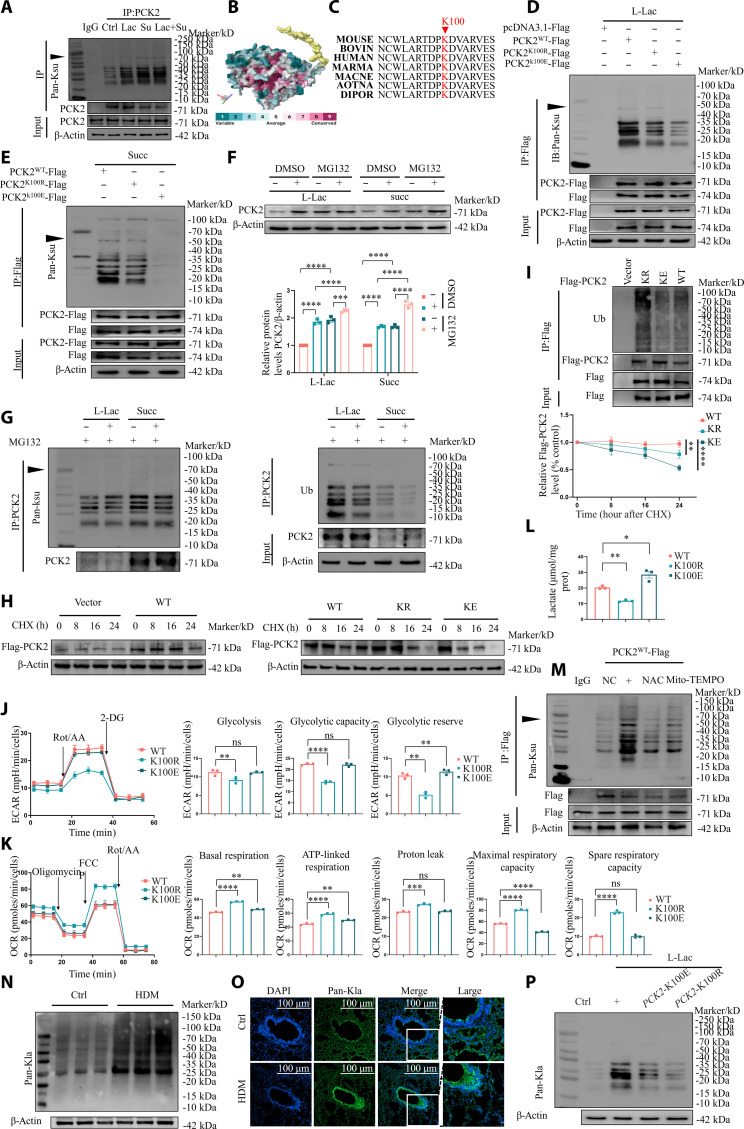
Phosphoenolpyruvate carboxykinase 2 (PCK2)-K100 succinylation drives a lactate positive feedback loop via protein stabilization. (A) Immunoprecipitation (IP) of PCK2 from BEAS-2B cells to detect succinylation induced by lactate (Lac) or succinate (Su). (B and C) Evolutionary conservation analysis of the PCK2-K100 site, showing a structural surface conservation score map (B) and multispecies sequence alignment (C). (D and E) Detection of exogenous PCK2 succinylation induced by lactate (D) or succinate (E) in cells expressing wild-type (WT) or mutant (K100R/E) PCK2. (F) Effect of lactate or succinate on PCK2 protein levels in the presence of MG132. (G) Coimmunoprecipitation (Co-IP) of PCK2 to detect changes in its succinylation and ubiquitination upon lactate or succinate treatment. (H) Cycloheximide (CHX) chase assay comparing the degradation rates of WT and mutant (KR/KE) PCK2. (I) Co-IP analysis of the ubiquitination levels of WT and mutant PCK2. (J and K) Seahorse analysis of extracellular acidification rate (ECAR) (J) and oxygen consumption rate (OCR) (K) in cells expressing WT or mutant PCK2. (L) Lactate production in cells expressing different PCK2 mutants. (M) Detection of lactate-induced PCK2 succinylation in cells overexpressing Flag-PCK2 following pretreatment with antioxidants (N-acetylcysteine [NAC]/Mito-TEMPO). (N and O) Western blot (N) and immunofluorescence (O) analysis of global protein lactylation (Pan-Kla) in lung tissues of house dust mite (HDM)-challenged mice. Scale bar, 100 μm. (P) Effect of overexpressing WT or mutant PCK2 on global lactylation levels. Note: Multiple bands present in the pan-succinylation blots correspond to ubiquitinated species and partial degradation fragments of PCK2, rather than nonspecific interacting proteins. All Western blots are representative of ≥3 independent experiments. Data are presented as means ± standard error of the mean (SEM) (*n* = 3 independent experiments). Analyzed by 1 or 2-way analysis of variance (ANOVA). *P < 0.05, **P < 0.01, ***P < 0.001, ****P < 0.0001; ns, not significant.

To investigate the function of PCK2-K100 succinylation, we confirmed at the cellular level via immunoprecipitation (IP) that exogenous supplementation with either lactate or succinate directly induces the succinylation of the PCK2 protein (Fig. 3A). Structural and sequence analyses revealed that the K100 residue is exposed on the protein surface and is highly conserved across species (Fig. 3B and C), implying its significant functional potential. By constructing site-directed mutants, K100R (arginine, to block succinylation) and K100E (glutamate, to mimic the negative charge of constitutive succinylation), we demonstrated that K100 is the core site that undergoes succinylation in response to lactate and succinate signals (Fig. 3D and E).

We hypothesized that succinylation at this site is critical for PCK2 protein stability. Experiments confirmed that PCK2 is degraded primarily through the ubiquitin-proteasome system (UPS) (Fig. S6A). Building on this, we found that supplementation with exogenous lactate or succinate further enhanced the accumulation of PCK2 protein induced by the proteasome inhibitor MG132 (Fig. 3F), indicating that these metabolites inhibit PCK2 degradation. To exclude the potential confounding effect of MG132-induced metabolic stress, we verified that although MG132 treatment caused a minor elevation in basal intracellular lactate, this increase was negligible compared to the robust accumulation driven by the exogenous supplementation used in our assays (Fig. S6B). This confirms that the observed protein stabilization is specific to the exogenous metabolite signals rather than nonspecific drug toxicity. To explore the molecular mechanism, we performed coimmunoprecipitation (Co-IP) analysis and confirmed that lactate or succinate supplementation significantly reduced the overall ubiquitination level of PCK2 (Fig. 3G). To directly assess the function of K100 succinylation, we conducted a cycloheximide (CHX) chase assay. The results showed that the degradation rate of wild-type (WT) PCK2 was significantly slower than that of the K100R/E mutants, which cannot be succinylated (Fig. 3H). Consistent with this, Co-IP experiments revealed that the ubiquitination levels of the K100R/E mutants were significantly higher than those of the WT (Fig. 3I). Taken together, these results demonstrate that succinylation at the PCK2-K100 site effectively antagonizes its ubiquitination-dependent proteasomal degradation, thereby significantly enhancing PCK2 protein stability.

This succinylation-enhanced protein stability, coupled with intrinsic enzymatic activation, dictates a fundamental shift in PCK2 function against the backdrop of mitochondrial dysfunction in asthmatic inflammation. To directly validate this catalytic gain of function, we performed an in vitro PEPCK enzymatic assay using purified Flag-tagged proteins. The K100E mutant (mimicking succinylation) exhibited significantly higher enzymatic activity compared to the WT and K100R mutants (Fig. S6C and D). Consistently, measurement of lactate output in the culture medium confirmed that cells expressing the K100E mutant drove significantly higher lactate production compared to the WT and K100R groups (Fig. S6E). These biochemical data provide direct evidence that K100 succinylation enhances PCK2’s catalytic efficiency and drives metabolic flux toward lactate generation. Seahorse metabolic analysis showed that cells overexpressing PCK2-WT or K100E exhibited a higher ECAR and lower mitochondrial respiration (OCR) and adenosine triphosphate production rates compared to cells with the K100R mutant (Fig. 3J and K). Further functional experiments also confirmed that PCK2-K100 succinylation not only led to a decrease in mitochondrial membrane potential and damaged morphology but also significantly exacerbated intracellular oxidative stress (increased reactive oxygen species [ROS] and MDA; decreased GSH, CAT, and SOD) (Fig. S7A to E).

This indicates that in the context of mitochondrial dysfunction, stabilized PCK2 undergoes functional adaptation. It no longer performs its classical gluconeogenic function but instead appears to favor its “cataplerotic” function, converting TCA cycle carbon skeletons derived from glutaminolysis into phosphoenolpyruvate (PEP), which is then efficiently converted to lactate via the lower half of glycolysis. These findings revealed a self-reinforcing positive feedback loop: Initial lactate accumulation induces succinate production, leading to PCK2-K100 succinylation; this stabilizes PCK2 and shifts its function to efficiently generate more lactate. Experiments confirmed that, compared to WT or K100E, the K100R mutation significantly reduced the cell’s capacity for lactate production (Fig. 3L). To rigorously confirm the exact directionality of this loop and exclude the possibility that succinate merely drives lactate production through the classical hypoxia-inducible factor-1α pathway, we evaluated the PCK2 dependency of this process. Pharmacological inhibition of glutaminolysis with the GLS inhibitor bis-2-(5-phenylacetamido-1,3,4-thiadiazol-2-yl)ethyl sulfide (BPTES) significantly blunted HDM-induced lactate accumulation, confirming that succinate generation is a prerequisite for sustaining high lactate levels (Fig. S7G). Furthermore, while direct supplementation with exogenous succinate increased intracellular lactate, this effect was significantly abrogated when endogenous *PCK2* was knocked down (Fig. S7H). These results clearly establish that the succinate-driven lactate accumulation in our model is heavily reliant on the newly identified PCK2 axis rather than being solely driven by classical reverse regulation.

The substantial accumulation of lactate in the cellular microenvironment driven by PCK2 is not just a result of metabolic reprogramming; it may also act as a signaling molecule to further influence the cellular redox state. We found that after lactate stimulation, intracellular ROS levels showed a time-dependent and significant increase (Fig. S7F). We demonstrated that inhibiting oxidative stress could interrupt this positive feedback loop. Treatment with the antioxidants N-acetylcysteine or Mito-TEMPO not only significantly reduced lactate-induced lactate production and ECAR (Fig. S8A to C) but also, in PCK2-overexpressing cells, significantly inhibited PCK2 succinylation and ECAR (Fig. 3M and Fig. S8D). This reveals that oxidative stress acts as a critical amplifier in the PCK2-lactate positive feedback loop: Lactate enhances its own production via PCK2 and induces oxidative stress, which in turn promotes PCK2 succinylation, thereby further reinforcing this vicious cycle.

The abundant lactate generated by this loop provides an ample signaling substrate for widespread downstream protein lactylation. Experiments confirmed that in both the HDM asthma model and the in vitro cell models, lactate accumulation induced a significant increase in global protein lysine lactylation (pan-Kla), and this modification was predominantly enriched in the nucleus (Fig. 3N and O and Fig. S8E and F). Crucially, when the PCK2-K100 site was mutated, this lactate-induced global lactylation effect was significantly attenuated (Fig. 3P). These results collectively indicate that the PCK2-driven lactate positive feedback loop triggers widespread protein lactylation, primarily occurring in the nucleus. Further functional experiments also confirmed that inhibiting PCK2 or its K100 site function could comprehensively reverse the glycolytic phenotype induced by lactate (Fig. S9A to D). More importantly, to definitively establish the causal link between PCK2-K100 succinylation and downstream signaling, we performed a genetic rescue experiment. Under HDM stimulation, silencing endogenous *PCK2* almost completely abolished PQBP1 lactylation. Reconstitution with *PCK2*-WT fully rescued this modification, whereas the succinylation-deficient K100R mutant failed to restore PQBP1 lactylation (Fig. S9E). This demonstrates that the specific succinylation of PCK2 at K100 is an indispensable prerequisite for driving downstream PQBP1 lactylation.

### Quantitative lactyl-proteomics identifies PQBP1 as a key lactate-responsive protein

To comprehensively identify one of the central regulator proteins responding to the lactate signal, we employed 4-dimensional label-free quantification quantitative lactyl-proteomics. The quality control results demonstrated good experimental reproducibility (Fig. S10A to C), providing a solid foundation for subsequent analyses. Volcano plot analysis indicated that lactate stimulation triggered widespread and specific lactylation events, with as many as 1,374 of the 1,485 identified differentially lactylated sites showing an upward trend (Fig. S10D and E).

**Fig. 4.**
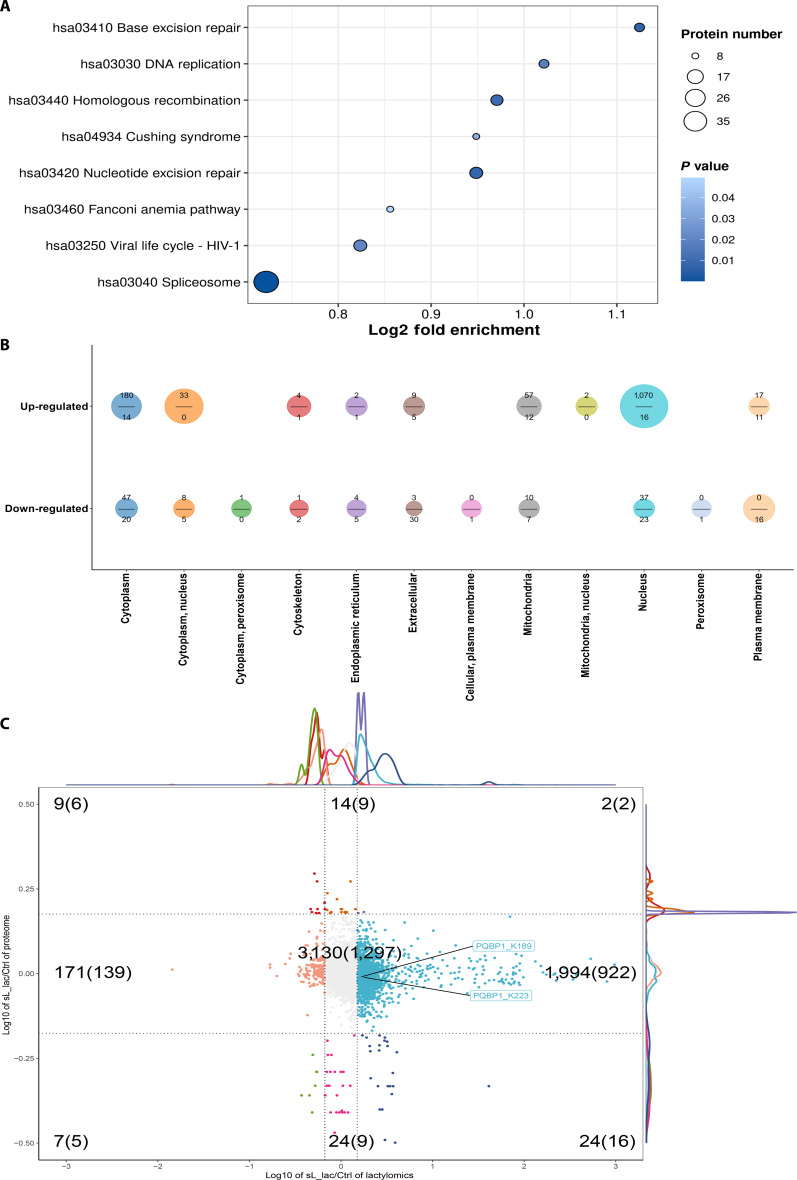
Lactyl-proteomics identifies polyglutamine-binding protein 1 (PQBP1) as a key effector molecule. (A and B) Kyoto Encyclopedia of Genes and Genomes (KEGG) pathway enrichment analysis (A) and subcellular localization analysis (B) of differentially lactylated proteins. (C) Integrated Proteome-Lactylome Association Plot of the lactylome and proteome.

To investigate the biological significance of this global change, we performed bioinformatics analysis on the differentially lactylated proteins. Kyoto Encyclopedia of Genes and Genomes (KEGG) pathway enrichment analysis revealed that these proteins were significantly enriched in the spliceosome pathway (Fig. 4A). GSEA not only confirmed the enrichment of this pathway but also uncovered the enrichment of several immune-inflammatory pathways relevant to asthma pathology, such as the IL-17 signaling pathway (Fig. S10F and G). Subcellular localization analysis further confirmed that these proteins were predominantly enriched in the nucleus (Fig. 4B), which is highly consistent with the functional localization of the spliceosome. Additionally, analysis of the features of the differential sites revealed conserved sequence motifs and a “hyper-lactylation” phenomenon (Fig. S10H and I). Taken together, these bioinformatics analyses collectively suggested that the lactate signal may regulate RNA splicing and immune-inflammatory processes by driving the lactylation of key proteins within the nucleus, particularly those in the spliceosome pathway.

To unbiasedly pinpoint the most critical effector protein from a multitude of candidates, we employed a pathway-based hierarchical filtering strategy. First, through dual-omics intersection (lactyl-proteomics versus global proteomics), we excluded false positives caused by protein abundance fluctuations, identifying 752 high-confidence targets whose altered lactylation was purely driven by modification levels (Fig. S11A). Second, building upon the pronounced enrichment of the RNA processing and spliceosome pathways within these common targets (Fig. S11B and C and Fig. 4A), we quantitatively ranked the 42 candidate proteins within this core functional network. Consequently, PQBP1 emerged as a top-tier candidate: Its specific lactylation level was highly significantly up-regulated (Top 4, *P* = 0.000076), while its total protein level remained strictly unchanged in the Integrated Proteome-Lactylome Association Plot (Fig. 4C). Crucially, given its exclusive dual role in both RNA processing and core epigenetic regulation among these top candidates, PQBP1 perfectly aligned with our theoretical rationale for a sensor bridging metabolic signaling and nuclear transcription. This data-driven and mechanistically exclusive screening made PQBP1 an ideal candidate for mediating the lactate signal, and we therefore focused our subsequent research on its function and mechanism.

### PQBP1-K223 lactylation drives inflammation through PRMT5-mediated epigenetic reprogramming

**Fig. 5.**
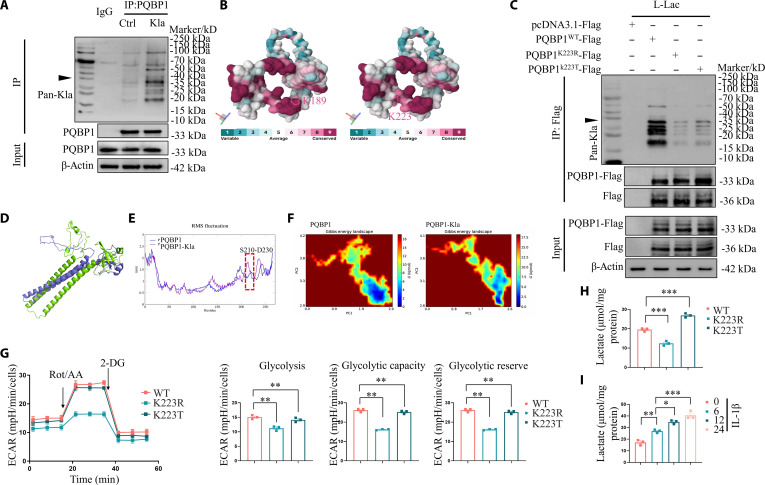
Polyglutamine-binding protein 1 (PQBP1)-K223 lactylation mediates inflammation and metabolic reprogramming. (A) Immunoprecipitation (IP) of endogenous PQBP1 to detect the effect of lactate stimulation on its lactylation (Kla). (B) Evolutionary conservation analysis of PQBP1, with K189 and K223 sites highlighted. (C) Detection of exogenous PQBP1 lactylation induced by lactate in cells expressing wild-type (WT) or mutant (K223R/T) PQBP1. (D to F) Molecular dynamics simulation analysis of the effect of K223 lactylation on PQBP1 protein conformation, including backbone structure (D), RMSF (E), and Gibbs free energy landscape (F). (G and H) Measurement of extracellular acidification rate (ECAR) (G) and lactate production (H) in cells expressing WT, K223R, or K223T mutant PQBP1. (I) Time-course analysis of Effect of interleukin-1β (IL-1β) on intracellular lactate levels in BEAS-2B cells. Note: Multiple bands present in the pan-lactylation blots correspond to ubiquitinated species and partial degradation fragments of PQBP1, rather than nonspecific interacting proteins. All Western blots are representative of ≥3 independent experiments. Data are presented as means ± standard error of the mean (SEM) from ≥3 independent experiments. Analyzed by analysis of variance (ANOVA). *P < 0.05, **P < 0.01, ***P < 0.001.

We discovered that under stimulation with either lactate or the inflammatory cytokine IL-1β [28]—which itself can induce lactate accumulation—the lactylation level of PQBP1 was significantly elevated (Fig. 5A and Fig. S12A). We then identified the key modification site as the highly conserved K223 residue (Fig. 5B and Fig. S12B to D). By overexpressing WT and K223R mutant plasmids, we confirmed that K223 is the primary site of lactylation in response to the lactate signal, as the K223R mutation completely blocked lactate-induced PQBP1 lactylation (Fig. 5C). Molecular dynamics simulations further revealed that lactylation at this site specifically increases the conformational flexibility of the surrounding region (Fig. 5D to F and Fig. S12E to H), which may facilitate its interaction with other proteins.

Functionally, lactylation of PQBP1 at the K223 site is essential for its ability to drive glycolysis. Compared to cells overexpressing WT PQBP1, cells expressing the K223R mutant exhibited not only significantly lower basal ECAR and lactate production (Fig. 5G and H and Fig. S12I and J), but also a complete blockade of the enhanced glycolytic effect driven by the inflammatory cytokine IL-1β (Fig. 5I and Fig. S12K to N). These results indicate that PQBP1, through lactylation at its K223 site, acts as a key node that translates inflammatory signals into metabolic reprogramming.

### Lactylated PQBP1 inhibits PRMT5, reprogramming transcription through H4R3me2s regulation

**Fig. 6.**
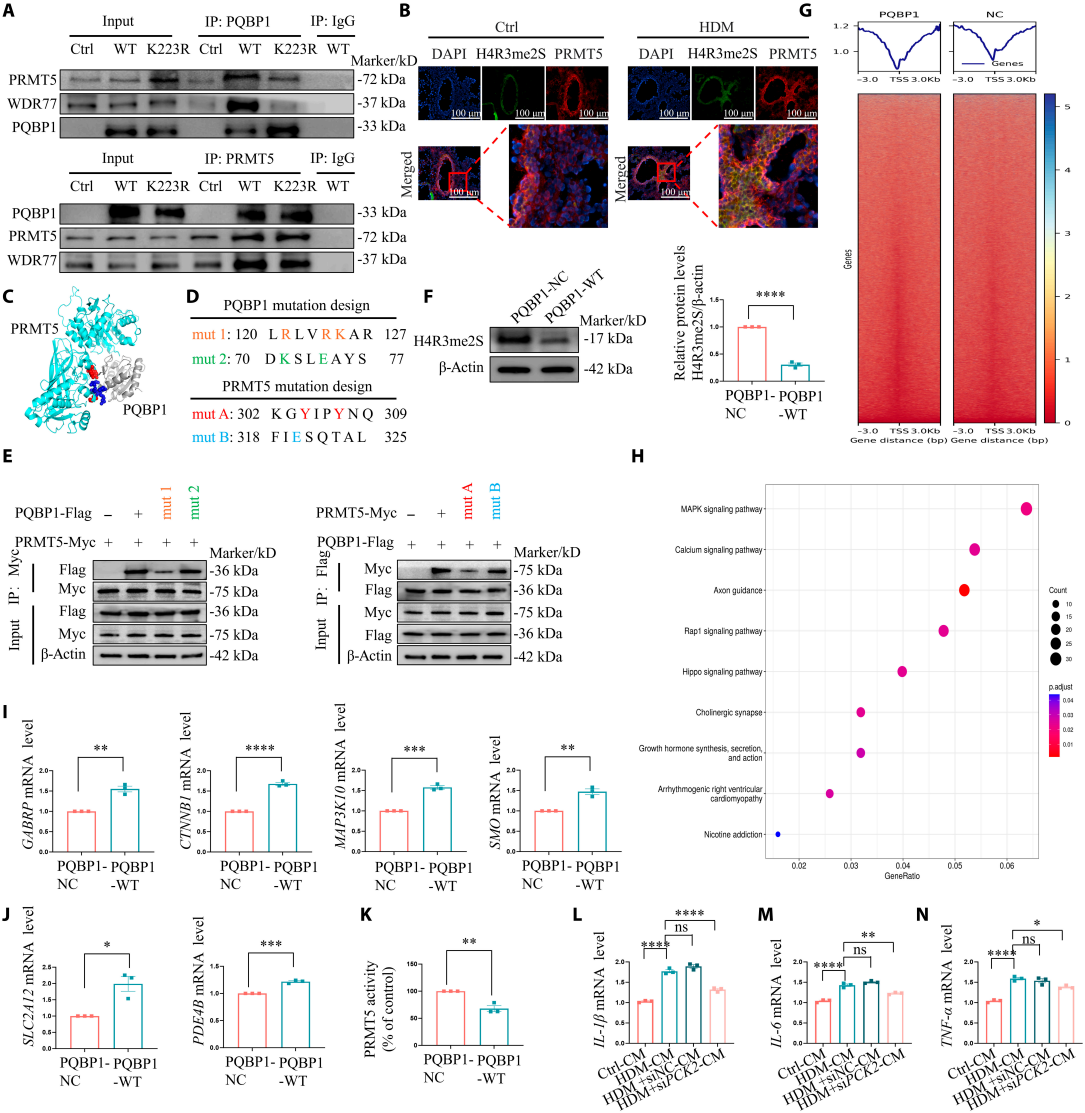
Lactylated polyglutamine-binding protein 1 (PQBP1) reprograms the epigenome and transcriptome by inhibiting the protein arginine methyltransferase 5 (PRMT5) complex. (A) Coimmunoprecipitation (Co-IP) analysis of the interaction between wild-type (WT) or K223R mutant PQBP1 and PRMT5/WD repeat domain 77 (WDR77). (B) Immunofluorescence showing the colocalization of H4R3me2S and PRMT5 in lung tissues of house dust mite (HDM)-challenged mice. Scale bar, 100 μm. (C) Molecular docking model illustrating the interaction interface between PRMT5 (light cyan) and PQBP1 (gray). (D) Schematic diagram of the PRMT5 and PQBP1 mutants designed based on the docking results. (E) Co-IP assays showing the binding affinity between the PRMT5 and PQBP1 mutants. (F) Western blot analysis of global H4R3me2s levels in cells overexpressing PQBP1-WT versus a negative control (NC). (G to J) H4R3me2s chromatin immunoprecipitation (ChIP)-seq analysis in cells overexpressing PQBP1, showing an enrichment heatmap at promoter regions genome-wide (G) and Kyoto Encyclopedia of Genes and Genomes (KEGG) pathway analysis of target genes (H). (I and J) Quantitative polymerase chain reaction (qPCR) analysis of representative signaling pathway target genes and the metabolic regulatory gene (J) mRNA transcription in cells overexpressing PQBP1. (K) In vitro assay showing the direct effect of PQBP1 on the methyltransferase activity of PRMT5. (L to N) Assessment of epithelial-immune crosstalk via conditioned media (CM) transfer. THP-1 differentiated macrophages were incubated for 24 h with CM collected from BEAS-2B cells (transfected with si-NC or si-*PCK2* and stimulated with HDM). Bar graphs show the relative mRNA expression levels of the proinflammatory cytokines *IL-1β* (L), *IL-6* (M), and *TNF-α* (N) in macrophages. All Western blots are representative of ≥3 independent experiments. Data are presented as means ± standard error of the mean (SEM) (*n* = 3 independent experiments). Analyzed by Student *t* test or 1-way analysis of variance (ANOVA). *P < 0.05, **P < 0.01, ***P < 0.001, ****P < 0.0001; ns, not significant.

To investigate the downstream mechanism of lactylated PQBP1, we identified its significant interaction with the PRMT5/WD repeat domain 77 (WDR77) epigenetic repressive complex through proteomic analysis (Fig. 6A and Fig. S13A and B). This physical interaction was confirmed by endogenous Co-IP and observed in situ via nuclear colocalization (Fig. 6B and Fig. S13C). To further elucidate the molecular mechanism by which PQBP1 “directly” inhibits PRMT5, we mapped their binding interface using molecular docking techniques (Fig. 6C). The structural model revealed that PQBP1 binds to a specific pocket on the surface of PRMT5. Specifically, a positively charged cluster composed of R121, R124, and K125 within the WW domain of PQBP1 forms strong electrostatic interactions and a hydrogen bond network with an electronegative pocket in PRMT5 (containing Y304, Y307, and E320) (Fig. 6C). To validate these predicted binding sites, we constructed site-directed mutants for PRMT5 (Mut A: Y304A/Y307A/E320A; Mut B: D70A as a control) and PQBP1 (Mut 1: R121A/R124A/K125A; Mut 2: K71A/E74A as a control) (Fig. 6D). Co-IP assays demonstrated that the interaction between PRMT5 and PQBP1 was significantly abolished when the core binding residues were mutated (PQBP1-Mut 1 and PRMT5-Mut A). In contrast, mutations in noninterface regions (Mut 2 and Mut B) had a negligible effect on binding affinity (Fig. 6E). Collectively, these data suggest that PQBP1 directly binds to PRMT5 via a specific “charge-clamp” mechanism, and this physical association likely inhibits PRMT5 enzymatic activity through steric hindrance.

To explore the functional consequence of this interaction, we next examined the effect of PQBP1 on the overall catalytic activity of PRMT5 by Western blot. The results showed that overexpression of PQBP1 significantly decreased the global levels of H4R3me2s, a modification catalyzed by PRMT5 (Fig. 6F). This result indicates that PQBP1 can inhibit the methyltransferase activity of PRMT5 within cells. Functionally, PQBP1 acts as an inhibitor of this complex. Overexpression of PQBP1 led to a genome-wide reduction of the PRMT5-catalyzed repressive mark H4R3me2s, with a particularly pronounced decrease in promoter regions (Fig. 6G and Fig. S13D to G). These results reveal a dual regulatory mechanism: Overexpression of PQBP1 not only partially down-regulated the total protein level of PRMT5 (Fig. S13H) but, more critically, it potently inhibited its methyltransferase catalytic activity, thereby driving the significant reduction in downstream H4R3me2s levels.

This epigenetic alteration led to the derepression of 2 key classes of functional genes. Chromatin immunoprecipitation (ChIP)-seq analysis revealed that the derepressed genes were significantly enriched in the proinflammatory mitogen-activated protein kinase (MAPK) signaling pathway (Fig. 6H and I and Figs. S13I and S14A and B) and among key metabolic regulators (Fig. 6J and Fig. S14C and D). The underlying mechanism was determined to be direct enzymatic inhibition, as an in vitro assay demonstrated that PQBP1 inhibited the methyltransferase activity of PRMT5 (Fig. 6K). This finding provides a mechanistic explanation for the previously observed proglycolytic phenotype, linking PQBP1’s epigenetic function to the regulation of metabolic genes.

Finally, global transcriptomic analysis revealed the widespread impact of this regulatory axis. PQBP1 overexpression induced a pervasive transcriptional reprogramming, altering the expression of nearly 3,000 genes (Fig. S15A). This reprogramming involved a correlation with altered metabolism characterized by the suppression of genes related to homeostatic processes like the “Spliceosome”, alongside the activation of genes associated with pathological processes such as the “Cell cycle” (Fig. S15B to G). In summary, these data establish lactylated PQBP1 as a critical mediator that links metabolic signals to epigenetic and transcriptional changes, thereby promoting a proinflammatory and hypermetabolic cellular state.

Crucially, epithelial–macrophage coculture assays demonstrated that conditioned media from HDM-stimulated epithelial cells potently induced macrophage polarization and cytokine release (IL-1β, IL-6, and tumor necrosis factor-α [TNF-α]). To determine the exact molecular basis of this intercellular communication, we analyzed the expression of classical epithelium-derived alarmins. Quantitative PCR (qPCR) validation confirmed that both HDM and direct lactate stimulation robustly up-regulated the transcription of key alarmins, most notably *TSLP*, in BEAS-2B cells. Importantly, this alarmin expression was significantly abrogated by epithelial PCK2 silencing (Fig. S15H). Consistent with this, the macrophage activation in the coculture system was significantly abrogated by epithelial PCK2 silencing (Fig. 6L to N), confirming that the PCK2–lactate axis acts as an upstream driver orchestrating epithelial-immune crosstalk by synergistically providing a direct metabolic signal and triggering the secretion of specific macrophage-polarizing cytokines.

### In vivo validation of PQBP1-K223 lactylation

**Fig. 7.**
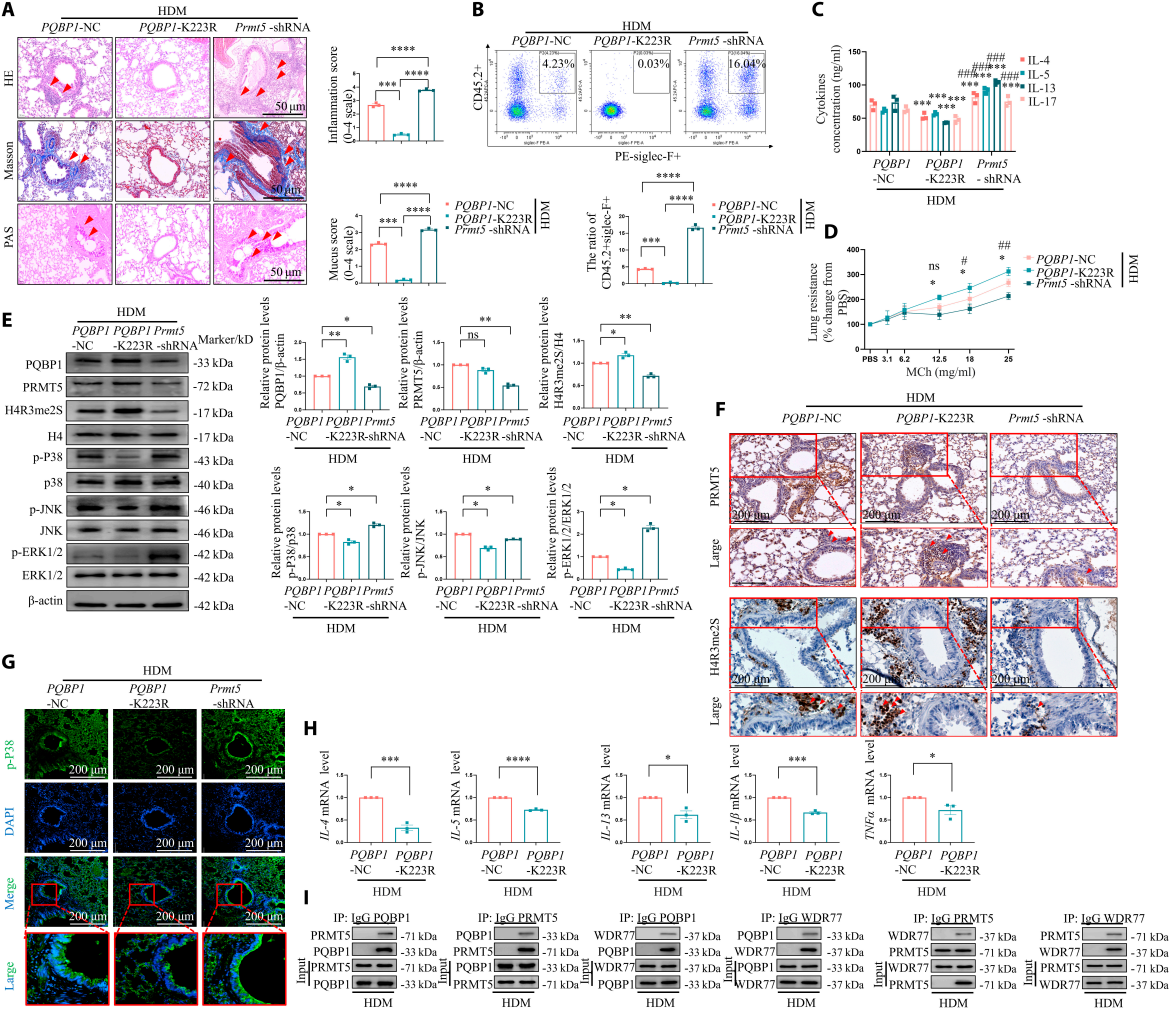
Polyglutamine-binding protein 1 (PQBP1)-K223 lactylation drives airway inflammation in vivo via the protein arginine methyltransferase 5 (PRMT5)/mitogen-activated protein kinase (MAPK) axis. House dust mite (HDM)-challenged mice were treated with adeno-associated viruses (AAVs) delivering *PQBP1*-NC, *PQBP1*-K223R, or *Prmt5*-short hairpin RNA (shRNA), followed by: (A) Pathological staining (hematoxylin and eosin [H&E], Masson, and periodic acid-Schiff [PAS]) of lung tissues. Scale bar, 50 μm. (B) Flow cytometric quantification of eosinophils (CD45.2^+^ CD11c^−^ Siglec-F^+^) in bronchoalveolar lavage fluid (BALF). (C)The concentrations of interleukin-4 (IL-4), IL-5, IL-13, and IL-17 in the BALF supernatant were measured by enzyme-linked immunosorbent assay (ELISA) in HDM-challenged mice transduced with AAV-*PQBP1*-NC, AAV-*PQBP1*-K223R, or AAV-sh*Prmt5*. Data are presented as means ± standard error of the mean (SEM). *P* < 0.001 versus PQBP1-NC group; ###*P* < 0.001 versus PQBP1-NC group (indicating exacerbation by shPrmt5***). (D) Assessment of airway hyperresponsiveness (AHR) regulated by the PQBP1–PRMT5 axis. Lung resistance (% change from phosphate-buffered saline [PBS]) was measured in HDM-challenged mice transduced with AAV-*PQBP1*-NC, AAV-*PQBP1*-K223R, or AAV-sh*Prmt5* in response to increasing concentrations of methacholine (MCh). Data are presented as means ± SEM. Symbols indicate statistical significance compared to the *PQBP1*-NC group (* indicates *PQBP1*-K223R versus NC; # indicates *Prmt5*-shRNA versus NC). (E) Western blot analysis of PRMT5, H4R3me2s, and MAPK pathway phosphorylation in lung tissues. (F and G) Immunohistochemistry (IHC) staining for PRMT5/H4R3me2s (F) and immunofluorescence (IF) staining for p-P38 (G) in lung sections. Scale bar, 200 μm. (H) Quantitative polymerase chain reaction (qPCR) analysis of T helper 2 (Th2) (*Il-4*, *Il-5*, and *Il-13*) and proinflammatory cytokine (*Il-1β* and *Tnf-α*) mRNA in lung tissues. (I) Endogenous coimmunoprecipitation (Co-IP) validating the PQBP1–PRMT5-WD repeat domain 77 (WDR77) interaction in lung tissues. All Western blots are representative of ≥3 independent experiments. Data are presented as means ± SEM (*n* = 6 to 8 per group). Analyzed by Student *t* test, 1 or 2-way analysis of variance (ANOVA). *P < 0.05, **P < 0.01, ***P < 0.001, ****P < 0.0001; ns, not significant.

To validate the pathological function of the PQBP1–PRMT5 signaling axis in vivo, we employed adeno-associated virus (AAV)-mediated gene modulation in the HDM-induced mouse model of asthma (Fig. S16). Consistent with our proposed mechanism, expression of the nonlactylatable PQBP1-K223R mutant significantly attenuated airway inflammation, mucus hypersecretion, and collagen deposition. Conversely, knocking down its downstream target, PRMT5, exacerbated these pathological features (Fig. 7A and B). Mechanistically supporting these histological observations, ELISA analysis of the BALF supernatant revealed that the K223R mutation significantly blunted the HDM-induced cytokine storm, leading to reduced levels of type 2 (IL-4, IL-5, and IL-13) and type 17 (IL-17) cytokines. In sharp contrast, *Prmt5* silencing further elevated these inflammatory mediators, confirming the critical regulatory function of this axis on the immune microenvironment (Fig. 7C). To confirm whether these histological changes translated into physiological functional outcomes, we assessed AHR. As shown in Fig. 7D, the protective effect of the K223R mutation was functionally validated by significantly reduced airway resistance in response to methacholine. In contrast, *Prmt5* knockdown resulted in severe AHR, exhibiting significantly higher lung resistance (LR) compared to the control group.

At the molecular level, these opposing phenotypes correlated with the regulation of the PRMT5-MAPK pathway. Expression of PQBP1-K223R maintained high levels of the repressive mark H4R3me2s and suppressed MAPK pathway activation, whereas *Prmt5* knockdown reduced H4R3me2s levels and increased MAPK phosphorylation (Fig. 7E). These findings were further corroborated in lung tissue sections by immunohistochemistry for PRMT5 and H4R3me2s and by immunofluorescence for p-P38 (Fig. 7F and G). Functionally, the suppression of MAPK signaling in the PQBP1-K223R group was associated with a significant reduction in the transcription of proinflammatory cytokines (Fig. 7H). Furthermore, we confirmed the physical interaction between PQBP1 and the PRMT5/WDR77 complex in asthmatic lung tissues via endogenous Co-IP (Fig. 7I).

Collectively, these in vivo results establish a core mechanism whereby lactylation of PQBP1 at the K223 site contributes to airway inflammation by inhibiting PRMT5-mediated epigenetic silencing, which in turn leads to the activation of the MAPK pathway.

### Therapeutic targeting of the PQBP1 axis

**Fig. 8.**
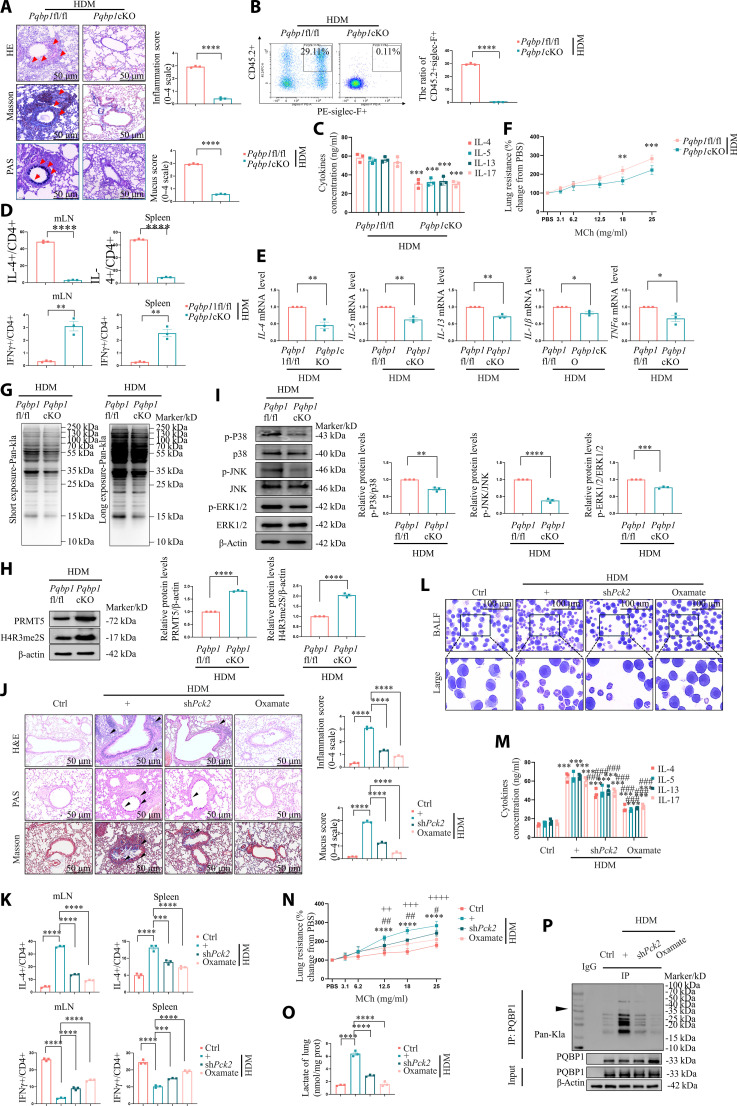
Genetic deletion and therapeutic intervention confirm the central role of the polyglutamine-binding protein 1 (PQBP1) axis. (A to I) Evaluation of house dust mite (HDM)-induced asthma phenotypes in *Pqbp1*fl/fl and airway epithelium-specific *Pqbp1*fl/fl conditional knockout (cKO) mice. (A) Pathological staining (hematoxylin and eosin [H&E], Masson, and periodic acid-Schiff [PAS]) of lung tissues. Scale bar, 50 μm. (B) Flow cytometric quantification of eosinophils (CD45.2+CD11c−Siglec-F+) in bronchoalveolar lavage fluid (BALF). (C) Enzyme-linked immunosorbent assay (ELISA) analysis of inflammatory cytokines (interleukin-4 [IL-4], IL-5, IL-13, and IL-17) in BALF supernatant. (D) Analysis of T helper 1 (Th1)/Th2 cell proportions in lymph nodes and spleen. (E) mRNA expression levels of proinflammatory cytokines. (F) Assessment of airway hyperresponsiveness (AHR). Lung resistance was measured in response to increasing concentrations of methacholine (MCh). (G to I) Western blot analysis of global protein lactylation (Pan-Kla), H4R3me2s levels, and mitogen-activated protein kinase (MAPK) pathway phosphorylation. (J to P) Evaluation of therapeutic efficacy targeting the phosphoenolpyruvate carboxykinase 2 (PCK2)–lactate axis (sh*Pck2* or oxamate) in HDM-challenged mice. (J) Pathological staining (H&E, Masson, and PAS) of lung tissues. Scale bar, 50 μm. (K) Flow cytometric analysis of Th2 and Th1 cells in mediastinal lymph node (mLN) and spleen. (L) Diff-Quik staining of BALF cells. Scale bar, 100 μm. (M) ELISA analysis of inflammatory cytokines (IL-4, IL-5, IL-13, and IL-17) in BALF supernatant. (N) Assessment of AHR following *Pck2* knockdown or lactate inhibition. Lung resistance (% change from phosphate-buffered saline [PBS]) was measured in response to increasing concentrations of MCh. Symbols indicate statistical significance compared to the HDM group (* versus Ctrl; # versus sh*Pck2*; + versus oxamate). (O) Measurement of intrapulmonary lactate concentrations. (P) Immunoprecipitation (IP)-Western blot analysis of PQBP1 lactylation levels. Data are presented as means ± standard error of the mean (SEM). All Western blots are representative of ≥3 independent experiments. Data are presented as means ± SEM (*n* = 6 to 8 per group). Analyzed by Student *t* test, 1 or 2-way analysis of variance (ANOVA). *P < 0.05, **P < 0.01, ***P < 0.001, ****P < 0.0001.

To genetically validate the necessity of epithelial PQBP1 in contributing to airway inflammation, we generated airway epithelium-specific *Pqbp1* conditional knockout (cKO) mice (Fig. S17A to D). In the HDM-induced asthma model, *Pqbp1* cKO mice exhibited a robustly protective phenotype, with substantial reductions in inflammatory infiltration, goblet cell hyperplasia, and collagen deposition (Fig. 8A). This was associated with a marked decrease of eosinophil counts in the BALF to near-baseline values (Fig. 8B). Consistent with the reduced cellular infiltration, ELISA analysis of the BALF supernatant confirmed that the secretion of key inflammatory cytokines (IL-4, IL-5, IL-13, and IL-17) was significantly blunted in *Pqbp1* cKO mice compared to the control group (Fig. 8C). This suppression of the immune microenvironment was further corroborated by flow cytometry and qPCR analyses (Fig. 8D and E and Fig. S17E). To determine whether the observed histological attenuation translates into improved physiological function, we assessed AHR, a hallmark feature of asthma. We measured RL in response to increasing concentrations of methacholine using the FlexiVent system. As shown in Fig. 8F, HDM challenge induced a robust increase in airway resistance in the *Pqbp1*^fl/fl control mice. In contrast, *Pqbp1* cKO mice exhibited significantly reduced airway resistance compared to the control group, indicating that PQBP1 deficiency effectively alleviates HDM-induced AHR. Mechanistically, the deletion of *Pqbp1* reversed the key molecular hallmarks of asthma, leading to decreased global protein lactylation, restoration of H4R3me2s levels, and a blockade of MAPK pathway activation (Fig. 8G to I).

Building on the critical role of PQBP1, we further explored the therapeutic potential of targeting its upstream activating pathway—the PCK2–lactate axis. In vivo administration of shRNA targeting *Pck2* or the lactate metabolism inhibitor oxamate effectively ameliorated lung pathology and the associated Th2 inflammation (Fig. 8J to L and Fig. S17F and G). ELISA analysis further demonstrated the efficacy of these interventions, showing that both sh*Pck2*and oxamate treatment significantly suppressed the release of IL-4, IL-5, IL-13, and IL-17 in the BALF, reversing the HDM-induced cytokine storm (Fig. 8M). Furthermore, we evaluated the physiological efficacy of these interventions. Assessment of AHR revealed that while the vehicle-treated HDM group developed severe airway resistance in response to methacholine, treatment with either sh*Pck2* or oxamate significantly mitigated this response (Fig. 8N). This confirms that blocking the PCK2–lactate metabolic axis effectively restores airway function. Both interventions reduced intrapulmonary lactate accumulation (Fig. 8O) and, critically, inhibited the lactylation of the downstream effector molecule PQBP1 (Fig. 8P).

Taken together, these findings from genetic knockout and therapeutic intervention models provide strong in vivo evidence for the central role of the “PCK2-lactate-PQBP1-PRMT5” signaling axis in association with airway inflammation. This work not only validates epithelial PQBP1 as a promising drug target but also suggests that targeting upstream nodes of this pathway is a viable strategy for asthma treatment.

### HDAC1/2 function as “erasers” for PQBP1 lactylation

**Fig. 9.**
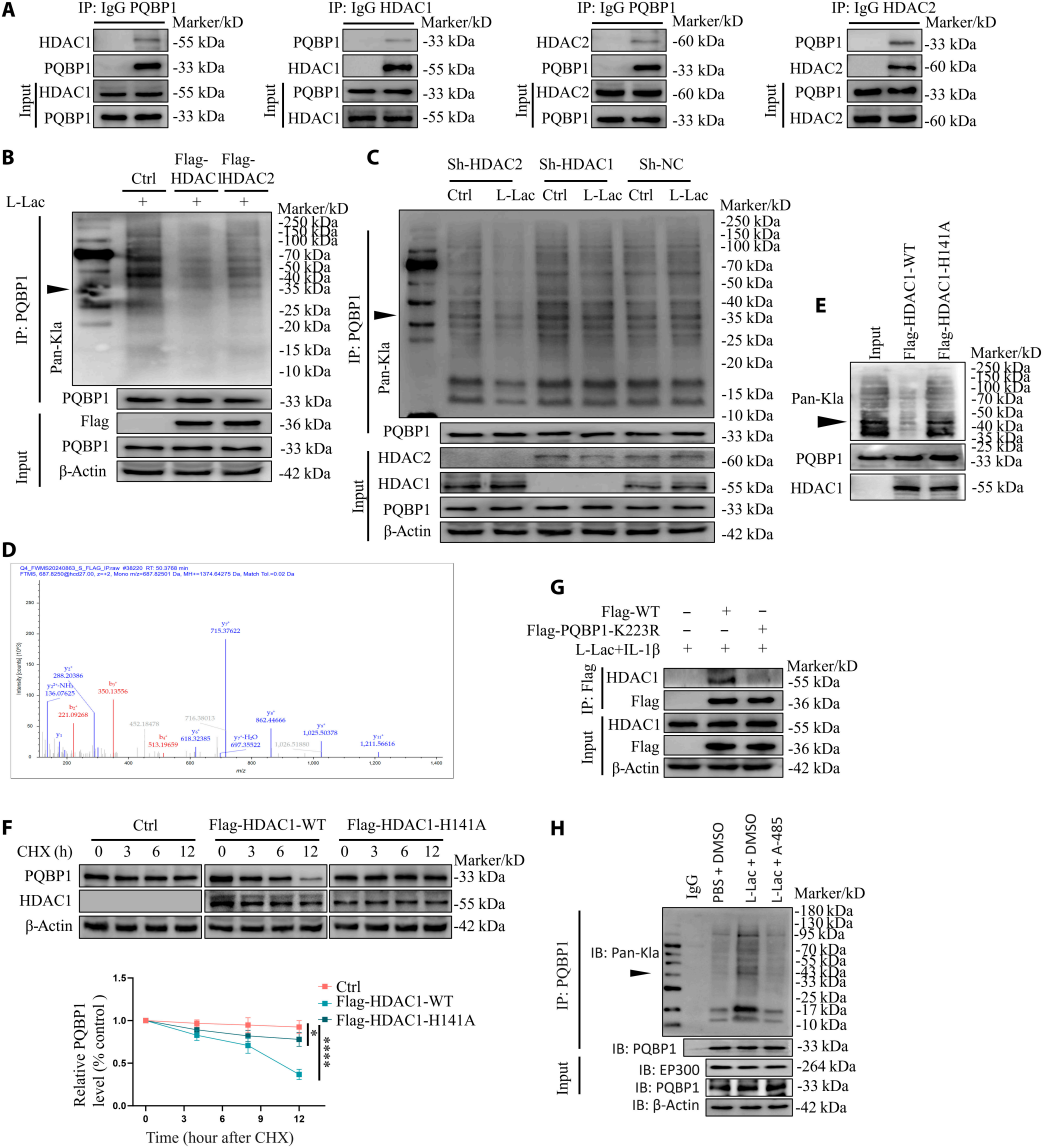
HDAC1 regulates polyglutamine-binding protein 1(PQBP1) lactylation and protein stability. (A) Endogenous coimmunoprecipitation (Co-IP) showing the interaction of PQBP1 with HDAC1/2 in BEAS-2B cells. (B and C) Detection of lactate-induced PQBP1 lactylation following overexpression (B) or knockdown (C) of HDAC1/2. (D) Comparison of the signal abundance of HDAC1 interacting with PQBP1 from immunoprecipitation-mass spectrometry (IP-MS) data. (E) In vitro HDAC1 delactylation assay. Purified hyper-lactylated Flag-PQBP1 substrate was incubated with recombinant HDAC1-WT (wild-type) or the catalytically inactive mutant (H141A), and PQBP1 lactylation levels were assessed by Western blot (Pan-Kla). (F) Cycloheximide (CHX) chase assay to determine the effect of overexpressing HDAC1 (WT or inactive mutant) on the degradation rate of PQBP1. (G) Co-IP experiment to assess the interaction of HDAC1 with WT or K223R mutant PQBP1. (H) Immunoprecipitation (IP) and Western blot analysis of PQBP1 lactylation (Pan-Kla) in BEAS-2B cells. Cells were pretreated with the EP300-specific catalytic inhibitor A-485 (or dimethyl sulfoxide [DMSO] vehicle) for 2 h prior to lactate (L-Lac) stimulation. Total EP300 and PQBP1 in the input, as well as the immunoprecipitated PQBP1, were verified. The black arrowhead indicates the target lactylated PQBP1 band. All Western blots are representative of ≥3 independent experiments. Data are presented as means ± standard error of the mean (SEM) (*n* = 3 independent experiments). Analyzed by 1 or 2-way analysis of variance (ANOVA). *P < 0.05, ****P < 0.0001.

To investigate the dynamic regulation of PQBP1 lactylation, we found that PQBP1 interacts with histone deacetylases HDAC1 and HDAC2 (Fig. 9A), an association that was enhanced upon IL-1β stimulation (Fig. S18A). Experiments demonstrated that while overexpression of either HDAC1 or HDAC2 could inhibit PQBP1 lactylation, the effect of HDAC1 was more pronounced. Furthermore, specific knockdown of HDAC1 led to a significant accumulation of lactylated PQBP1 (Fig. 9B and C and Fig. S18B and C). This functional evidence collectively points to HDAC1 as the key enzyme mediating the delactylation of PQBP1. Consistent with this conclusion, a reexamination of our original protein interactome data revealed that the mass spectrometry signal intensity for HDAC1 was the strongest among all interacting deacylases (Fig. 9D).

To rigorously validate the direct, enzyme-dependent “delactylase” activity of HDAC1, we performed an in vitro enzymatic assay using purified proteins. We incubated physiological, hyperlactylated PQBP1 (purified from lactate-stimulated cells) with either wild-type HDAC1 (HDAC1-WT) or a catalytically inactive mutant (HDAC1-H141A). As shown in Fig. 9E, incubation with HDAC1-WT almost completely abolished the lactylation signal of PQBP1. In sharp contrast, the H141A mutant failed to remove the lactyl group, resulting in signal intensity comparable to the input control. These results provide direct biochemical evidence that HDAC1 functions as a bona fide delactylase for PQBP1, a finding that is consistent with emerging studies identifying Class I HDACs as primary erasers of lysine lactylation [29,30].

Building on the central role of HDAC1, we further explored its regulatory mechanism. We found that HDAC1 promotes the degradation of PQBP1, an effect that is dependent on its enzymatic activity (Fig. 9F). Concurrently, the interaction between PQBP1 and HDAC1 was dependent on the K223 residue (Fig. 9G), suggesting that the lactylation status of PQBP1 may regulate its binding to this demodifying enzyme.

### Multiomics integration validates the PCK2–lactate–PQBP1 axis in clinical asthma

To rigorously validate the clinical relevance of our mechanistic findings, we employed a multiomics triangulation strategy by integrating bulk transcriptomic data from bronchial brushings (GSE43696) with scRNA-seq data from asthmatic airway epithelium (GSE193816). First, we examined the metabolic basis of the disease. In the bulk cohort (GSE43696), the metabolic drivers PCK2 and HK2 were significantly up-regulated (Fig. S19A and C). Complementing this, analysis of the single-cell dataset (GSE193816) revealed a specific up-regulation of the succinate sensor SUCNR1 and the lactate generator lactate dehydrogenase A in asthmatic epithelial cells (Fig. S19B and D). Next, to address the mechanism of protein lactylation, we further analyzed the scRNA-seq dataset (GSE193816). We observed a distinct clustering of asthmatic epithelial cells (Fig. S19E) and, crucially, found that EP300, the primary lactyltransferase responsible for catalyzing lysine lactylation, was significantly up-regulated in the asthmatic group (Fig. S19F). This up-regulation of the “writer” enzyme provides the enzymatic basis for the elevated lactylation events. To provide direct biochemical evidence supporting this clinical observation, we performed in vitro pharmacological inhibition assays. We pretreated BEAS-2B cells with A-485, a highly specific competitive inhibitor of the EP300 catalytic domain, prior to lactate stimulation. Co-IP results demonstrated that inhibiting EP300 catalytic activity significantly abrogated the lactate-induced lactylation of PQBP1, while the total protein pool of PQBP1 remained unaffected (Fig. 9H). This functional loss-of-function evidence firmly establishes EP300 as the bona fide “writer” (lactyltransferase) specifically responsible for PQBP1 lactylation in our model. Most importantly, despite the up-regulation of these metabolic and enzymatic drivers, the mRNA levels of the downstream effectors PQBP1 and PRMT5 remained highly stable across healthy and severe asthma groups (Fig. S19G and H). This clinical observation aligns perfectly with our experimental data (Fig. S4C), reinforcing our conclusion that the pathological function of PQBP1 is driven by EP300-mediated posttranslational lactylation rather than transcriptional abundance. Collectively, these multiomics data provide coherent verification that the core molecular machinery driving the PCK2–lactate–PQBP1–PRMT5 axis is clinically operative in human asthma. Finally, to ascertain the clinical and translational relevance of the identified metabolic–epigenetic axis beyond transcriptional profiling, we utilized primary human bronchial epithelial cells (pHBECs) to simulate varying degrees of asthma severity in vitro. Consistent with our findings in the BEAS-2B cell line, exposure to a concentration gradient of HDM provoked a dose-dependent up-regulation in both PRMT5 expression and H4R3me2s levels (Fig. S20A). More importantly, the specific succinylation of PCK2 and lactylation of PQBP1 were also significantly intensified alongside the escalating HDM burden, despite unchanged total protein levels (Fig. S20B and C). These primary cell-derived data compellingly demonstrate that the PCK2–PQBP1–PRMT5 axis is tightly correlated with asthma severity in a human physiological context, thereby reinforcing its potential as a therapeutic target.

## Discussion

**Fig. 10.**
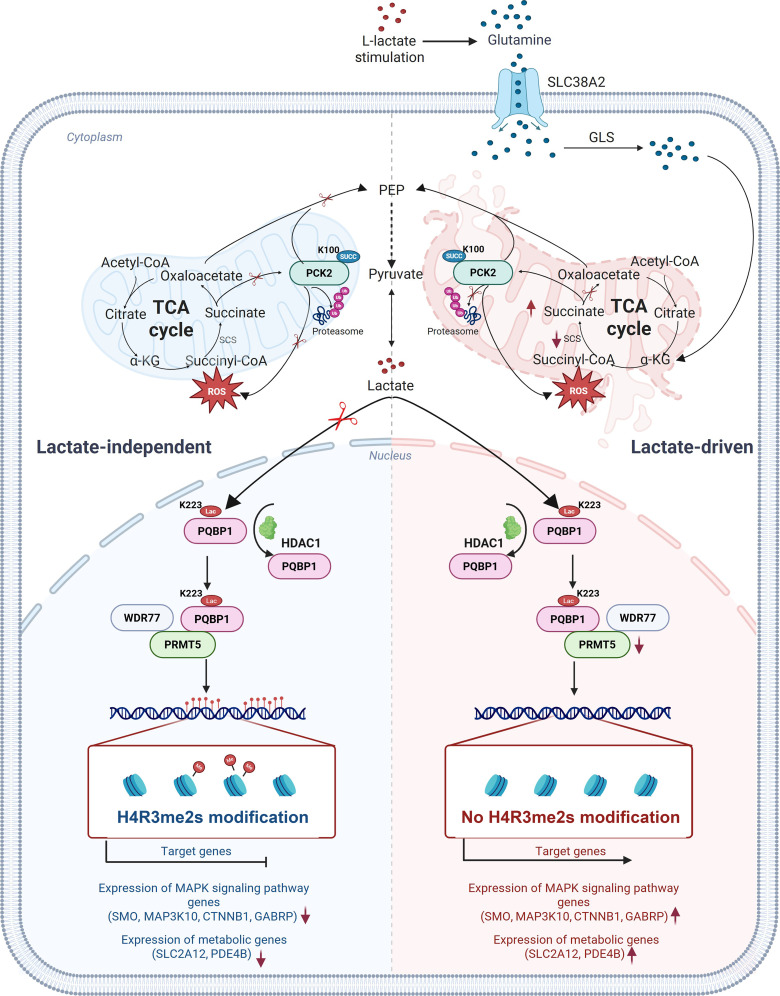
Schematic model of the phosphoenolpyruvate carboxykinase 2 (PCK2)–lactate–polyglutamine-binding protein 1 (PQBP1)–protein arginine methyltransferase 5 (PRMT5) signaling axis driving airway inflammation. The diagram illustrates that in the asthmatic microenvironment (right), lactate accumulation induces the stabilization of PCK2 via succinylation (K100), establishing a lactate positive feedback loop. The amplified lactate signal then induces the lactylation of PQBP1 (K223), which in turn inhibits PRMT5 activity, ultimately lifting the H4R3me2s-dependent transcriptional repression of downstream proinflammatory genes (e.g., the mitogen-activated protein kinase [MAPK] pathway). In the basal state (left), this pathway is suppressed.

This study delineates a complete and novel signaling cascade that functionally links metabolic reprogramming to epigenetic dysregulation, ultimately contributing to chronic airway inflammation (Fig. 10). Specifically, we have systematically elucidated a pathogenic mechanism within the inflammatory microenvironment of allergic asthma, wherein a signal initiated by a core metabolite is amplified via a PTM cascade and ultimately converges on epigenetic regulation. In this pathway, we reveal that lactate is not merely a product of metabolic dysregulation but a core signaling molecule [8]. It amplifies its own signal through a PCK2-mediated positive feedback loop, relays the metabolic stress signal to the effector protein PQBP1, and ultimately, through the modulation of the PRMT5 epigenetic repressive complex, transduces the inflammatory signal into a persistent proinflammatory gene expression program.

Our investigation began with the observation of a synergistic accumulation of lactate and succinate in our asthma model. Mechanistically, we confirmed that lactate drives this succinate accumulation by up-regulating the glutamine transporter SLC38A2 and GLS. By cross-referencing our global lactyl- and succinyl-proteomics datasets, we verified that the up-regulation of SLC38A2 and GLS is primarily driven at the transcriptional level, as their PTMs (including lactylation and succinylation) were either unchanged or significantly down-regulated, ruling out PTM-driven protein stabilization. This led to an intriguing observation of metabolic–epigenetic uncoupling, where elevated succinate levels were unexpectedly accompanied by a general decrease in global protein succinylation. While intracellular succinate accumulation is typically thought to promote succinylation, our mechanistic investigation resolved this paradox. We confirmed that the expression of SIRT5, the primary mitochondrial desuccinylase, remained unchanged, ruling out enhanced enzymatic removal as the cause. Instead, our data identified mitochondrial dysfunction—specifically the inhibition of SCS—as the culprit. This impairment blocks the TCA cycle and depletes succinyl-CoA, the direct donor for the modification, thereby causing a “substrate shortage” that overrides the effect of succinate accumulation. This observation is highly consistent with the energy metabolism crises observed in other pathological stress states [31,32].

Against this backdrop, we captured a key molecular event: the specific succinylation of PCK2 at its K100 site. A compelling aspect of this finding is the specific preference for succinylation over lactylation despite a globally lactate-rich cellular environment. We attribute this to the subcellular compartmentalization of metabolism. PCK2 is strictly localized to the mitochondrial matrix. While the overarching cytosolic and nuclear compartments become enriched with lactate and lactyl-CoA (driving the lactylation of specific nuclear targets), the localized microenvironment surrounding PCK2 within the mitochondria accumulates a massive pool of succinyl-CoA due to heightened glutaminolysis. Thus, spatial isolation and localized substrate availability dictate its preferential succinylation. Crucially, it is imperative to address the apparent paradox of why PCK2-specific succinylation increases despite the global decline in succinyl-CoA and the absence of a known specific “writer”. Extensive studies establish that mitochondrial protein succinylation is predominantly driven by nonenzymatic chemical acylation rather than specific enzymatic writers [33,34]. This nonenzymatic process is highly dependent on the slightly alkaline pH of the mitochondrial matrix and immediate physical proximity to the local generation of highly reactive succinyl-CoA via the α-ketoglutarate dehydrogenase complex. Because PCK2 remains strictly localized within the mitochondrial network even during stress-induced fragmentation, this unique spatial microenvironment permits PCK2 to preferentially and nonenzymatically undergo K100 succinylation, successfully competing against the global substrate shortage. PCK2 is conventionally regarded as a key rate-limiting enzyme in gluconeogenesis [25]. However, a central finding of this study is that PCK2 exhibits context-dependent functional plasticity. Under the metabolic stress and mitochondrial dysfunction characteristic of asthmatic inflammation, succinylation at the K100 site acts as a molecular switch that redirects the enzyme’s function. While PCK2 maintains its cataplerotic activity to generate PEP from TCA cycle intermediates [35], the fate of this PEP is dramatically altered. In the context of impaired mitochondrial function and enhanced glycolytic flux found in inflamed cells [8], this PEP is preferentially shunted toward pyruvate and lactate production rather than glucose synthesis. This functional shift thereby contributes to the significant lactate accumulation observed in the inflammatory microenvironment [6].

This PTM-driven alteration of enzymatic function not only explains the persistent and substantial accumulation of lactate in the asthmatic microenvironment but also unveils a novel paradigm of metabolic regulation: Cells can fundamentally alter the functional attributes of enzymes at key metabolic nodes through PTMs to adapt to the demands of the pathological microenvironment, even forming a self-reinforcing positive feedback loop. The complex crosstalk between PTMs is critical in determining a protein’s functional fate [36]. For example, succinylation promotes protein degradation in septic cardiomyopathy [37] but inhibits it in specific pathways in fatty liver disease [38]. Echoing these findings, our study in asthmatic lung tissue reveals a new mode where succinylation protects PCK2 from degradation, thereby creating a pathogenic positive feedback loop. Specifically, our data illustrate a competitive occupancy at the K100 residue, where targeted succinylation directly antagonizes ubiquitination. Concurrently, our omics datasets confirmed the absence of competing lactylation at this specific site under our experimental conditions, ensuring that succinylation serves as the primary and specific functional driver for PCK2 stabilization.

Notably, the coelevation of Th2 cytokines and IL-17 observed in our model mirrors the heterogeneity of severe human asthma. The up-regulation of IL-17 is of particular interest in the context of our metabolic findings, as lactate accumulation has been shown to preferentially support Th17 differentiation [39], potentially creating a feed-forward loop that exacerbates airway remodeling and hyperresponsiveness.

The functional diversity of PQBP1 has only recently begun to be unveiled, with its traditional roles primarily defined as a regulator of RNA processing and a core protein in neurodevelopment [27,40]. Recent studies have further expanded its functions, identifying it as an intracellular sensor in innate immunity that recognizes viruses and pathological proteins [26,41]. However, our study reveals a completely new function for PQBP1 in the field of immunometabolism. We have identified PQBP1 as a lactate signal sensor by integrating our global lactyl-proteome and proteome datasets, which highlighted PQBP1 as an optimal candidate due to its specifically up-regulated lactylation status against a stable total protein background. We directly linked the increase in lactate—caused by classical inflammatory signals like IL-1β—to the lactylation of PQBP1 at the K223 site. This finding is significant because it clearly delineates at the molecular level how a cell translates a macroscopic inflammatory stimulus into a microscopic and highly specific PTM. PQBP1 thus emerges as a critical hub for signal integration and transduction, representing a new class of signal “decoders” specifically responsible for accurately “translating” a broad, global metabolic stress signal (high lactate) into a downstream gene expression program.

Our findings suggest a specific molecular mechanism. Our ChIP-seq data revealed that PQBP1-mediated PRMT5 inhibition primarily affects H4R3me2s levels at the promoter regions of both proinflammatory and metabolic genes, thereby “unlocking” these key genes. This epigenetic modification “unlocks” these key genes, effectively coupling the inflammatory response to the metabolic processes that sustain it at the epigenetic level.

A pivotal finding of this study is the elucidation of how lactylated PQBP1 regulates downstream gene expression, which provides a novel, noncanonical regulatory model for the core epigenetic repressor PRMT5. The canonical mode of action for PRMT5 is to act as a structural platform for the assembly of a large repressive complex containing various effector enzymes [42,43]. This complex is then precisely recruited to specific genomic loci by transcription factors such as c-Myc, Slug, and LYAR to establish a multilayered repressive chromatin state, thereby inhibiting gene expression [22,44,45]. However, our study reveals a distinct regulatory mechanism: Lactylated PQBP1 directly interacts with the PRMT5/WDR77 complex and inhibits its catalytic activity. This direct functional inhibition of a core epigenetic repressive complex, driven by PQBP1 lactylation, stands in sharp contrast to the established modes of PRMT5 regulation, such as its activation by upstream signaling proteins [46] or the modulation of its expression [47]. Mechanistically, our structural analysis and mutational data provide the first atomic-level insights into the PQBP1–PRMT5 complex. We identified that the WW domain of PQBP1 acts as a molecular “plug”, inserting its positively charged loop (R121/R124/K125) into the functional pocket of PRMT5. This direct physical occupancy supports a steric hindrance model, wherein PQBP1 binding may either lock PRMT5 in an inactive conformation or directly block substrate access to the active site. This finding not only explains the inhibitory effects observed in vitro but also provides a structural basis for the development of peptidomimetic inhibitors targeting this specific interface. Given that PRMT5 has been reported to exert its diverse repressive functions by inhibiting specific microRNA pathways or down-regulating an important regulatory node of proinflammatory pyroptosis like Caspase-1 [48], our findings suggest that in the inflammatory cells of asthma, PQBP1 lactylation may systematically lift the key epigenetic repression on multiple proinflammatory and pro-proliferative pathways by inhibiting PRMT5, thereby contributing to disease exacerbation.

Despite these promising findings, our study has several limitations. First, although the HDM-induced mouse model is a classic tool for studying asthma, it cannot fully replicate the heterogeneity and complexity of human asthma, particularly the chronic airway remodeling observed in patients with long-term disease. Second, while we identified the “lactate-driven glutaminolysis/succinate” cascade as a key driver, metabolic reprogramming in asthma is highly complex. We cannot rule out the possibility that other metabolic pathways or metabolites also contribute to the observed lactylation phenotypes. Third, our initial mechanistic insights were primarily derived from the BEAS-2B cell line and murine models. While we corroborated these findings using public clinical datasets, we did not validate them in fresh clinical tissue samples from asthma patients. While acquiring fresh clinical biopsy tissues in the milligram quantities required for specific PTM immunoprecipitation remains technically and ethically prohibitive, we have effectively mitigated this limitation by utilizing pHBECs. By simulating a clinical severity gradient with HDM in these primary cells, we successfully corroborated the dose-dependent up-regulation of PCK2 succinylation, PQBP1 lactylation, and the downstream PRMT5/H4R3me2s axis within a physiological human genetic context. Although this approach robustly validates our findings beyond immortalized cell lines and public transcriptomic datasets, the ultimate lack of direct biochemical validation in fresh, large-cohort patient biopsies still warrants acknowledgment. Fourth, while our site-directed mutagenesis and mass spectrometry data strongly support K100 and K223 as the primary regulatory sites for PCK2 and PQBP1, respectively, the current reliance on pan-PTM antibodies remains a technical limitation. The future development of site-specific antibodies (e.g., anti-PCK2-K100su and anti-PQBP1-K223la) will be highly valuable for precisely mapping these specific modifications in clinical biopsies. Fifth, while our functional and biochemical assays strongly support the cataplerotic and lactate-generating shift of PCK2, the current study lacks 13C-isotope metabolic flux analysis. This limits our ability to mathematically quantify the precise kinetic proportion of glutamine-derived carbons entering the lactate pool. Future 13C-flux studies are essential to definitively track this dynamic carbon flow and further substantiate the proposed functional “moonlighting” shift of PCK2. Future studies involving primary human samples and these targeted tools are necessary to confirm the translational relevance of our results.

While this study delineates a foundational signaling axis, it also opens several compelling avenues for future investigation. Our work, centered on airway epithelial cells using cKO models, underscores the need to explore the contribution of this metabolic–epigenetic pathway in other key cell types within the complex asthmatic microenvironment, particularly immune cells. Indeed, our coculture models provide direct evidence of a critical epithelial–macrophage crosstalk orchestrated by the PCK2–lactate axis. Our findings support a synergistic dual-driver mechanism for this communication: On one hand, the substantial accumulation of extracellular lactate acts as a direct immunomodulatory signal; on the other hand, the downstream PQBP1–PRMT5 epigenetic derepression directly triggers the robust secretion of classical epithelium-derived alarmins, such as *TSLP* and *IL-33*. This coordinated release of metabolites and cytokines establishes a paracrine signaling network that critically drives macrophage polarization and perpetuates chronic airway inflammation. Interestingly, beyond metabolic enzymes, our lactyl-proteomics analysis revealed a significant enrichment of the spliceosome pathway. We identified novel lactylation sites on key splicing factors, including SF3B2 (a component of U2 small nuclear ribonucleoprotein) and HNRNPU (a heterogeneous nuclear ribonucleoprotein). SF3B2 is crucial for branch point sequence recognition during early spliceosome assembly [49], while HNRNPU is a recognized regulator of exon skipping and retention. Crucially, a reanalysis of our unbiased interactomics (immunoprecipitation-mass spectrometry [IP-MS]) dataset revealed that PQBP1 physically interacts not only with the PRMT5/WDR77 complex but also with several of these core splicing factors, including SF3B2 and HNRNPU. This finding bridges the apparent gap between PQBP1’s epigenetic function and the global spliceosome enrichment, positioning PQBP1 as a dual-function hub that potentially coordinates both PRMT5-mediated transcriptional regulation and downstream alternative splicing. Recent evidence suggests that metabolic signals can fine-tune immune responses by modulating RNA splicing [50]. Therefore, it is highly probable that the lactate-rich microenvironment in asthmatic lungs alters the function of these splicing factors and PQBP1 via lactylation, potentially leading to pathogenic isoform switching of key cytokine receptors (e.g., *Il4ra* and *Crlf2*), thereby exacerbating type 2 inflammation. While identifying specific alternative splicing events driven by lactylation is beyond the scope of this study, our findings provide preliminary evidence for a potential “metabolism-splicing” axis in asthma, which warrants comprehensive exploration in future research. Furthermore, understanding the dynamic regulation of this pathway is crucial. While we identified HDAC1/2 as PQBP1 delactylases, future studies should address how their activity is modulated during the initiation and resolution of asthmatic inflammation.

Our findings have several translational implications for asthma management. The PCK2–lactate–PQBP1–PRMT5 axis components could serve as biomarkers for disease stratification and therapeutic response monitoring. The demonstrated reversibility of this pathway suggests that metabolic interventions could complement existing therapies, particularly in treatment-resistant cases. Potential therapeutic strategies include developing small-molecule inhibitors targeting PCK2-K100 succinylation, PQBP1–PRMT5 interaction inhibitors, or HDAC1 activators to enhance endogenous pathway regulation. The efficacy of oxamate in our models suggests that repurposing existing metabolic drugs could accelerate clinical translation. Based on our mechanistic model, we propose several conceptual therapeutic strategies that warrant future investigation: (a) targeting the enzymatic activity of PCK2 or its specific succinylation at K100; (b) blocking the lactylation of PQBP1 at K223; (c) developing small-molecule inhibitors to disrupt the pathological PQBP1–PRMT5 interaction; or (d) activating HDAC1/2 to enhance the endogenous delactylation of PQBP1. However, targeted delivery to the airway epithelium and careful patient selection will be critical for successful therapeutic development.

In summary, this study systematically elucidates how metabolic dysregulation associates with and perpetuates chronic inflammation in asthma by delineating a signaling cascade that bridges metabolites, PTMs, and epigenetics. We have identified the PCK2-mediated lactate positive feedback loop and the functional inhibition of PRMT5 via PQBP1 as the 2 core mechanisms in this process. Crucially, our final in vivo intervention experiments provide compelling evidence for the clinical translational potential of this pathway. Rather than a simple linear axis, our findings suggest a complex regulatory network where metabolic stress is integrated into the epigenetic landscape. This work provides a novel molecular paradigm for understanding the pathophysiology of asthma and offers a theoretical framework for developing next-generation metabolic–epigenetic therapies for airway inflammation.

## Materials and Methods

### Experimental animals and models

#### Animals and ethics approval

Female BALB/c mice (6 to 8 weeks old, 20- to 22-g body weight) were used in this study and were purchased from the Experimental Animal Center of Yanbian University. For cKO experiments, *Scgb1a1*-iCre mice (Product No. 1001145; Strain name: C57BL/6JCya-Scgb1a1em1(P2A-iCre)/Cya) were purchased from Cyagen Biosciences and were bred on a C57BL/6J genetic background [51]. This model achieves specific and persistent expression of Cre recombinase in bronchial and alveolar epithelial cells by integrating a P2A-iCre element at the stop codon of the endogenous *Scgb1a1* gene.

All mice were housed in specific pathogen-free animal facilities under strictly controlled conditions: temperature of 22 ± 2 °C, relative humidity of 50 ± 10%, and a 12-h light/dark cycle. Mice had ad libitum access to standard chow and sterile water. All animals were acclimated to the environment for at least 1 week before the experiments. All animal experimental protocols were reviewed and approved by the Ethics Committee of the Medical College of Yanbian University (Ethics Approval No.: YD20230710003; Approval Date: 2023 July 10) and were performed in strict accordance with the relevant guidelines for animal welfare and ethics.

#### Preparation of viral vectors for gene intervention

AAV9 viral vectors for in vivo gene knockdown (AAV9-U6-shRNA series, including shRNAs targeting *Pck2* and *Prmt5*) and gene overexpression (AAV9-CMV series, e.g., AAV9-CMV-*PQBP1*-WT, AAV9-CMV-*PQBP1*-K223R), as well as the corresponding negative control viruses (AAV9-shControl, AAV9-CMV-NC), were constructed, packaged, and purified by GeneChem Co., Ltd. (Shanghai, China). The knockdown or overexpression efficiency of all viruses was validated to ensure the intended effect in target cells (e.g., knockdown efficiency ≥70%).

#### Establishment and intervention of an AAV-based asthma model

Mice were randomly allocated into experimental groups (*n* = 6 to 8 per group) using a random number generator and treated as follows:

##### Virus delivery

Seven days before the first sensitization, mice in the gene intervention groups received a slow intranasal instillation of 50 μl of the corresponding AAV9 virus solution (titer: 1.5 × 10^12^ v.g./ml) under isoflurane inhalation anesthesia. The control, HDM asthma model, and some pharmacological intervention groups received an equal dose and volume of negative control viral particles at the same time point.

##### Sensitization and challenge

Sensitization was performed on days 0 and 7 of the experiment. Except for the control group (intraperitoneal injection of 100 μl of phosphate-buffered saline (PBS) containing 2 mg of aluminum hydroxide adjuvant), all other groups of mice were intraperitoneally injected with 100 μl of a suspension containing 50 μg of HDM (Cat# XPB46D3A4, Greer Laboratories, Lenoir, NC, USA) and 2 mg of aluminum hydroxide adjuvant (alum, vac-alu-250, Invivogen). From day 14 to day 35, except for the control group (which received 50 μl of PBS intranasally 3 times a week), all other groups of mice received an intranasal challenge 3 times a week with 50 μg of HDM (dissolved in 50 μl of sterile PBS) under light isoflurane anesthesia.

##### Pharmacological/metabolite intervention

Starting 2 h before the first HDM challenge, the corresponding intervention groups received daily administration of the following: The HDM+BPTES group received intraperitoneal injections of the specific GLS inhibitor BPTES (10 mg/kg/d, SML1865, Sigma-Aldrich, USA); the HDM+Oxamate group received intraperitoneal injections of oxamate (0.75 g/kg/d, Selleck, USA); the Asthma+Lactate group received intraperitoneal injections of lactic acid (0.5 g/kg body weight, pH 6.8, Sigma-Aldrich); and the Asthma+Succinate group received intraperitoneal injections of succinic acid solution (0.5 g/kg body weight, Sigma, W239607, prepared as a 5% w/v solution) [52].

#### Airway epithelium-specific PQBP1 cKO asthma model

##### Animal model

*Pqbp1*^(flox/flox) (*Pqbp1*fl/fl) mice, purchased from Cyagen Biosciences, were crossed with *Scgb1a1*-iCre mice to generate *Pqbp1*fl/fl; *Scgb1a1*-iCre (+) mice, which served as the cKO group. Their Cre-negative littermates (*Pqbp1*fl/fl) were used as the control group.

##### HDM induction

The HDM sensitization and challenge protocol was identical to that used in the AAV experiments. As the Scgb1a1-iCre model mediates constitutive rather than inducible knockout, tamoxifen treatment was not required.

##### Measurement of AHR

AHR was assessed by measuring changes in RL in response to increasing doses of aerosolized methacholine using the FlexiVent system (SCIREQ, Montreal, Canada). Briefly, 24 h after the final HDM challenge, mice were anesthetized with pentobarbital sodium (50 mg/kg, intraperitoneally). A tracheostomy was performed using a specific cannula, and the mice were connected to a computer-controlled small animal ventilator (respiratory frequency: 150 breaths/min; tidal volume: 10 ml/kg). Muscle paralysis was induced to prevent spontaneous breathing artifacts. Following baseline measurement with PBS, the mice were challenged with increasing concentrations of aerosolized methacholine (3.1, 6.2, 12.5, 18, and 25 mg/ml). The peak RL at each concentration was recorded and analyzed using the FlexiWare software.

##### Sample collection

Twenty-four hours after the final HDM challenge (and corresponding intervention), mice were euthanized by cervical dislocation following cardiac puncture under sodium pentobarbital anesthesia. BALF, serum (obtained by centrifugation), and lung tissues were immediately collected. A portion of the lung tissue was immediately snap-frozen in liquid nitrogen for molecular biology analyses, while another portion was fixed in 4% paraformaldehyde (PFA) for pathological analysis.

### Cell culture and treatment

Human bronchial epithelial cells (BEAS-2B; RRID: CVCL_0168) and human embryonic kidney 293T cells (HEK293T), and human acute monocytic leukemia cells (THP-1; Cat#. CL-0233) were purchased from the Cell Resource Center of the Shanghai Institutes for Biological Sciences, Chinese Academy of Sciences (Shanghai, China), and Fuheng Biology (Shanghai, China), and Procell Life Science & Technology Co., Ltd. (Wuhan, China), respectively. All cell lines were confirmed to be mycoplasma-free. Cells were cultured in Dulbecco’s modified Eagle medium supplemented with 10% fetal bovine serum (BaiDi Biotechnology Co., Ltd. [BDBIO]), 100 μg/ml streptomycin, and 100 U/ml penicillin. The cells were maintained in a humidified incubator at 37 °C with 5% CO₂. pHBECs were obtained from ScienCell Research Laboratories and cultured in specific bronchial epithelial cell medium supplemented with corresponding growth factors according to the manufacturer’s instructions. To strictly preserve their primary physiological characteristics, pHBECs were utilized solely between passages 2 and 3 for all experiments.

### Conditioned medium collection and epithelial–macrophage coculture

To investigate the intercellular crosstalk between airway epithelial cells and macrophages, a conditioned medium (CM) transfer model was established. Briefly, BEAS-2B cells were seeded into 6-well plates at a density of 1 × 10^5^ cells/well. Upon reaching 60% to 70% confluence, cells were transfected with *PCK2*-specific small interfering RNA (si-*PCK2*) or negative control siRNA (si-NC) using Lipofectamine 3000 reagent, following the manufacturer’s instructions. Twenty-four hours posttransfection, the medium was replaced, and cells were stimulated with HDM extract (50 μg/ml) or vehicle (PBS) for an additional 24 h. The culture supernatants were then collected and centrifuged at 1,000 × *g* for 10 min at 4 °C to remove cellular debris. The resulting supernatant was defined as epithelial CM and stored at −80 °C or used immediately. For the macrophage assay, THP-1 monocytes were seeded in 24-well plates and differentiated into adherent macrophage-like cells by incubation with 100 nM phorbol 12-myristate 13-acetate for 24 h. After differentiation, cells were gently washed 3 times with sterile PBS to remove residual phorbol 12-myristate 13-acetate and allowed to rest in fresh complete medium for 24 h. Subsequently, the macrophages were incubated with a 1:1 mixture of epithelial CM and fresh medium for 24 h. Finally, total RNA was extracted using TRIzol reagent and reverse transcribed into cDNA. The mRNA expression levels of inflammatory markers, including *IL-1β*, *IL-6*, and *TNF-α*, were quantified via RT-qPCR using SYBR Green Master Mix, with *GAPDH* serving as the internal normalization control.

### RNA extraction and RT-qPCR of airway epithelial cells

To further evaluate the expression of epithelium-derived alarmins in response to metabolic signals, BEAS-2B cells were independently subjected to the indicated treatments (PBS control, 50 μg/ml HDM, HDM+si-*PCK2*, or 10 mM sodium lactate) for 24 h. Total RNA was extracted from these cultured epithelial cells using TRIzol reagent (Invitrogen, Carlsbad, CA, USA) and reverse-transcribed into cDNA using the PrimeScript RT Reagent Kit (Takara, Japan). The mRNA expression levels of *TSLP* and *IL-33* were quantified via RT-qPCR using SYBR Green Master Mix, with *GAPDH* serving as the internal normalization control. All independent experiments were performed in triplicate.

### Reagents and cell treatments

Depending on the experimental design, cells were treated with the following reagents: sodium lactate (10 mM, pH 6.8 to 7.0; Sigma-Aldrich, St. Louis, MO, USA), a pathophysiologically relevant concentration well within the range observed in the local microenvironment of inflamed asthmatic airways [39,53], for 24 h; sodium succinate (10 mM; Cat# W239607, Sigma-Aldrich) for 24 h; lipopolysaccharide (5 μg/ml; Cat# L8274, Sigma-Aldrich) for 24 h; recombinant human IL-1β (10 ng/ml; Cat# 200-01B, PeproTech, Cranbury, NJ, USA) for 24 h; the proteasome inhibitor MG132 (100 μM; Cat# M8699, Sigma-Aldrich) for 6 h; or CHX (100 μM; Cat# 18079, Sigma-Aldrich) for a time course of 0 to 12 h. For antioxidant experiments, cells were pretreated for 2 h with either N-acetylcysteine (5 mM; Cat# A7250, Sigma-Aldrich) or Mito-TEMPO (10 μM; Cat# SML0737, Sigma-Aldrich) before subsequent stimulation. To dynamically simulate the clinical severity gradient of asthma in vitro, pHBECs were treated with varying concentrations of HDM. Specifically, cells were exposed to either a vehicle control (PBS), low-dose HDM (10 μg/ml, simulating mild to moderate asthma), or high-dose HDM (50 μg/ml, simulating severe asthma) for 24 h. For EP300 pharmacological inhibition experiments, cells were pretreated with the highly specific EP300 catalytic inhibitor A-485 (2 μM; Cat# SML2192, Sigma-Aldrich) for 2 h prior to the subsequent stimulation.

### Plasmids, recombinant proteins, and gene transfection

#### Plasmids and key reagents

All expression plasmids used in this study were synthesized, constructed, and sequence-verified by HonorGene Co., Ltd. (Changsha, China).

##### Overexpression plasmids

pcDNA3.1-*PQBP1*-Flag (WT) and its mutants (K189R, K223R, K223Q); pcDNA3.1-*PCK2*-Flag (WT) and its mutant (K100R); pcDNA3.1-Flag-HDAC1 and pcDNA3.1-Flag-HDAC2; pcDNA3.1-*SLC38A2*; pcDNA3.1-*GLS*. The corresponding empty vector (pcDNA3.1-Flag) was used as a negative control.

##### Gene knockdown plasmids

shRNA plasmids targeting PCK2 (sh-*PCK2*), HDAC1 (sh-*HDAC1*), HDAC2 (sh-*HDAC2*), SLC38A2 (sh-*SLC38A2*), and GLS (sh-*GLS*). A negative control shRNA plasmid (sh-NC) was used.

#### Site-directed mutagenesis and plasmid construction

Construction of the expression plasmids for PRMT5 and PQBP1 was performed as previously described. To validate the interaction interface, mutant plasmids (PRMT5-mut A/B and PQBP1-mut 1/2) were generated using a site-directed mutagenesis kit (Vazyme, Nanjing, China) according to the manufacturer’s instructions. The primer sequences used for mutagenesis are listed in Table S1. All constructs were verified by Sanger sequencing to ensure that no unintended mutations were introduced.

Recombinant human PQBP1 protein (Cat# ab126687) was purchased from Abcam. This protein is a full-length human protein with an N-terminal His-tag, produced in an *Escherichia coli* expression system and purified using conventional chromatography techniques. Its purity is >90%, with a predicted molecular weight of 33 kDa.

#### Cell transfection

All transient transfections of plasmids were performed in BEAS-2B cells. Specifically, when cells reached 70% to 80% confluency, the respective plasmids were transfected into the cells using Lipofectamine 3000 Transfection Reagent (#L3000015; Thermo Scientific, Sunnyvale, CA, USA) according to the manufacturer’s instructions. Following transfection, cells were cultured for an additional 24 to 48 h. Crucially, the transfection efficiency was strictly verified by Western blotting to confirm the successful knockdown or overexpression of the target proteins before the cells were subjected to subsequent experimental treatments, such as L-lactic acid stimulation.

### Biochemical and metabolic analyses

#### Enzyme activity assays

##### GLS activity

Assayed according to the instructions of a commercial kit from Solarbio (#BC1450). Enzyme activity was determined by measuring absorbance at 630 nm, and the final data are presented as U·mg^−1^ protein.

##### Hexokinase (HK) and phosphofructokinase activity

Measured using kits from Solarbio (#BC0745 and #BC0535, respectively). The catalytic activity was determined by monitoring the rate of change of nicotinamide adenine dinucleotide phosphate at 340 nm.

##### PEPCK activity

Measured according to the protocol of the Phosphoenolpyruvate Carboxylase Assay Kit (A130-1-1, Jiancheng Bioengineering Institute), with activity normalized to protein concentration.

##### SCS activity

The activity of SCS in the supernatant of BEAS-2B cell lysates was measured using a commercial colorimetric assay kit (Cat# E-BC-K906-M, Elabscience). The experiment was performed in strict accordance with the manufacturer’s instructions. The final enzyme activity was normalized to the total protein concentration and expressed as units per milligram of protein.

#### In vitro PRMT5 methyltransferase activity

PRMT5 activity was measured using the PRMT5 Chemiluminescent Assay Kit (BPS Bioscience, Cat# 52002L), following the manufacturer’s protocol. The kit provides a 96-well plate precoated with a histone H4 peptide substrate. Recombinant human PRMT5/MEP50 (Cat# 51045) was freshly diluted in 1× PRMT5 assay buffer to 5 to 10 ng/μl, and the reaction was initiated by adding 20 μl of enzyme solution (100 ng per well) to the designated wells; 20 μl of 1× PRMT5 buffer was added to “Blank” wells. The plate was incubated at room temperature for 2 h. Wells were washed 3 times with tris-buffered saline with Tween 20, blocked with blocking buffer, and then incubated sequentially with Primary Antibody 4-3 (1:100 dilution) and horseradish peroxidase (HRP)-labeled Secondary Antibody 2 (1:1,000 dilution). Immediately before detection, HRP chemiluminescent substrates A and B were mixed 1:1 and 100 μl was added per well. Chemiluminescence was read in luminescence mode, and blank-subtracted relative light units values were used for quantification. PQBP1 modulation: Purified PQBP1-WT protein (or control buffer) was included in the reaction mixture as indicated.

##### In vitro HDAC1 delactylation assay

To prepare the hyper-lactylated substrate, HEK293T cells were transfected with Flag-PQBP1-WT plasmids. Twenty-four hours posttransfection, cells were treated with 10 mM sodium lactate for an additional 24 h to maximize intracellular lactylation. Cells were lysed in mild lysis buffer free of EDTA, and Flag-PQBP1 was immunoprecipitated using Anti-FLAG M2 magnetic beads (Sigma) and eluted with 3× Flag peptide. Similarly, the enzymes Flag-HDAC1-WT and its catalytically inactive mutant, Flag-HDAC1-H141A, were overexpressed in HEK293T cells and purified using the same protocol. For the in vitro enzymatic assay, the purified Lactyl-PQBP1 substrate was incubated with an excess of HDAC1 enzyme (WT or H141A) in a reaction buffer containing 50 mM tris-HCl (pH 8.0), 137 mM NaCl, 2.7 mM KCl, 1 mM MgCl_2_, 1 mg/ml bovine serum albumin, and 10 μM ZnCl_2_. The reaction was performed at 37 °C for 2 h and terminated by the addition of sodium dodecyl sulfate (SDS) loading buffer followed by boiling for 10 min. Finally, the lactylation status of PQBP1 was analyzed by Western blotting using an anti-Pan-Kla antibody, while anti-Flag and anti-HDAC1 antibodies were used to confirm equal loading of the substrate and enzymes, respectively.

### Metabolite and oxidative stress marker analysis

Total intracellular ROS were quantified using a 2′,7′-dichlorofluorescin diacetate probe (10 μM; #S0033S, Beyotime). To specifically measure mtROS, cells were stained with the MitoSOX Red probe (5 μM; #M36008, Thermo Fisher Scientific). Fluorescence images were acquired using a Cytation 5 Imaging Reader (BioTek), and signal intensity was analyzed with ImageJ software (National Institutes of Health).

Intracellular succinate levels were measured using the Amplex Red Succinic Acid/Succinate Assay Kit (#S0529, Beyotime). Briefly, cell lysates were incubated with the Amplex Red working solution for 30 min at 37 °C, protected from light. The resulting fluorescence was measured on a microplate reader with excitation at 560 nm and emission at 590 nm.

Intracellular lactate content was determined using a colorimetric L-Lactate Assay Kit (Micro method; #BC2235, Solarbio). Following sample processing with the provided extraction solutions, the supernatant was incubated with the kit’s working solution for 10 min at 37 °C in complete darkness. The absorbance at 570 nm was then measured using a microplate reader.

#### Glutamate assay

Intracellular glutamate levels were determined using the Glutamate Assay Kit (Colorimetric) (ab83389, Abcam). Briefly, cells (10^6^) were harvested and homogenized in ice-cold Assay Buffer 31, followed by centrifugation at 10,000 × g for 2 to 5 min at 4 °C to remove insoluble precipitates. The supernatant was collected, and deproteinization was performed if samples contained proteins that might interfere with enzyme activity. Subsequently, samples were added to a 96-well plate, and the volume was adjusted to 50 μl. A 100-μl volume of the prepared Reaction Mix (containing Glutamate Enzyme Mix VIII, Developer Solution III, and Assay Buffer 31) was added to each well. For samples requiring background correction, parallel wells were set up with Background Reaction Mix lacking the enzyme mixture. After mixing, the plate was incubated at 37 °C for 30 min in the dark. The optical density (OD) was measured at 450 nm using a microplate reader. Glutamate content was calculated by subtracting the blank and background values, applying the standard curve (0 to 10 nmol/well), and normalizing to the sample dilution factor and protein concentration.

#### Glutamine assay

Intracellular glutamine levels were measured using the Glutamine Assay Kit (Colorimetric) (ab197011, Abcam). Briefly, samples were homogenized in the provided Assay Buffer on ice and centrifuged at 10,000 × g for 10 min to remove insoluble material. A glutamine standard curve (0 to 10 nmol/well) was prepared according to the manufacturer’s instructions. In a 96-well plate, the processed samples were mixed with the Hydrolysis Enzyme Mix and incubated at 37 °C for 30 min to hydrolyze glutamine into glutamate. Subsequently, the Development Reaction Mix was added, and the plate was incubated at 37 °C for 60 min in the dark to generate the colorimetric signal. The OD was measured at 450 nm. Glutamine concentration was calculated by subtracting the background blank, applying the standard curve, and normalizing to the protein concentration.

#### Oxidative stress markers

The levels of key oxidative stress markers were quantified using commercially available colorimetric assay kits from the Nanjing Jiancheng Bioengineering Institute, including MDA (#A003-1-2), CAT) (#A007-1-1), and SOD (#A001-3-2). The concentration of reduced GSH was measured using a GSH and GSSG Assay Kit (#S0053, Beyotime), which is based on the 5,5′-dithiobis(2-nitrobenzoic acid) colorimetric method.

### Metabolomics and proteomics analyses

#### Untargeted metabolomics

Performed by Bioprofile Technology Co., Ltd (Shanghai, China). Samples were flash-frozen in liquid nitrogen, followed by solvent extraction, sonication, and centrifugation. Analysis was conducted using a liquid chromatography-tandem mass spectrometry system (Thermo Fisher UltiMate 3000 coupled to an Exploris 480 Orbitrap). Differential metabolites were identified using methods such as principal component analysis and orthogonal partial least squares discriminant analysis, followed by pathway enrichment analysis.

#### Proteomics and PTM-omics

Samples were subjected to trypsin digestion and enrichment of modified peptides. Analysis was performed using an Easy-nLC 1000 UHPLC system coupled to a timsTOF Pro mass spectrometer operating in data-independent acquisition parallel accumulation serial fragmentation mode. Data were searched and quantified against a *Homo sapiens* database using Spectronaut (v.17.0). Differential expression analysis was performed using the limma package in R, which applies an empirical Bayes approach to estimate variance. *P* values were adjusted for multiple comparisons using the Benjamini–Hochberg method. Multidimensional bioinformatics analyses were conducted, including volcano plots, Integrated Proteome-Lactylome Association Plot (Fold Change Comparison), KEGG pathway enrichment, GSEA, and subcellular localization analysis.

### Seahorse cellular energy metabolism analysis

BEAS-2B cells were seeded in a Seahorse XF96 cell culture microplate (W21715, Seahorse Biosciences). The OCR and ECAR were measured using a Seahorse XFe96 Analyzer (Agilent). For the mitochondrial stress test, 1.5 μM oligomycin, 0.5 μM carbonyl cyanide-p-trifluoromethoxyphenylhydrazone, and 0.5 μM rotenone/antimycin A were injected sequentially (Kit: Agilent, 103015–100). For the glycolysis rate assay, 0.5 μM rotenone/AA and 50 mM 2-deoxy-D-glucose were injected sequentially (Kit: Agilent, 103344-100).

### ΔΨm assay

Changes in the ΔΨm were assessed using the JC-1 fluorescent probe method. Briefly, cells from each treatment group were coincubated with culture medium containing 5 μM JC-1 (#C2006, Beyotime) for 15 min at 37 °C. After incubation, red (J-aggregates) and green (J-monomers) fluorescence images were captured using a Cytation 5 Multi-Mode Microplate Reader. The degree of mitochondrial depolarization was quantified by calculating the red-to-green fluorescence intensity ratio (Red/Green Ratio), with all data normalized to the control group.

### Cellular and histological analyses

#### ELISA

To assess the inflammatory response in the airway microenvironment, the concentrations of key cytokines (IL-4, IL-5, IL-13, and IL-17) in the BALF were quantified using commercial ELISA kits. Briefly, after the collection of BALF, the samples were centrifuged at 1,000 × g for 10 min at 4 °C to pellet the cells. The cell-free supernatants were collected and stored at −80 °C until analysis. The levels of IL-4, IL-5, IL-13, and IL-17 were measured using mouse-specific ELISA kits (Abcam, Cambridge, UK) strictly according to the manufacturer’s instructions. In brief, samples and standards were added to the precoated microplates and incubated. After washing, biotinylated detection antibodies and streptavidin-HRP were added sequentially. The reaction was developed using 3,3',5,5'-tetramethylbenzidine substrate and terminated with a stop solution. The OD was measured at 450 nm using a Synergy H1 microplate reader (Bio-Tek, Winooski, VT, USA). The cytokine concentrations were calculated based on the standard curves and expressed as nanograms per milliliter.

#### Flow cytometry

##### pHi detection

Cells were incubated with BCECF AM (a fluorescent pH probe, 10 μM working concentration) for 60 min at 37 °C in the dark. The fluorescence intensity was then measured using a flow cytometer (excitation wavelength: 488 nm, emission wavelength: 535 nm).

##### Cell population analysis

Single-cell suspensions from BALF, lymph nodes, or spleen were incubated with the following antibodies for 30 min at 4 °C in the dark: PE-Siglec-F (#552126; BD), APC-CD45.2 (#558702; BD Biosciences), PerCP-Cy5.5 CD11c (#45-0114-82; Invitrogen), PE-CD4 (#11-0041-82; Invitrogen), APC-IFN-γ (#554413; BD Biosciences), and PE-Cy7 IL-4 (#504117; Biolegend). Data were acquired using a Cytoflex flow cytometer (Beckman Colter, Inc., USA) and analyzed with CytoExpert 2.4 software. Eosinophils in BALF were identified as CD45.2^+^ CD11c^−^ Siglec-F^+^ populations.

### Histology and cell staining

#### Diff-quik staining

BALF cells were collected by centrifugation, fixed with formaldehyde, and then stained using a Diff-Quik reagent kit (#G1541, Solarbio, Beijing, China). Cell morphology was observed using a slide scanner imaging system (SQS-40R, Shengqiang Technology Co., Ltd., Shenzhen, China).

#### Histopathological staining

Paraffin-embedded lung tissue sections were subjected to hematoxylin and eosin staining,

PAS staining using a PAS staining kit (G1281, Solarbio, Beijing), and Masson’s trichrome staining using a Masson’s trichrome stain kit (G1345, Solarbio, Beijing).

#### Immunohistochemistry

Lung tissue sections were incubated overnight at 4 °C with primary antibodies, including PRMT5 (#MA532847, Thermo) and H4R3me2s (#PA596234, Thermo). This was followed by incubation with a goat anti-rabbit immunoglobulin G (IgG) (H+L) secondary antibody (#6721, 1:2,000, Abcam).

#### Immunofluorescence

To visualize the colocalization of target proteins with mitochondria, an immunofluorescence costaining technique was employed. Before immunostaining, mitochondria in live cells were labeled by incubating cells seeded in confocal dishes with a working solution of MitoTracker Red CMXRos (100 to 200 nM, #M7512, Thermo Fisher Scientific) in serum-free medium for 30 min at 37 °C in the dark. After mitochondrial labeling, cells were rinsed with PBS and then subjected to the immunofluorescence staining protocol. This protocol included the following: fixation with 4% PFA for 15 min at room temperature; permeabilization with 0.2% Triton X-100 for 10 min; and blocking of nonspecific binding with 5% bovine serum albumin for 1 h at room temperature. Subsequently, samples were incubated overnight at 4 °C with the following primary antibodies: Succinyllysine Rabbit pAb (#PTM-401, PTM BIO), Anti-L-Lactyl Lysine Rabbit mAb (#PTM-1401RM, PTM BIO), anti-PCK2 (#PTM-5650, PTM BIO), PRMT5 (#MA532847, Thermo), PQBP1 Polyclonal antibody (#16264-1-AP, Protech), H4R3me2s (#PA596234, Thermo), SCGB1A1 (#sc-365992, Santa Cruz Biotechnology), or Phospho-p38 MAPK (#4511, CST). The next day, after washing with PBS with Tween 20, samples were incubated for 1 h at room temperature in the dark with the corresponding fluorescent secondary antibodies (Alexa Fluor 488 goat anti-rabbit, #R37118, Life Technologies; or Goat Anti-Mouse IgG H&L Cy3, #97035, Abcam). For samples undergoing conventional immunofluorescence only, the MitoTracker Red incubation step was omitted, and the protocol began directly with PFA fixation. Finally, all samples were counterstained with 4′,6-diamidino-2-phenylindole and mounted using an antifade mounting medium. Images were acquired and analyzed using a Cytation 5 Imaging Reader and ImageJ software.

### Ultrastructural observation of cells

Cell samples were fixed in a 2.5% glutaraldehyde solution for 2 h at 4 °C. After rinsing with PBS, the samples were processed for standard electron microscopy sample preparation, including dehydration, embedding, ultrathin sectioning, and staining, which was outsourced to the Department of Pathology at Yanbian University. Finally, the cellular ultrastructure was observed and imaged using an HT7700 transmission electron microscope (Hitachi, Japan) at 6,000× magnification.

### Molecular interaction and gene expression analyses

#### Co-IP and interactomics

To identify interacting proteins and validate specific interactions, 2 strategies were employed:

#### Interactomics (IP-MS)

To identify PQBP1-interacting proteins, BEAS-2B cells overexpressing either Flag-PQBP1 or an empty vector were lysed, and immunoprecipitation was performed using anti-Flag M2 affinity gel (A2220, Sigma-Aldrich). The eluted protein complexes were briefly separated by SDS-polyacrylamide gel electrophoresis, followed by in-gel digestion and liquid chromatography-tandem mass spectrometry analysis. Proteins specifically binding to PQBP1 were identified by comparing them against the control group.

#### Co-IP validation

Co-IP assays were performed as previously described to validate the interaction between specific mutants. Briefly, Myc-tagged PRMT5 (WT or mutant) and Flag-tagged PQBP1 (WT or mutant) were cotransfected into HEK293T cells using Lipo3000 Transfection Reagent. Forty-eight hours posttransfection, cells were lysed using NETN lysis buffer (100 mM NaCl, 1 mM EDTA, 50 mM tris-HCl, pH 8.0, 0.5% NP-40) supplemented with protease inhibitors. Cell lysates were incubated with anti-Flag or anti-Myc magnetic beads (MedChemExpress) overnight at 4 °C. The immunoprecipitates were washed 4 times with NETN buffer, eluted with SDS loading buffer, and analyzed by Western blotting.

#### Endogenous immunoprecipitation for PTMs

To accurately evaluate the specific succinylation and lactylation levels of endogenous proteins in pHBECs, a large-scale endogenous immunoprecipitation strategy was applied. Briefly, cells from each HDM gradient group were lysed in radioimmunoprecipitation assay buffer supplemented with protease, deacetylase, and desuccinylase inhibitors to extract large quantities of total protein (1 to 2 mg per sample). The high-concentration lysates were incubated overnight at 4 °C with specific primary antibodies against PCK2 (#PTM-5650, PTM BIO) or PQBP1 (#16264-1-AP, Proteintech), with species-matched normal IgG serving as the negative control. Protein A/G magnetic beads were then added to capture the endogenous immune complexes. After extensive washing, the immunoprecipitated target proteins and corresponding input samples were eluted by boiling in SDS loading buffer. The PTM status of the captured proteins was subsequently analyzed by Western blotting using anti-Pan-Ksu or anti-Pan-Kla antibodies.

#### Nuclear and cytoplasmic fractionation

To detect the distribution of proteins in the nucleus and cytoplasm, subcellular fractionation was performed using the Nuclear and Cytoplasmic Protein Extraction Kit (#P0027, Beyotime Biotechnology) in strict accordance with the manufacturer’s instructions. The resulting nuclear and cytoplasmic protein fractions were used for subsequent Western blot analysis.

#### Western blot

Protein concentration was determined using a NanoPhotometer NP80 (Implen). A total of 20 μg of protein per sample was separated by 12% SDS-polyacrylamide gel electrophoresis and transferred to a polyvinylidene difluoride membrane. Primary antibodies were incubated overnight at 4 °C and included the following: anti-SLC38A2 (#ab90677, Abcam), anti-GLS (#12855, CST), anti-Succinyllysine Rabbit pAb (#PTM-401, PTMBIO), anti-PCK2 (#PTM-5650, PTMBIO), anti-Flag DYKDDDDK Tag (#14793S, CST), anti-ubiquitin (#134953, Abcam), anti-L-Lactyl Lysine Rabbit mAb (#PTM-1401RM, PTMBIO), PQBP1 Polyclonal antibody (#16264-1-AP, Proteintech), SIRT5 (#DF8294, Affinity), HDAC1 (#AF6433, Affinity), HDAC2 (#5113, CST), anti-PRMT5 (#ab109451, Abcam), WDR77 (#AB154190, Abcam), H4R3me2s (#PA596234, Thermo), Histone H4 (#CY5877, Abways), p-SAPK/JNK (T183/Y185) (#9255S, CST), JNK (#9252, CST), p38 MAPK (#8690, CST), Phospho-p38 MAPK (#4511, CST), p-Erk1/2 (#4370, CST), Erk1/2 (#4695, CST), and anti-β-actin (#3700s, CST). To quantify global protein succinylation and lactylation, the total signal intensity for each sample lane was integrated over the molecular weight range of 15 to 250 kDa, excluding the marker lane. Secondary antibodies, Goat Anti-Rabbit IgG H&L (#ab6721, Abcam) and Goat Anti-Mouse IgG H&L (#ab6789, Abcam), were incubated for 2 h at room temperature. Chemiluminescent signals were captured, and band intensities were quantified using Quantity One software (BioRad, Hercules, CA, USA). Densitometric measurements were performed with local background subtraction and confirmed to be within the linear detection range by analyzing serial dilutions of control samples. The densitometric intensity of each target protein was normalized to the corresponding β-actin loading control from the same blot. For the analysis of phosphorylated proteins (e.g., p-SAPK/JNK, p-p38, and p-Erk1/2), the phospho-protein signal was normalized to the corresponding total protein level. The relative intensity was calculated after background subtraction, and all experimental groups were expressed as a fold change relative to the control group, which was set to 1.0. Quality control criteria: Only blots with clear bands, minimal background (<10% of signal intensity), and no signs of saturation were included in quantitative analysis.

### RNA sequencing (RNA-seq) and qPCR

#### RNA-seq

Total RNA was extracted using the TRIzol reagent kit (Takara, Dalian, China). Transcriptome sequencing and data analysis were performed by Novogene Co., Ltd. (Tianjin, China) on an Illumina NovaSeq 6000 platform. Differentially expressed genes were identified using the “limma” package in R (false discovery rate [FDR] < 0.05, |log2(fold change) | > 1), followed by Gene Ontology, KEGG, and GSEA enrichment analyses.

#### RT-qPCR

Total RNA was isolated using the RNA Easy Fast Animal Tissue/Cell Total RNA Extraction Kit (#DP451; TIANGEN). cDNA was synthesized using the FastKing gDNA Dispelling RT SuperMix (#KR118; TIANGEN). qPCR analysis was performed using SuperReal PreMix Plus (SYBR Green) (#FP205; TIANGEN) on an Azure Cielo 6 system (Azure Biosystems). Primer sequences are listed in Table S2. Relative gene expression levels were calculated using the 2^−^ΔΔCt method and normalized to glyceraldehyde phosphate dehydrogenase (GAPDH) as an internal control.

### ChIP and subsequent analyses

Cells were cross-linked with 1% formaldehyde, and chromatin was sheared into fragments with an average length of 200 to 1,000 bp by sonication. The processed chromatin fragments were immunoprecipitated with either an anti-Histone H4R3me2s antibody (#61187, Proteintech) or rabbit IgG as a negative control. An aliquot of the chromatin that did not undergo immunoprecipitation was saved as the input control. The immunoprecipitated complexes were eluted, reverse cross-linked, and digested with proteinase K. The DNA was then purified and used for subsequent ChIP-seq or ChIP-qPCR analysis.

For ChIP-seq analysis, the purified DNA was used to construct sequencing libraries, which were subjected to high-throughput sequencing on an Illumina platform, with a sequencing depth of at least 20 million reads per sample. The sequencing data were aligned using Bowtie2. Peak calling was performed using MACS2. Subsequent analyses were performed using software including bedtools and deepTools. For differential binding analysis, consensus peak sets were generated, and read counts were normalized using the DiffBind R package. Differentially bound regions were identified using the DESeq2 method (based on a negative binomial distribution model) with an FDR < 0.05 cutoff.

To validate the ChIP-seq enrichment results, real-time quantitative PCR was performed on the purified DNA (from the experimental, IgG, and input groups). We designed primers targeting the promoter regions of several key target genes and successfully validated the enrichment for *SMO*, *MAP3K*10, *CTNNB1*, *GABRP*, *SLC2A12*, and *PDE4B*. Two other candidate genes identified by ChIP-seq, *PFN2* and *EPHB6*, were not validated further because specific and efficient primers could not be designed due to the high homology of PFN2 with other genomic regions and the low GC content in the target region of EPHB6, respectively. The binding level of the target protein at specific DNA loci was quantitatively analyzed by calculating the percentage of enrichment in the immunoprecipitated sample relative to the total input sample (% Input) and comparing the enrichment efficiency between the specific antibody group and the IgG control group.

### Bioinformatics analysis of human public transcriptome datasets

To validate the clinical relevance of the PCK2–lactate–PQBP1 axis, we retrieved and analyzed public transcriptomic datasets from the Gene Expression Omnibus database. For bulk transcriptomic analysis, the gene expression profile GSE43696 was obtained to assess the mRNA levels of metabolic and epigenetic regulators in bronchial epithelial brushings from healthy controls (*n* = 20) and patients with severe asthma (*n* = 36). The normalized gene expression matrix was downloaded directly from the Gene Expression Omnibus database. Probes corresponding to the genes of interest—specifically *PCK2*, *HK2*, *EP300*, *PQBP1*, and PRMT5—were extracted, and their average expression values were calculated if multiple probes corresponded to a single gene. Statistical differences in gene expression between the healthy and severe asthma groups were analyzed using an unpaired Student *t* test, and data visualization was performed using the ggplot2 package in R software (Version 4.2.0).

To further investigate cell-type-specific gene expression, particularly that of the lactyltransferase EP300 and the key glutamine metabolic regulators (SLC38A2 and GLS), we utilized the scRNA-seq dataset GSE193816, which comprises bronchial epithelial cells from asthmatic patients and healthy donors. The raw gene expression matrix was processed using the Seurat R package (Version 4.2.0). For quality control, cells were filtered to retain those with unique feature counts between 200 and 6,000 and less than 20% mitochondrial gene content to exclude low-quality cells and doublets. Following normalization via the Normalize Data function, the top 2,000 highly variable features were identified. Dimensionality reduction was performed using principal component analysis, and uniform manifold approximation and projection was subsequently employed to visualize cellular heterogeneity and cluster distribution. Epithelial cell clusters were identified based on the expression of canonical marker genes (e.g., *EPCAM* and *KRT5*). Finally, the differential expression levels of target genes (*EP300*, *SLC38A2*, and *GLS*) in epithelial cell clusters were compared between the asthma and control groups using the Wilcoxon rank-sum test, with results visualized as box plots.

#### Candidate gene screening

Key lactate-responsive genes that regulate glutaminolysis were identified by performing an intersection analysis of search results from the Comparative Toxicogenomics Database and GeneCards with the proteomics data generated in this study.

#### Protein sequence conservation and structural analysis

To evaluate the evolutionary conservation of key lysine residues (e.g., PCK2-K100 and PQBP1-K223), homologous protein sequences from multiple species were obtained from National Center for Biotechnology Information. These sequences were aligned using ClustalW and then submitted to the ConSurf server to calculate conservation scores. The results were subsequently mapped onto the 3-dimensional protein structures, obtained from the Protein Data Bank (PDB) database, for visualization.

#### Molecular docking simulation

Preprocessing of the 3-dimensional structures of PRMT5 (PDB ID: 6RLL) and PQBP1 (PDB ID: 4CDO) was performed using PyMOL software. Protein–protein docking was conducted using the ZDOCK server to predict the binding mode between PRMT5 and PQBP1, followed by conformational optimization via RosettaDock. The optimal binding pose was selected for interface analysis based on the lowest binding energy and geometric shape complementarity. Key interacting residues were identified based on hydrogen bond distances of less than 3.5 A and electrostatic complementarity. All structural images were generated using PyMOL software.

#### Protein structure prediction and molecular dynamics simulation

The initial structure of the target PQBP1 complex was predicted using AlphaFold3 [54], and the model with the highest score was selected for subsequent studies. To investigate the impact of lysine lactylation, 2 simulation systems were constructed: a WT system and a lactylated system (PQBP1-KLA). System building and solvation were performed using the CHARMM-GUI server, employing the CHARMM36m force field [55] and the TIP3P water model. The systems were neutralized with 0.15 M counterions. All molecular dynamics simulations were performed using GROMACS 2023.5. Each system underwent energy minimization and a 2-stage equilibration (NVT and NPT) before being subjected to 3 independent 100-ns production simulations. By analyzing the simulation trajectories, we calculated the root-mean-square deviation of the protein backbone, root-mean-square fluctuation, radius of gyration, number of intersubunit hydrogen bonds, and the Gibbs free energy landscape to comprehensively assess the impact of lactylation on the conformational stability, local flexibility, and dynamic features of the PQBP1 protein.

### Statistical analysis

Data are presented as means ± standard error of the mean (SEM) from at least 3 independent experiments. Statistical significance between 2 groups was analyzed using an unpaired, 2-tailed Student *t* test. Comparisons among 3 or more groups with a single independent variable were performed using 1-way analysis of variance (ANOVA) followed by Tukey’s multiple comparisons post hoc test. For experiments involving 2 independent variables (such as time-course assays, AHR across varying methacholine concentrations, and metabolic measurements with sequential drug injections), a 2-way ANOVA followed by Tukey’s or Sidak’s multiple comparisons post hoc test was performed. For all omics data (proteomics, ChIP-seq, and RNA-seq), *P* values were adjusted for multiple comparisons using the Benjamini–Hochberg method to control the FDR. A *P* value of less than 0.05 was considered statistically significant. All analyses were performed using GraphPad Prism 9 and R software.

## Data Availability

The multiomics datasets generated during this study are publicly available in the following repositories: The RNA-seq data (accession: HRA014618) and ChIP-seq data (accession: HRA014608) are deposited in the NGDC Genome Sequence Archive (GSA). The mass spectrometry data, including the IP-MS proteomics dataset (accession: PXD070678) and the PTM-proteomics (lactylation and succinylation) dataset (accession: PXD070618), are available via the ProteomeXchange Consortium (iProX partner repository).
