# Supplementary material for: PCK2-Mediated PQBP1 Lactylation Promotes Asthmatic Inflammation through PRMT5 Inhibition
Source: Research (Wash D C). 2026 Jun 19;9:1321. doi: 10.34133/research.1321 (PMC13280573; doi:10.34133/research.1321)

A

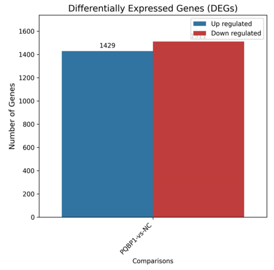

B

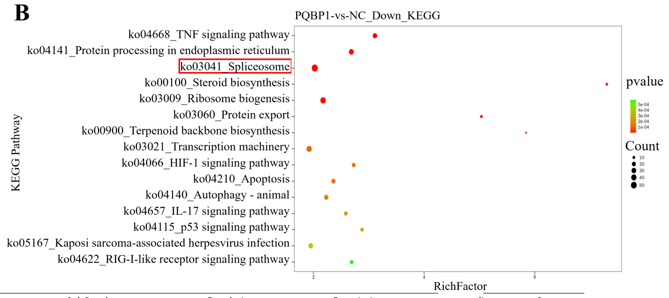

C

KEGG_Pathway_ID	Ontology	SubOntology	Regulation	Description	p.adjust	Count
ko03041	Transcription	Spliceosome_2	Down	Spliceosome	3.48E-05	57
ko03021	Transcription	Transcription machinery	Down	Transcription machinery	0.004397	37
ko03040	Transcription	Spliceosome_1	Down	Spliceosome	0.034271	21

D

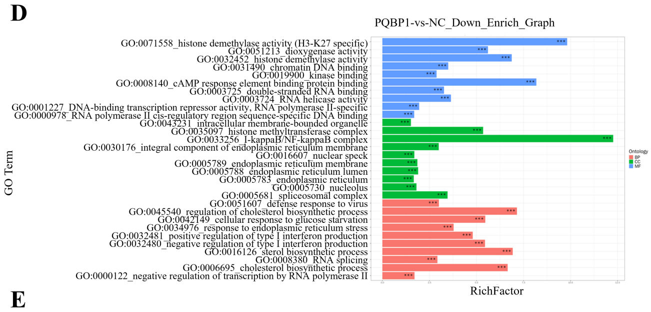

F

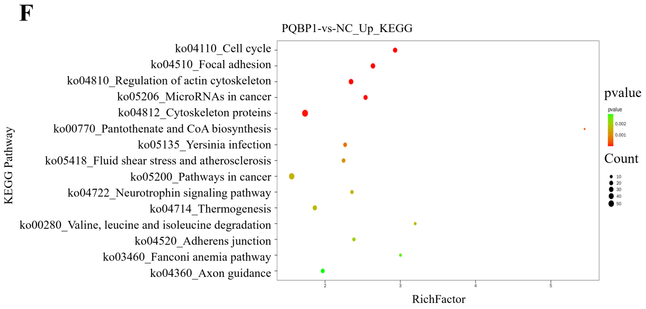

E

KEGG_Pathway_ID	Ontology	SubOntology	Regulation	Description	p.adjust	Count
ko04141	Vesicle_transport	Protein processing in endoplasmic reticulum	Down	Protein processing in endoplasmic reticulum	3.48E-05	32
ko03060	Vesicle_transport	Protein export	Down	Protein export	0.000891	10
ko04140	Vesicle_transport	Autophagy - animal	Down	Autophagy - animal	0.005804	24
ko02044	Vesicle_transport	Secretion system	Down	Secretion system	0.023548	6

G

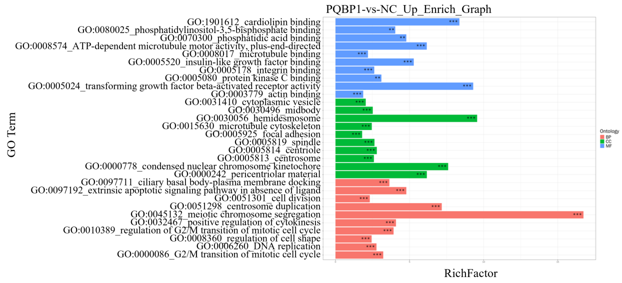

H

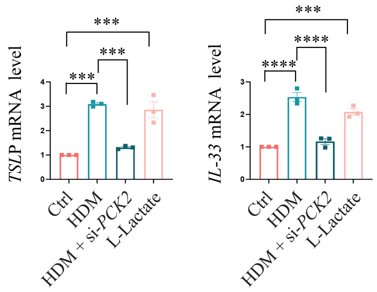

Supplement: Supplementary 1 — Figs. S1 to S20 Tables S1 and S2 [file research.1321.f1.zip › Figure S15.pdf]
