# Supplementary material for: PCK2-Mediated PQBP1 Lactylation Promotes Asthmatic Inflammation through PRMT5 Inhibition
Source: Research (Wash D C). 2026 Jun 19;9:1321. doi: 10.34133/research.1321 (PMC13280573; doi:10.34133/research.1321)
Supplement: Supplementary 1 — Figs. S1 to S20 Tables S1 and S2 [file research.1321.f1.zip › Supplemental Material Table 1.docx]

**Supplementary Table S1: List of primers used for site-directed mutagenesis**

| **Primer Name** | **Sequence (5' to 3')** | **Description** |
| --- | --- | --- |
| PQBP1-Mut1-F | 5'- CTC CGT CTG GTG GCG AAG GCG GCG CGC GCT GAG -3' | R121A / R124A / K125A (Forward) |
| PQBP1-Mut1-R | 5'- CTC AGC GCG CGC CGC CTT CGC CAC CAG ACG GAG -3' | R121A / R124A / K125A (Reverse) |
| PQBP1-Mut2-F | 5'- GAT AAG AGC CTG GCG GCG TAC AGC GGC -3' | K71A / E74A (Forward) |
| PQBP1-Mut2-R | 5'- GCC GCT GTA CGC CGC CAG GCT CTT ATC -3' | K71A / E74A (Reverse) |
| PRMT5-MutA-R1-F | 5'- AAG GGG GCG ATC CCC GCG AAC CAG CTG -3' | Y304A / Y307A (Round 1 Forward) |
| PRMT5-MutA-R1-R | 5'- CAG CTG GTT CGC GGG GAT CGC CCC CTT -3' | Y304A / Y307A (Round 1 Reverse) |
| PRMT5-MutA-R2-F | 5'- TTC ATC GCG TCT CAG ACA GCC CTG TAC -3' | E320A (Round 2 Forward) |
| PRMT5-MutA-R2-R | 5'- GTA CAG GGC TGT CTG AGA CGC GAT GAA -3' | E320A (Round 2 Reverse) |
| PRMT5-MutB-F | 5'- TCC AGC GCG CTG AAC AAC TTC -3' | D70A (Forward) |
| PRMT5-MutB-R | 5'- GAA GTT GTT CAG CGC GCT GGA -3' | D70A (Reverse) |
