# Supplementary material for: PCK2-Mediated PQBP1 Lactylation Promotes Asthmatic Inflammation through PRMT5 Inhibition
Source: Research (Wash D C). 2026 Jun 19;9:1321. doi: 10.34133/research.1321 (PMC13280573; doi:10.34133/research.1321)
Supplement: Supplementary 1 — Figs. S1 to S20 Tables S1 and S2 [file research.1321.f1.zip › Supplemental MaterialTable 2.docx]

**Supplementary Table S2. Primer sequences.**

| **Primer Name** | **Sequence (5' to 3')** |
| --- | --- |
| hsa- SLC2A12-forward | 5′-CCATGGCTGGAAGTACATGTTTGG-3′ |
| hsa - SLC2A12-reverse | 5′-CTGAGAGTGCTCTTAACCTTCC-3′ |
| hsa - PDE4B-forward | 5′-ATGGTGTTAGCAACTGATATG-3′ |
| hsa - PDE4B-reverse | 5′-AGAACGCCTGAACTTGTA-3′ |
| hsa -GAPDH-forward | 5′-TGCACCACCAACTGCTTAGC-3′ |
| hsa -GAPDH-reverse | 5′-GGCATGGACTGTGGTCATGAG-3′ |
| hsa -MAP3K10-forward | 5′-GAACGTGAGATGGACATCGTGGAA-3′ |
| hsa -MAP3K10-reverse | 5′-AGGCCTGGACTGTGATCTTATGCT-3′ |
| hsa -CTNNB1-forward | 5′-GACCACAAGCAGAGTGCTGA-3′ |
| hsa -CTNNB1-reverse | 5′-CTTGCATTCCACCAGCTTCT-3′ |
| hsa -PFN2-forward | 5′-GCTACGTGGATAACCTGATGTGCG-3′ |
| hsa -PFN2-reverse | 5′-CCCCTAATACTTAACAGTCTGCCTAGC-3′ |
| hsa -GABRP-forward | 5′-CAGACCCACGGCTAGTGTTC-3′ |
| hsa -GABRP-reverse | 5′-AGAGGCGGATGAGCCTGTT-3′ |
| hsa -SMO-forward | 5′-TTCTTCAACCAGGCTGAGTG-3′ |
| hsa - SMO-reverse | 5′- CGTATGGCTTCTCATTGGAGTG-3′ |
| hsa -EPHB6-forward | 5′-TCCTGGTGTCTTCAGTTCTG-3′ |
| hsa -EPHB6-reverse | 5′- GAAGCAAAGGATCGGAGCAC-3′ |
| hsa -TSLP-forward | 5′- CCCTTCACTCCCCGACAAAA -3′ |
| hsa - TSLP -reverse | 5′- CCTGAGTACCGTCATTTCTCTCA -3′ |
| hsa -IL-33 -forward | 5′- GAGAAATCACGGCAGAATCA -3′ |
| hsa - IL-33 -reverse | 5′- CTTCTTATTTTGCAAGGCGG -3′ |
| hsa -SLC38A2-forward | 5′-GTGTCCTGTGGAAGCTGCTTTGA |
| hsa -SLC38A2-reverse | 5′-CAGGTACAAGAGCTGTTGGCTGTGT-3′ |
| mmu- SLC38A2-forward | 5′-CCGCGGGCGACAATAAAATAA-3′ |
| mmu- SLC38A2-reverse | 5′-CAGGTACAAGAGCTGTTGGCTGTGT-3′ |
| has-GLS-forward | 5′-TGGTGGCCTCAGGTGAAAAT-3′ |
| has-GLS-reverse | 5′-CCAAGCTAGGTAACAGACCCTGTTT-3′ |
| mmu-GLS-forward | 5′-CCGCGGGCGACAATAAAATAA-3′ |
| mmu- GLS-reverse | 5′-GCATGACACCATCTGACGTT-3′ |
| mmu-GAPDH-forward | 5′-TCATGGATGACCTTGGCCAG-3′ |
| mmu-GAPDH-reverse | 5′-GTCTTCACTACCATGGAGAAGG-3′ |
| mmu-IL-4-forward | 5′-AGGAGCCATATCCACGGATG-3′ |
| mmu-IL-4-reverse | 5′-ACAGACGAGCTCACTCTCTG-3′ |
| mmu-IL-5-forward | 5′-GCAATGAGACGATGAGGCTT-3′ |
| mmu-IL-5-reverse | 5′-CCCACGGACAGTTTGATTCT-3′ |
| mmu-IL-13-forward | 5′-CAGCTCCCTGGTTCTCTCAC-3′ |
| mmu-IL-13-reverse | 5′-ACACTCCATACCATGCTGCC-3′ |
| mmu-TNF-α-forward | 5′-AGGGTCTGGGCCATAGAACT-3′ |
| mmu-TNF-α-reverse | 5′-CAGCCTCTTCTCATTCCTGC-3′ |
| mmu-IL-1β-forward | 5′-TGGCAACTGTTCCTG-3′ |
| mmu-IL-1β-reverse | 5′-GGAAGCAGCCCTTCATCTTT-3′ |
