# Supplementary material for: PCK2-Mediated PQBP1 Lactylation Promotes Asthmatic Inflammation through PRMT5 Inhibition
Source: Research (Wash D C). 2026 Jun 19;9:1321. doi: 10.34133/research.1321 (PMC13280573; doi:10.34133/research.1321)
Supplement: Supplementary 1 — Figs. S1 to S20 Tables S1 and S2 [file research.1321.f1.zip › supplementary_materials .docx]

Supplementary Materials for

PCK2-mediated PQBP1 lactylation promotes asthmatic inflammation through PRMT5 inhibition

Qiaoyun Bai *et al*.

* Corresponding authors:

Guanghai Yan,

Department of Anatomy, Histology and Embryology, Yanbian University Medical College, No. 977, Gongyuan Road, Yanji 133002, P. R. China. Tel: +86-433-243-5137. Fax: +86-433-243-5136. E-mail: ghyan2015@sina.com & ghyan@ybu.edu.cn

Yilan Song,

Department of Anatomy, Histology and Embryology, Yanbian University Medical College, No. 977, Gongyuan Road, Yanji 133002, P. R. China. Tel: +86-433-243- 5135. Fax: +86-433-243-5135. E-mail: [songyl@ybu.edu.cn](mailto:songyl@ybu.edu.cn)

Chongyang Wang,

Department of Anatomy, Histology and Embryology, Yanbian University Medical College, No. 977, Gongyuan Road, Yanji 133002, P. R. China. Tel: +86-433-243-5134. Fax: +86-433-243-5134. E-mail: [wcy@ybu.edu.cn](mailto:wcy@ybu.edu.cn)


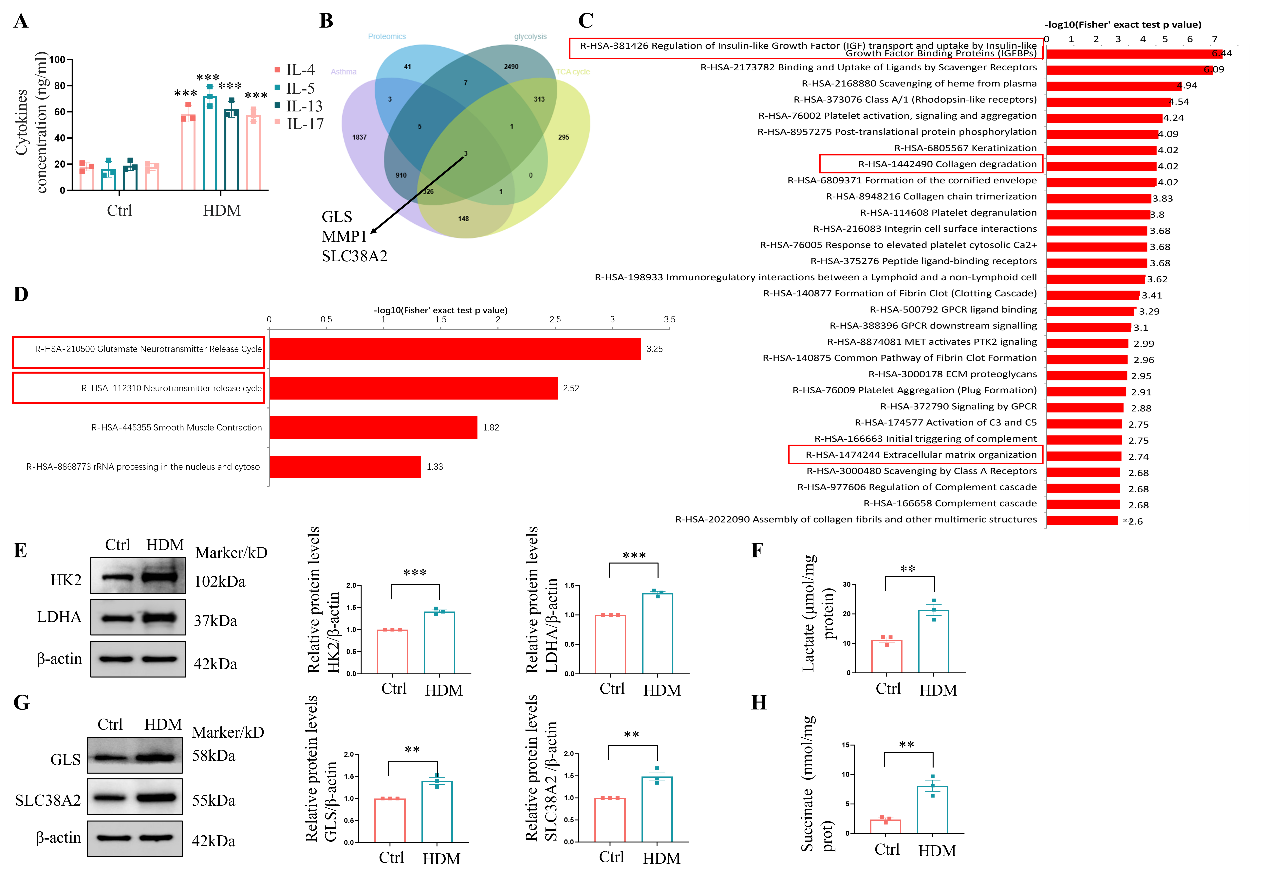


Fig. S1.

Combined bioinformatics and proteomics analysis identifies key pathways. (A) Analysis of inflammatory cytokines in BALF. The concentrations of IL-4, IL-5, IL-13, and IL-17 in the BALF supernatant were measured by ELISA. (B) Venn diagram showing the intersection analysis of database searches (CTD, GeneCards) and proteomics data. (C, D) Pathway enrichment analysis of differentially expressed proteins. (E) Western blot analysis of glycolytic enzymes HK2 and LDHA in BEAS-2B cells treated with HDM. (F) Lactate production levels in the culture medium. (G) Western blot analysis of glutaminolysis markers GLS and SLC38A2. (H) Intracellular succinate levels. Data are presented as mean ± SEM (n=3 independent experiments). Analyzed by Student's t-test. ** P < 0.01, ***P < 0.001.


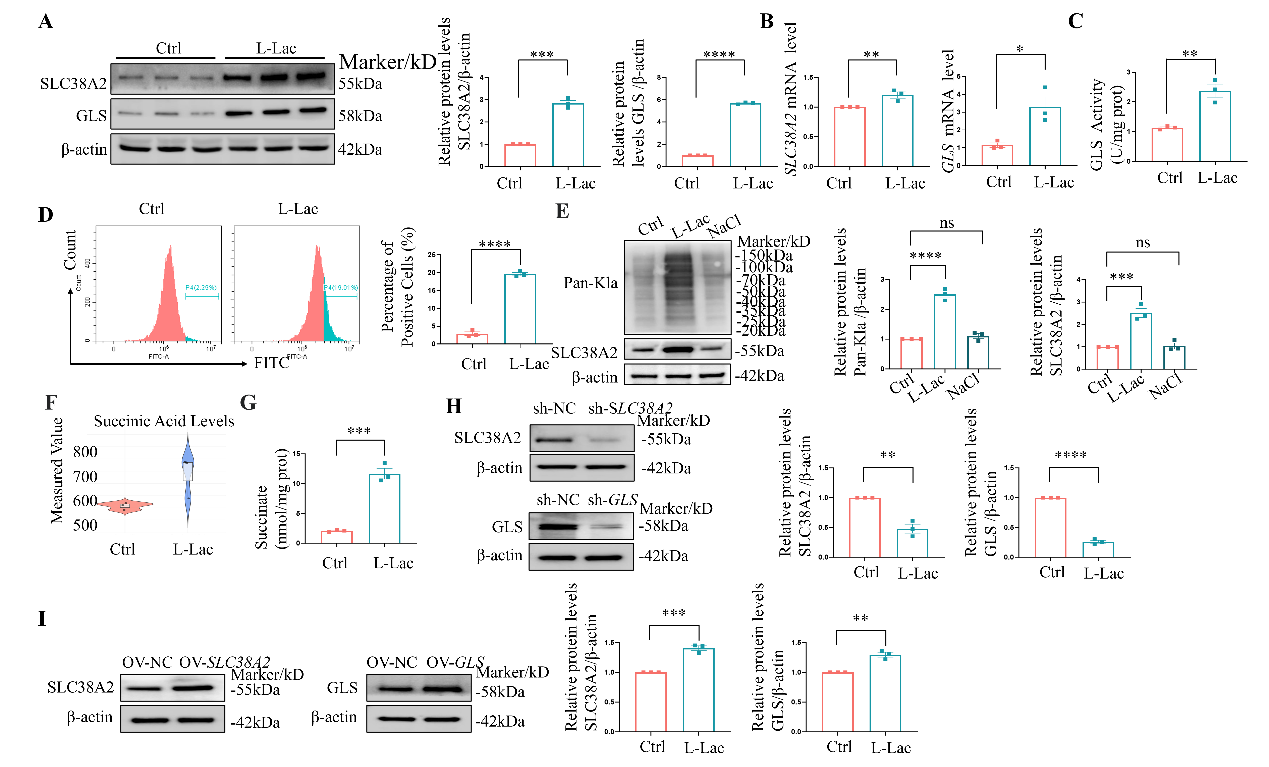


Fig. S2.

In vitro effects of lactate on the SLC38A2/GLS pathway and succinate levels. BEAS-2B cells were treated with lactate, followed by analysis of: (A, B) SLC38A2/GLS protein (A, Western blot) and mRNA (B, qPCR) levels of *SLC38A2*/*GLS*. (C) GLS activity. (D) Intracellular pH (pHi) by flow cytometry. (E) Western blot analysis of global protein lactylation (Pan-Kla) and SLC38A2 expression in cells treated with Lactate or NaCl (osmotic control). β-actin served as the loading control. (F, G) Intracellular succinate levels by untargeted metabolomics (F) and targeted assay (G). (H, I) Validation of genetic manipulation efficiency. BEAS-2B cells were transfected with specific shRNAs (sh-SLC38A2, sh-GLS) or overexpression plasmids (OV-SLC38A2, OV-GLS) and their respective negative controls (sh-NC, OV-NC) for 48 h. Western blot analysis and densitometric quantification show the significant downregulation (H) or upregulation (I) of SLC38A2 and GLS protein levels. All Western blots are representative of ≥3 independent experiments. Data are presented as mean ± SEM (n=3 independent experiments). Analyzed by Student's t-test. *P < 0.05, **P < 0.01, ***P < 0.001, ****P < 0.0001.


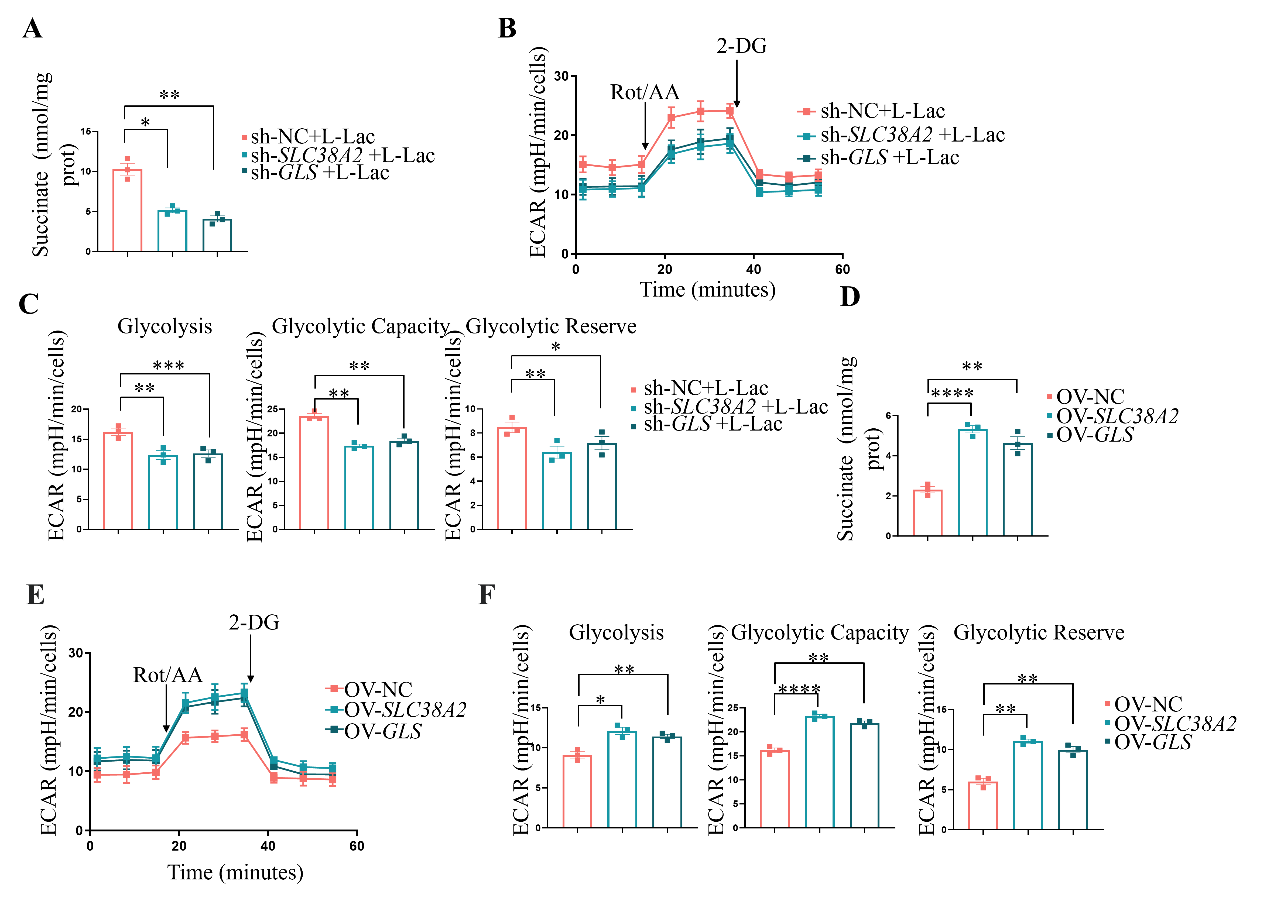


Fig. S3.

Genetic intervention validates the role of the SLC38A2/GLS pathway. (A-C) Effects of shRNA-mediated knockdown of *SLC38A2 or GLS* on succinate accumulation (A) and ECAR (B, C). (D-F) Effects of overexpressing *SLC38A2 or GLS* on succinate levels (D) and ECAR (E, F). Data are presented as mean ± SEM (n=3 independent experiments). Analyzed by one-way ANOVA. *P < 0.05, **P < 0.01, ****P < 0.0001.


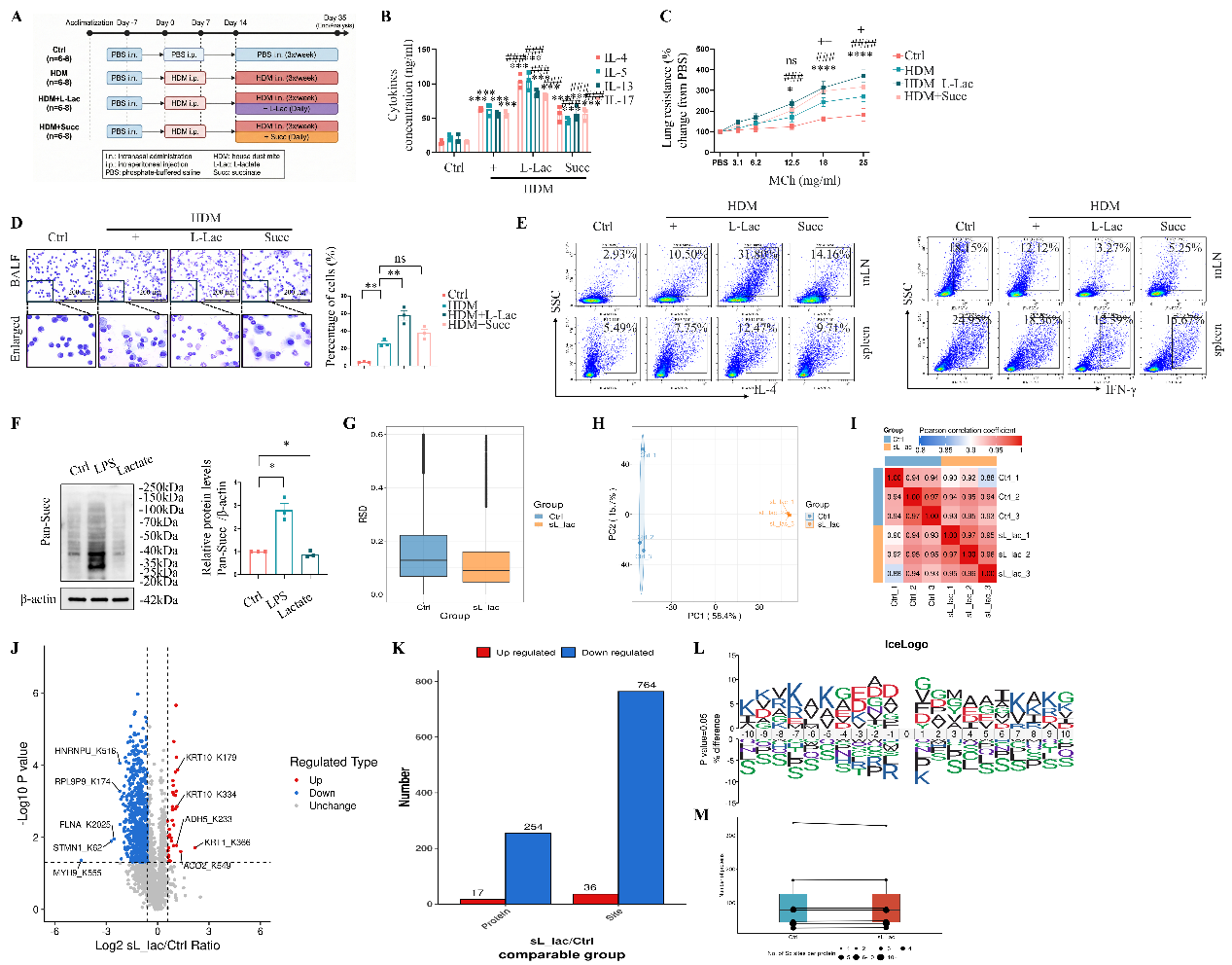


Fig. S4.

In vivo functional experiments and succinyl-proteomics analysis. (A) Schematic of the experimental protocol for exogenous lactate and succinate supplementation in HDM-induced asthmatic mice. C57BL/6 mice were randomized into four groups: Ctrl, HDM, HDM+L-Lac, and HDM+Succ. (B) ELISA analysis of inflammatory cytokines (IL-4, IL-5, IL-13, and IL-17) in the BALF supernatant. (C) Exogenous lactate or succinate exacerbates airway hyperresponsiveness (AHR). Lung resistance (% change from PBS) was measured in response to increasing concentrations of methacholine (MCh). (D) Representative images of Diff-Quik staining of BALF cells (left) and quantification of eosinophil percentage (right). Scale bar, 200 μm. (E) Representative flow cytometry plots of Th2 (IL-4⁺) and Th1 (IFN-γ⁺) cells in the mediastinal lymph nodes (mLN) and spleen. (F) Western blot analysis of global protein succinylation levels (Pan-Succ) in control (Ctrl), LPS-treated, and Lactate-treated BEAS-2B cells. β-actin served as a loading control. Bar graphs show the relative protein levels. (G-I) Quality control of succinyl-proteomics data, including RSD distribution (G), PCA analysis (H), and Pearson correlation analysis (I). (J, K) Volcano plot (J) and statistical summary (K) of differentially succinylated proteins/sites (sL_lac vs. Ctrl). (L, M) Motif analysis (L) and distribution of succinylation sites per protein (M). Data are presented as mean ± SEM (n=6–8 per group for in vivo, and n=3 independent experiments for in vitro). Analyzed by one-way ANOVA or Student's t-test. Symbols indicate statistical significance compared to the indicated groups (* vs. Ctrl; # vs. HDM; + vs. HDM+L-Lac/Succ). *P < 0.05, **P < 0.01, ***P < 0.001, ****P < 0.0001; ns, not significant.


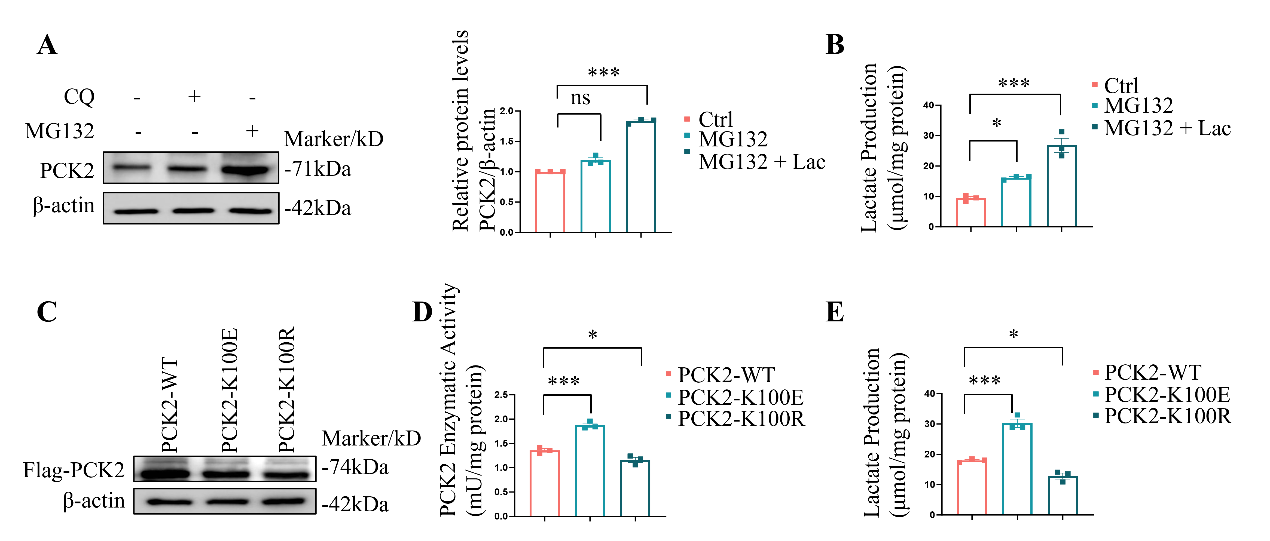

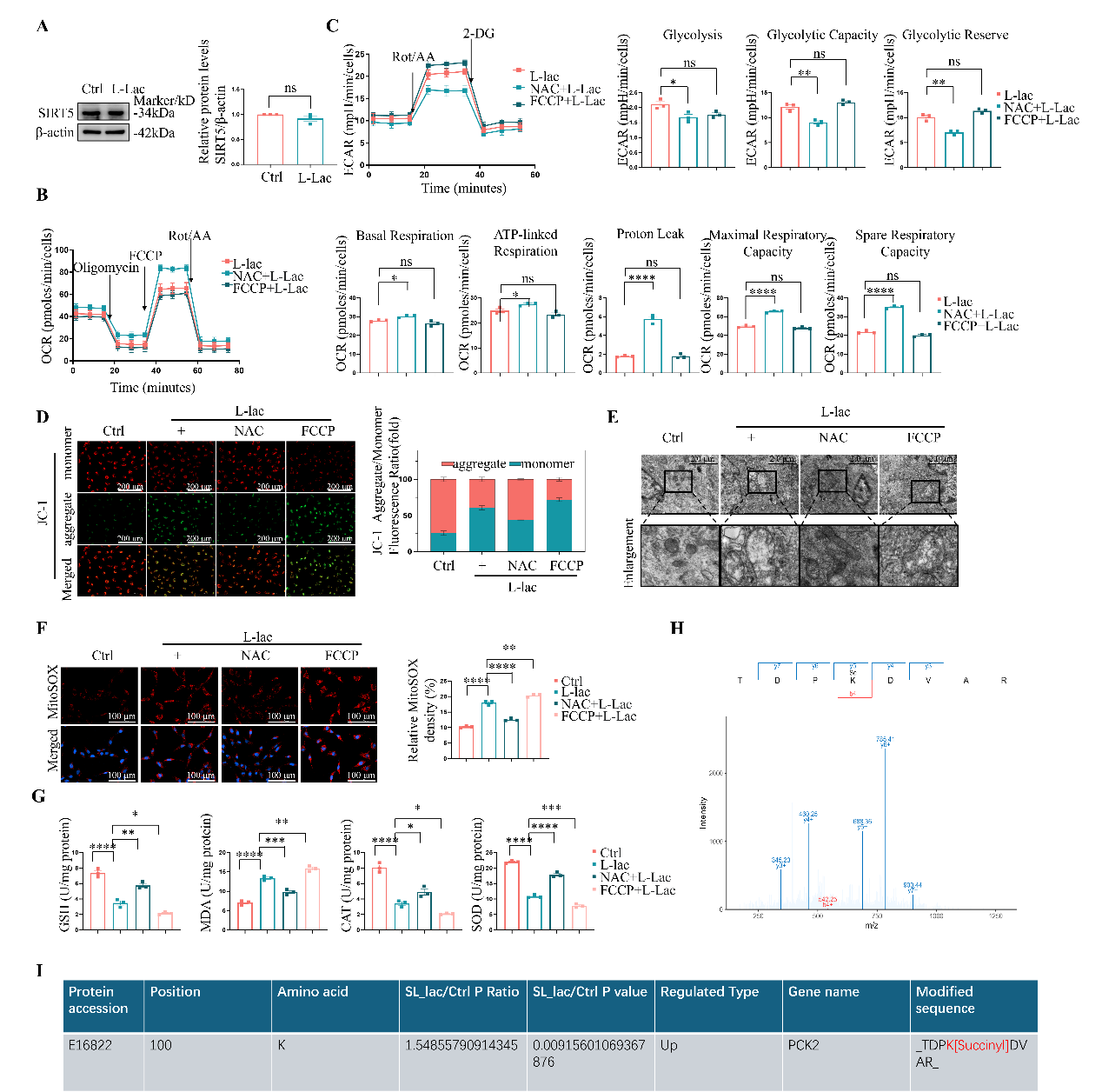


Fig. S5.

Effect of lactate on mitochondrial function and mass spectrometric identification of PCK2-K100 succinylation. (A) Western blot analysis of SIRT5 expression in BEAS-2B cells treated with or without Lactate (L-Lac). Quantitative analysis of SIRT5 band intensity relative to β-actin. (B, C) Seahorse analysis of OCR (B) and ECAR (C) in lactate-treated cells treated with NAC or FCCP. (D) Assessment of mitochondrial membrane potential (ΔΨm) by JC-1 staining. Scale bar, 200 μm. (E) TEM images of mitochondrial ultrastructure. Scale bar, 2.0 μm. (F) Detection of mitochondrial ROS (mtROS) by MitoSOX staining. Scale bar, 100 μm. (G) Quantification of oxidative stress markers (GSH, CAT, SOD, MDA). (H, I) Mass spectrometric identification data for PCK2-K100 succinylation, including the MS/MS spectrum (H) and site information (I). Data are presented as mean ± SEM (n=3 independent experiments). Analyzed by one-way ANOVA. *P < 0.05, **P < 0.01, ***P < 0.001, ****P < 0.0001; ns, not significant.


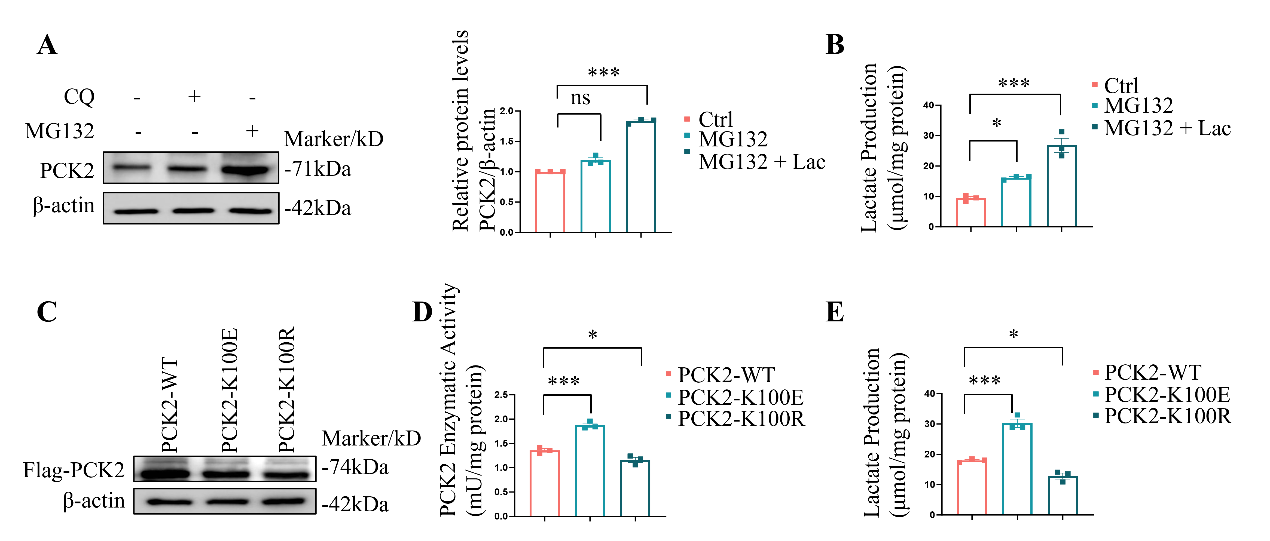


Fig. S6.

PCK2 degradation pathway and K100-dependent enzymatic activation. (A) Western blot analysis of PCK2 levels in BEAS-2B cells treated with chloroquine (CQ) or MG132, and quantification of protein levels under MG132 treatment alone or combined with lactate. (B) Intracellular lactate levels in BEAS-2B cells treated with MG132 alone or combined with lactate (10 mM). (C) Western blot validation of Flag-PCK2 (WT, K100E, and K100R) expression in HEK293T cells. (D) In vitro enzymatic activity of purified PCK2 proteins (mU/mg protein). (E) Lactate production in the culture medium of HEK293T cells expressing the indicated plasmids (μmol/mg protein). Data are presented as mean ± SEM (n=3 independent experiments). Analyzed by Student's t-test or one-way ANOVA. * P < 0.05, ***P < 0.001; ns, not significant.


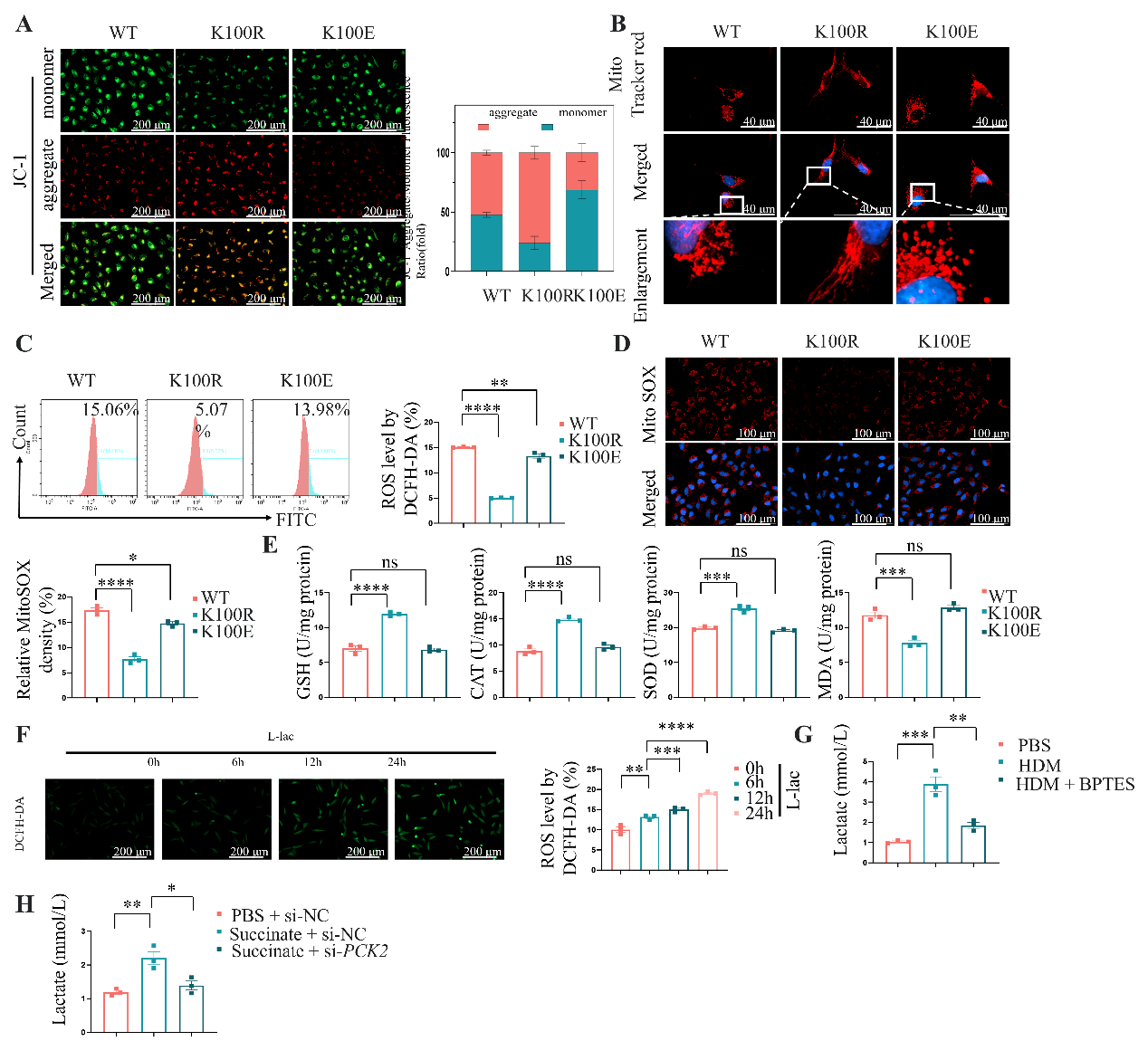


Fig. S7.

Effect of PCK2-K100 succinylation on mitochondrial and cellular oxidative stress. (A-E) Analysis of cells expressing WT or mutant (K100R/E) PCK2, showing: mitochondrial membrane potential. Scale bar, 200 μm. (A); mitochondrial network morphology. Scale bar, 40 μm. (B); total ROS and mtROS. Scale bar, 100 μm.(C, D); and oxidative stress markers (E). (F) Time-course analysis of total ROS levels following lactate treatment. Scale bar, 200 μm. (G) Intracellular lactate levels in HDM-stimulated BEAS-2B cells treated with or without the GLS inhibitor BPTES. (H) Intracellular lactate levels in BEAS-2B cells following exogenous succinate supplementation, with or without *PCK2* knockdown (si-*PCK2*). Data are presented as mean ± SEM (n=3 independent experiments). Analyzed by one-way ANOVA. *P < 0.05, **P < 0.01, ***P < 0.001, ****P < 0.0001; ns, not significant.


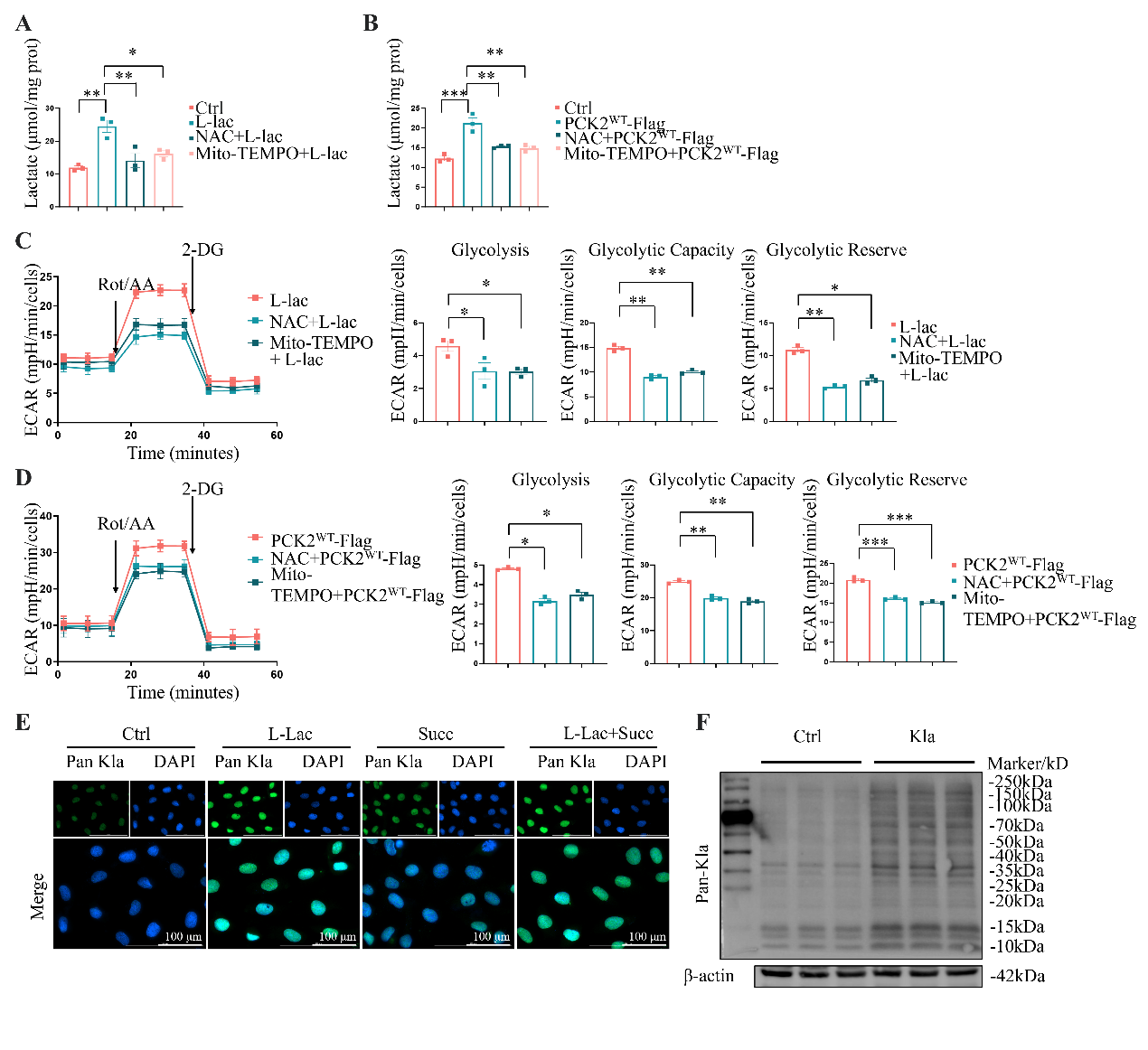


Fig. S8.

Role of oxidative stress in the PCK2-lactate feedback loop and nuclear protein lactylation. (A-D) Effects of antioxidant pre-treatment (NAC or Mito-TEMPO) on lactate production (A, B) and glycolytic function (ECAR) (C, D) induced by lactate stimulation (A, C) or PCK2 wild-type (PCK2^WT-Flag) overexpression (B, D). (E) Immunofluorescence analysis of the subcellular localization of protein lactylation (Pan-Kla, green) in the Control, Lactate (L-Lac), Succinate (Suc), and combined treatment (L-Lac+Su) groups. Scale bar, 100 μm. (F) Western blot analysis of global protein lactylation levels (Pan-Kla). All Western blots are representative of ≥3 independent experiments. Data are presented as mean ± SEM (n=3 independent experiments). Analyzed by one-way ANOVA. *P < 0.05, **P < 0.01, ***P < 0.001.


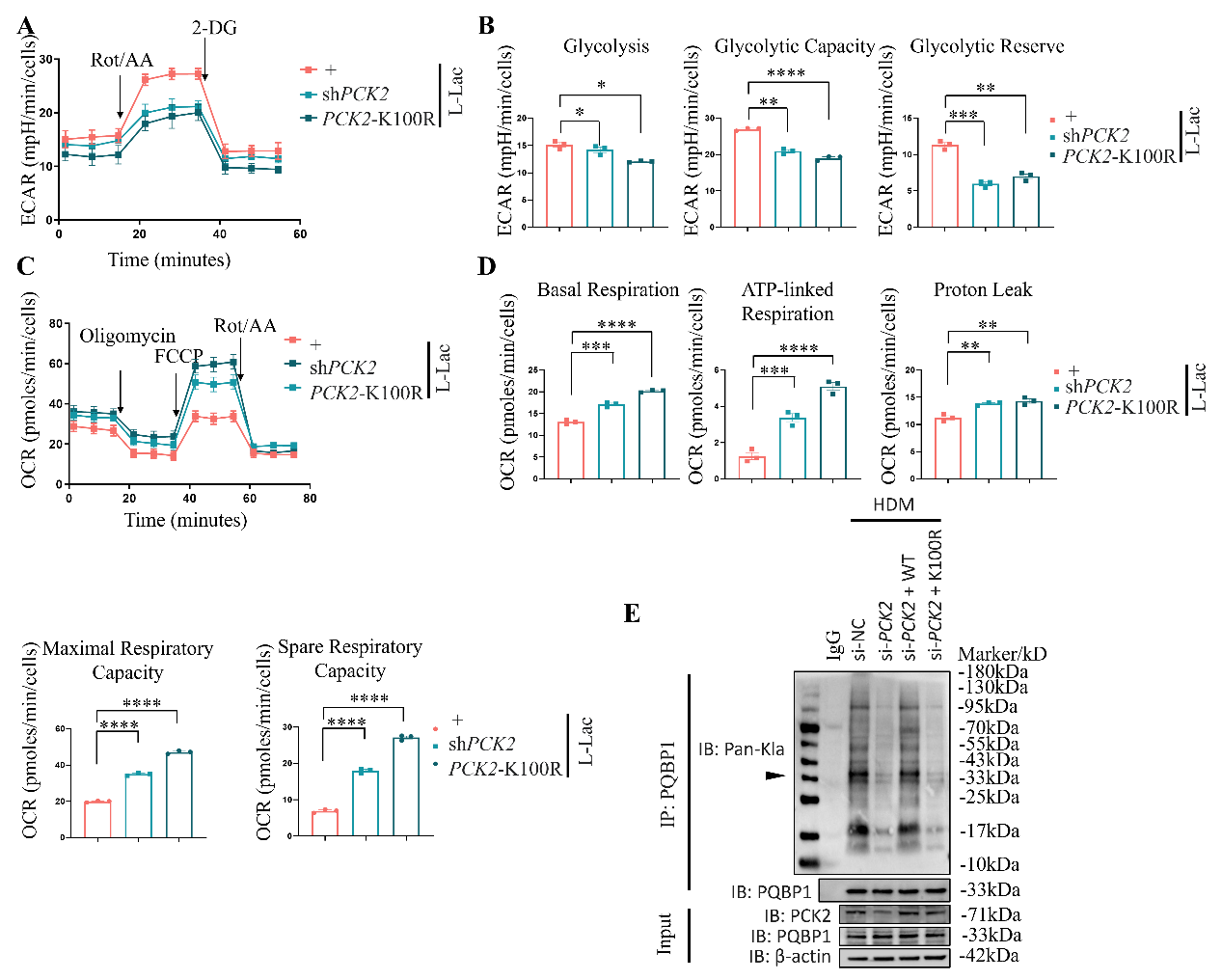


Fig. S9.

Functional validation of the PCK2-K100 site via a genetic rescue experiment. Seahorse analysis of ECAR (A, B) and OCR (C, D) in lactate-stimulated (L-Lac) cells from the Control (+), *PCK2* knockdown (sh*PCK2*), and K100R mutant (PCK2-K100R) groups. (E) Immunoprecipitation (IP) and Western blot analysis of PQBP1 lactylation (Pan-Kla) in HDM-stimulated BEAS-2B cells. Endogenous ***PCK2*** was knocked down by siRNA (si-***PCK2***), followed by reconstitution with either wild-type PCK2 (si-***PCK2*** + WT) or the K100R mutant (si-***PCK2*** + K100R). Total PQBP1 and PCK2 in the input and IP complexes were verified. Black arrowhead indicates the target lactylated PQBP1 band. Data are presented as mean ± SEM (n=3 independent experiments). Analyzed by One or two-way ANOVA. *P < 0.05, **P < 0.01, ***P < 0.001, ****P < 0.0001.


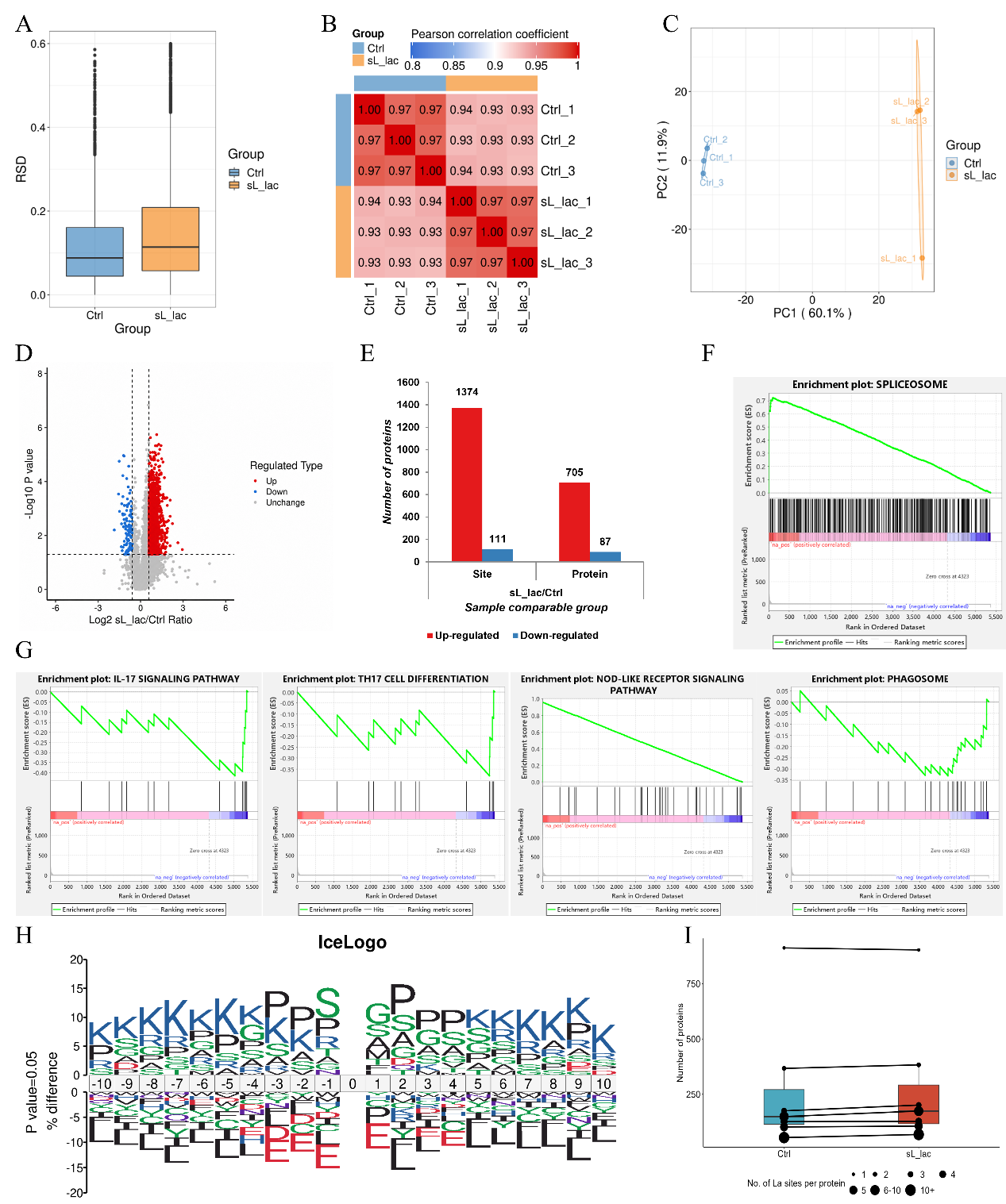


Fig. S10.

Data analysis of lactyl-proteomics. (A-C) Quality control, including RSD (A), Pearson correlation (B), and PCA (C). (D, E) Volcano plot (D) and statistical summary (E) of differentially lactylated sites/proteins. (F, G) GSEA of differentially lactylated proteins. (H, I) Motif analysis (H) and site count distribution (I) of differentially lactylated sites.


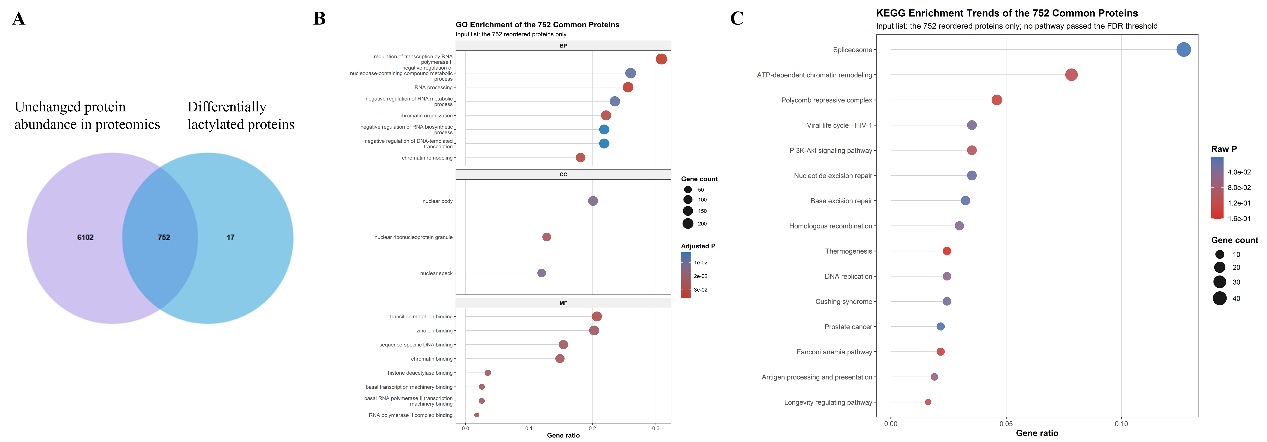


Fig. S11.

Multi-omics intersection and pathway-based hierarchical filtering identify key lactate-responsive targets. (A) Venn diagram showing the intersection between proteins with unchanged abundance in the global proteome (purple) and differentially lactylated proteins (blue). (B, C) Gene Ontology (GO) (B) and Kyoto Encyclopedia of Genes and Genomes (KEGG) (C) pathway enrichment analyses of the 752 high-confidence targets.


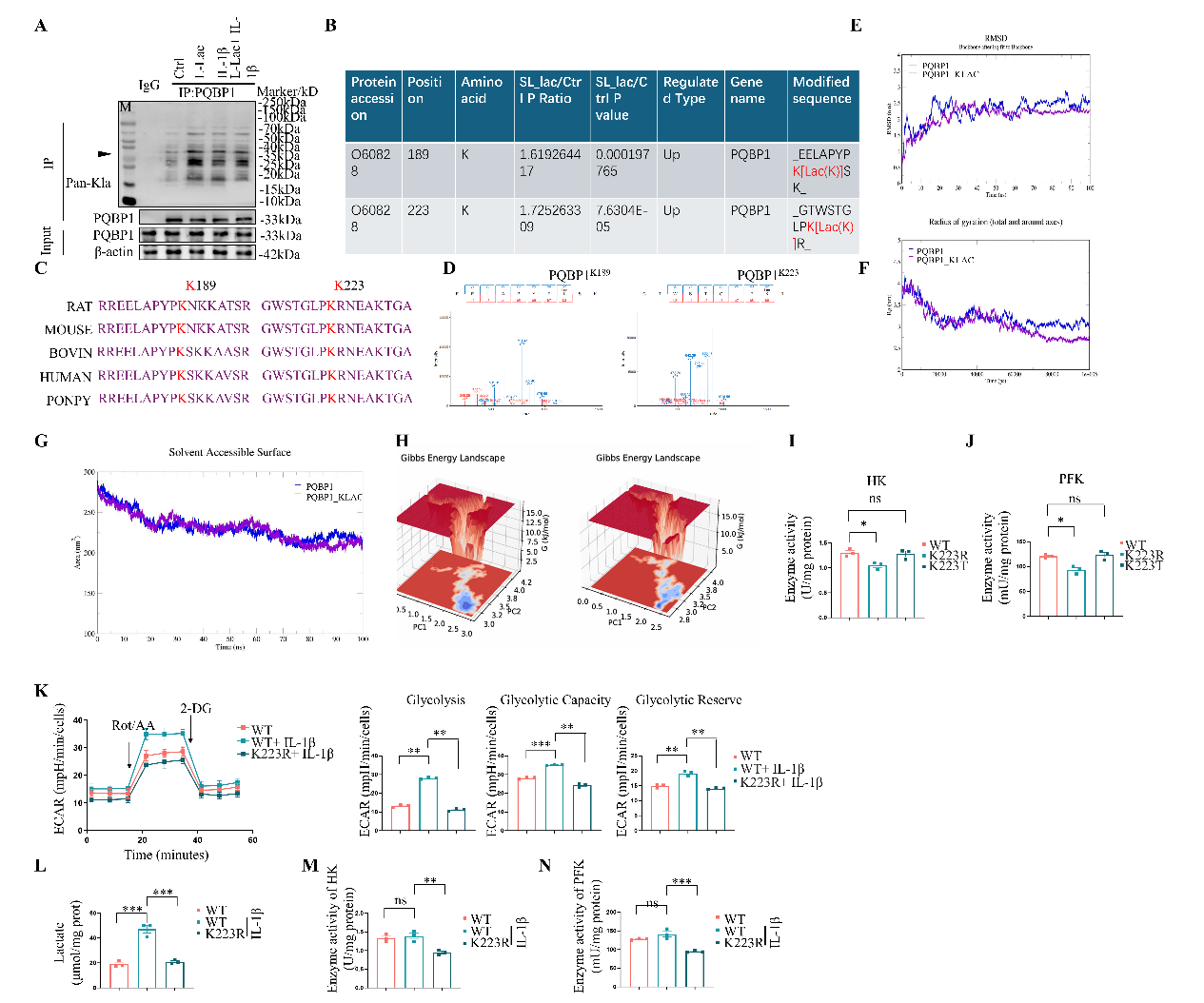


Fig. S12.

Validation and functional analysis of PQBP1 lactylation. (A) IP-Western blot analysis of PQBP1 lactylation in response to lactate and IL-1β. (B-D) Mass spectrometric identification of PQBP1 lactylation sites, including site information (B), sequence conservation (C), and MS/MS spectrum (D). (E-H) Molecular dynamics simulation analysis of the effect of K223 lactylation on PQBP1 protein conformation, including RMSD (E), Rg (F), SASA (G), and Gibbs free energy landscape (H). (I, J) Measurement of HK (I) and PFK (J) activity in cells expressing WT or mutant (K223R/T) PQBP1. (K-N) Assessment of the effect of IL-1β on ECAR (K), lactate production (L), and HK (M) and PFK (N) enzyme activity in cells expressing WT or K223R mutant PQBP1. All Western blots are representative of ≥3 independent experiments. Data are presented as mean ± SEM (n=3 independent experiments). Analyzed by one- or two-way ANOVA. *P < 0.05, **P < 0.01, ***P < 0.001, ****P < 0.0001; ns, not significant.


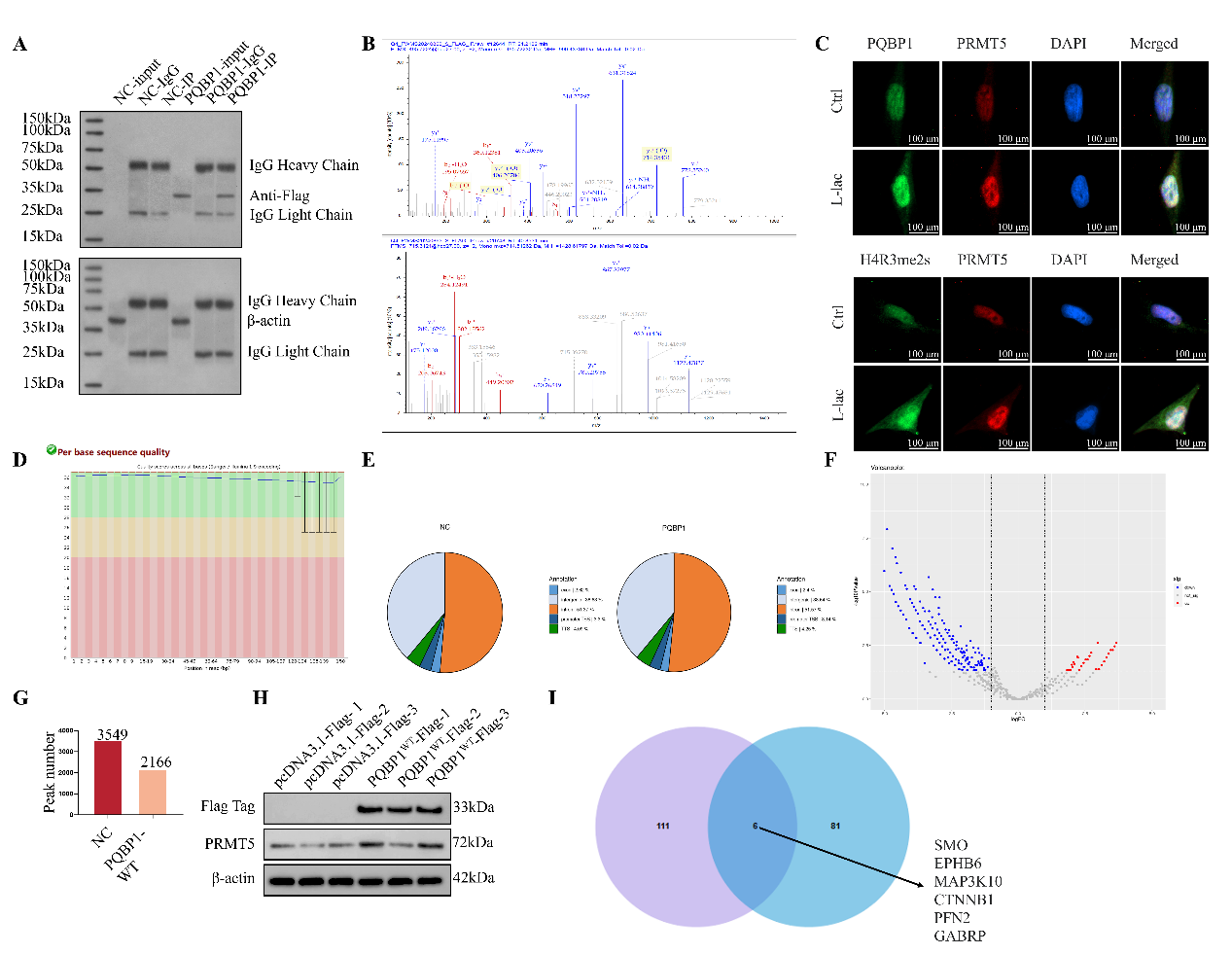


Fig. S13.

PQBP1-PRMT5 interaction and ChIP-seq enrichment analysis. (A, B) SDS-PAGE (A) and mass spectrometry signal quantification of PRMT5 and WDR77 (B) of IP-MS samples. (C) Immunofluorescence showing the co-localization of PQBP1 and PRMT5 (top), and H4R3me2s and PRMT5 (bottom) in BEAS-2B cells treated with or without Lactate (L-lac). Scale bar, 100 μm. (D, E) Quality control of H4R3me2s ChIP-seq data, including base quality assessment (D) and peak distribution across the genome (E). (F, G) Volcano plot (F) and statistical summary (G) of differential ChIP-seq peaks. (H) Effect of PQBP1 overexpression on endogenous PRMT5 protein levels. (I) Intersection analysis of ChIP-seq target genes and KEGG pathway genes. All Western blots are representative of ≥3 independent experiments.


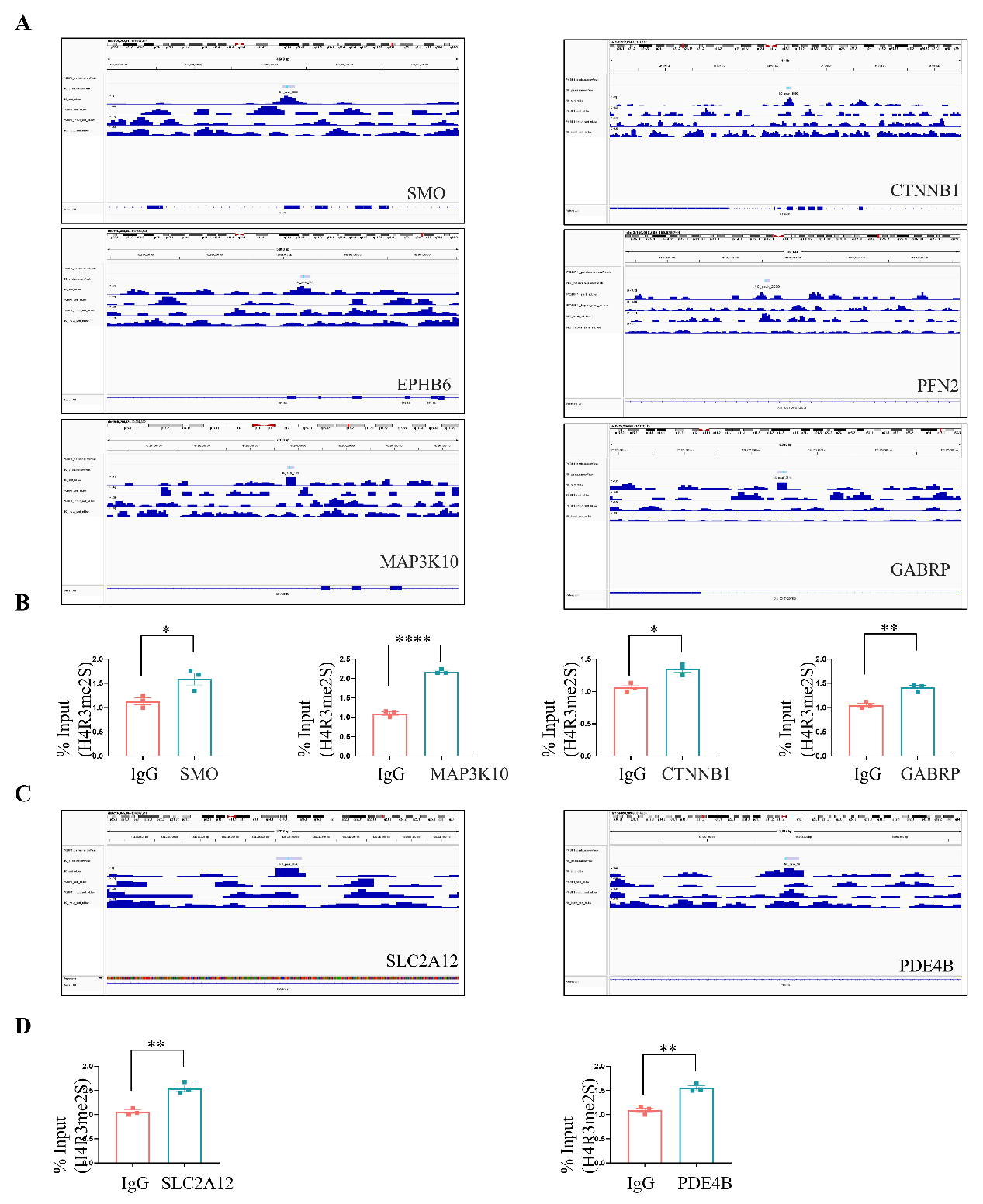


Fig. S14.

ChIP-seq enrichment analysis. (A, C) ChIP-seq signal tracks at promoter regions of representative target genes. (B, D) Potential ChIP-qPCR validation for the corresponding loci. Analyzed by Student's t-test. Data are presented as mean ± SEM (n=3 independent experiments). *P < 0.05, **P < 0.01, ****P < 0.0001; ns, not significant.


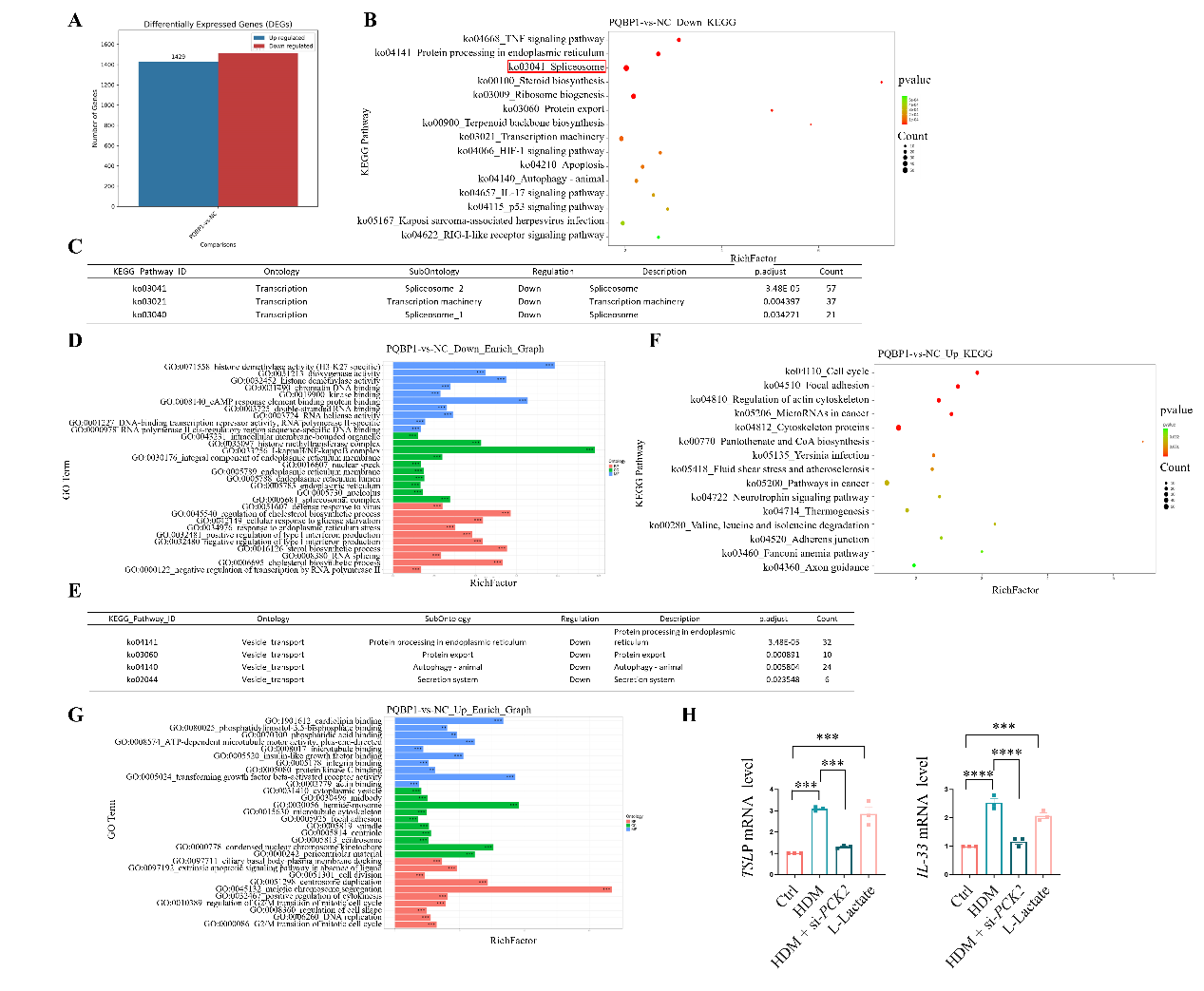


Fig. S15.

RNA-sequencing analysis of the global transcriptional impact of PQBP1 overexpression. (A) Statistical summary of the number of differentially expressed genes (DEGs). (B-E) KEGG and GO pathway enrichment analyses for downregulated genes, shown as bubble plots (B), detailed pathway lists (C, E), and bar charts (D). (F, G) KEGG (F) and GO (G) pathway enrichment analyses for upregulated genes. (H) RT-qPCR validation of mRNA levels for classic asthma-related alarmins *TSLP* (left) and *IL-33* (right) in BEAS-2B cells under four conditions: Control (Ctrl), HDM stimulation, HDM stimulation with PCK2 knockdown (HDM + si-PCK2), and direct L-Lactate stimulation. Data are presented as mean ± SEM (n=3 independent biological replicates). Significant differences were analyzed by one-way ANOVA. ***P < 0.001, ****P < 0.0001.


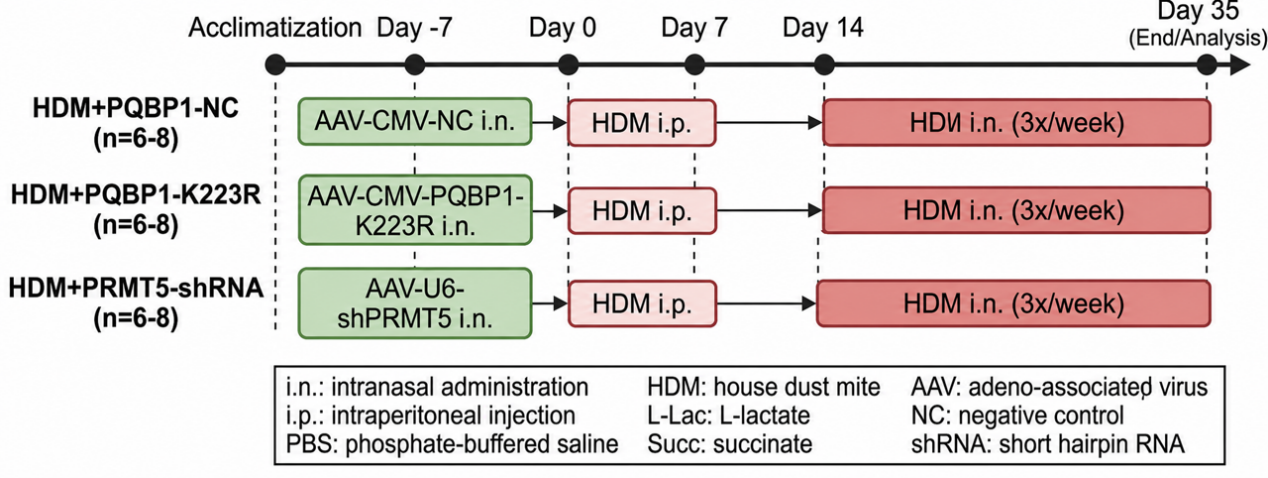


Fig. S16.

Schematic diagram of the experimental design for AAV-mediated gene intervention in the HDM-induced asthma model.


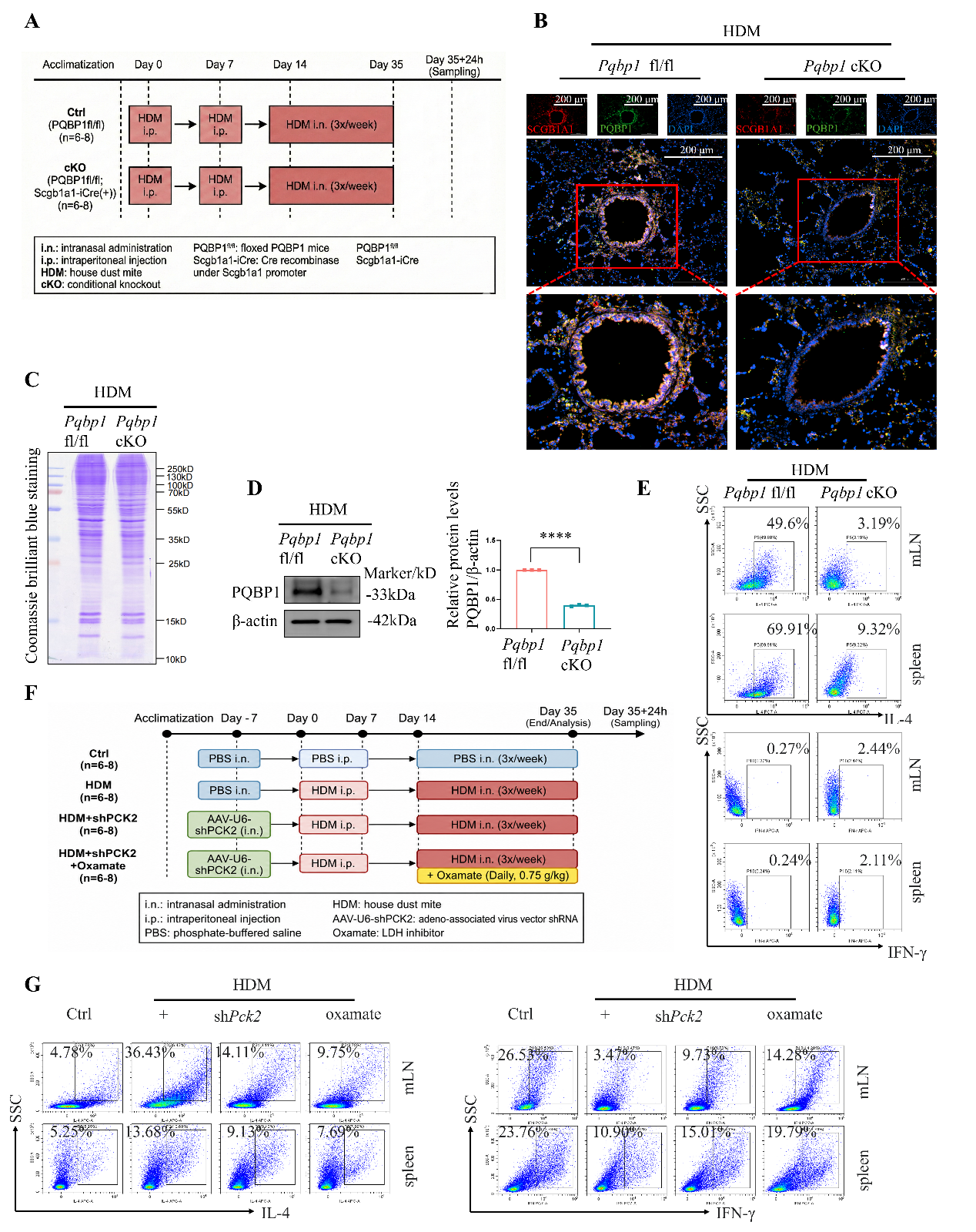


Fig. S17.

Validation of the PQBP1 conditional knockout (cKO) mouse model. (A) Protocol for HDM-induced asthma in airway epithelial-specific *Pqbp1* conditional knockout mice. (B) Immunofluorescence co-staining to assess the knockout efficiency of PQBP1 in the airway epithelium of cKO mice. Scale bar, 200 μm. (C, D) Coomassie blue staining (C) and Western blot (D) to evaluate PQBP1 protein levels and sample loading in lung tissues from cKO mice. (E) Representative flow cytometry plots of Th1/Th2 cells (related to the quantification in Figure 8D). (F) Schematic illustration of the experimental design for evaluating *Pck2* knockdown and lactate inhibition *in vivo*. C57BL/6 mice were randomized into four groups: Ctrl, HDM, HDM+sh*Pck2*, and HDM+sh*Pck2*+Oxamate. (n = 6–8 per group). (G) Representative flow cytometry plots of Th1/Th2 cells (related to the quantification in Figure 8K). All Western blots are representative of ≥3 independent experiments. Data are presented as mean ± SEM (n=6-8 per group). Analyzed by Student's t-test. ****P < 0.0001.


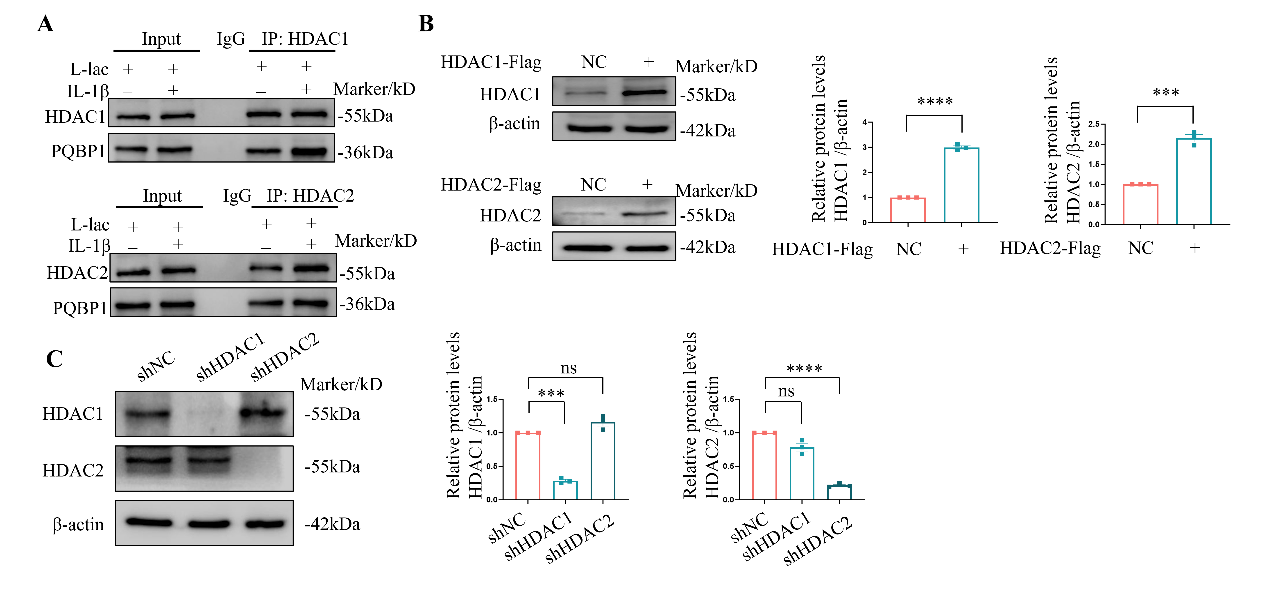


Fig. S18.

PQBP1 interaction with HDACs and validation of related genetic interventions. (A) Endogenous Co-IP showing the effect of IL-1β stimulation on the interaction between PQBP1 and HDAC1/2. (B, C) Western blot validation of the efficiency of HDAC1/2 overexpression (B) and shRNA-mediated knockdown (C). All Western blots are representative of ≥3 independent experiments. Data are presented as mean ± SEM (n=3 independent experiments). Analyzed by Student's t-test or one-way ANOVA. ***P < 0.001, ****P < 0.0001; ns, not significant.


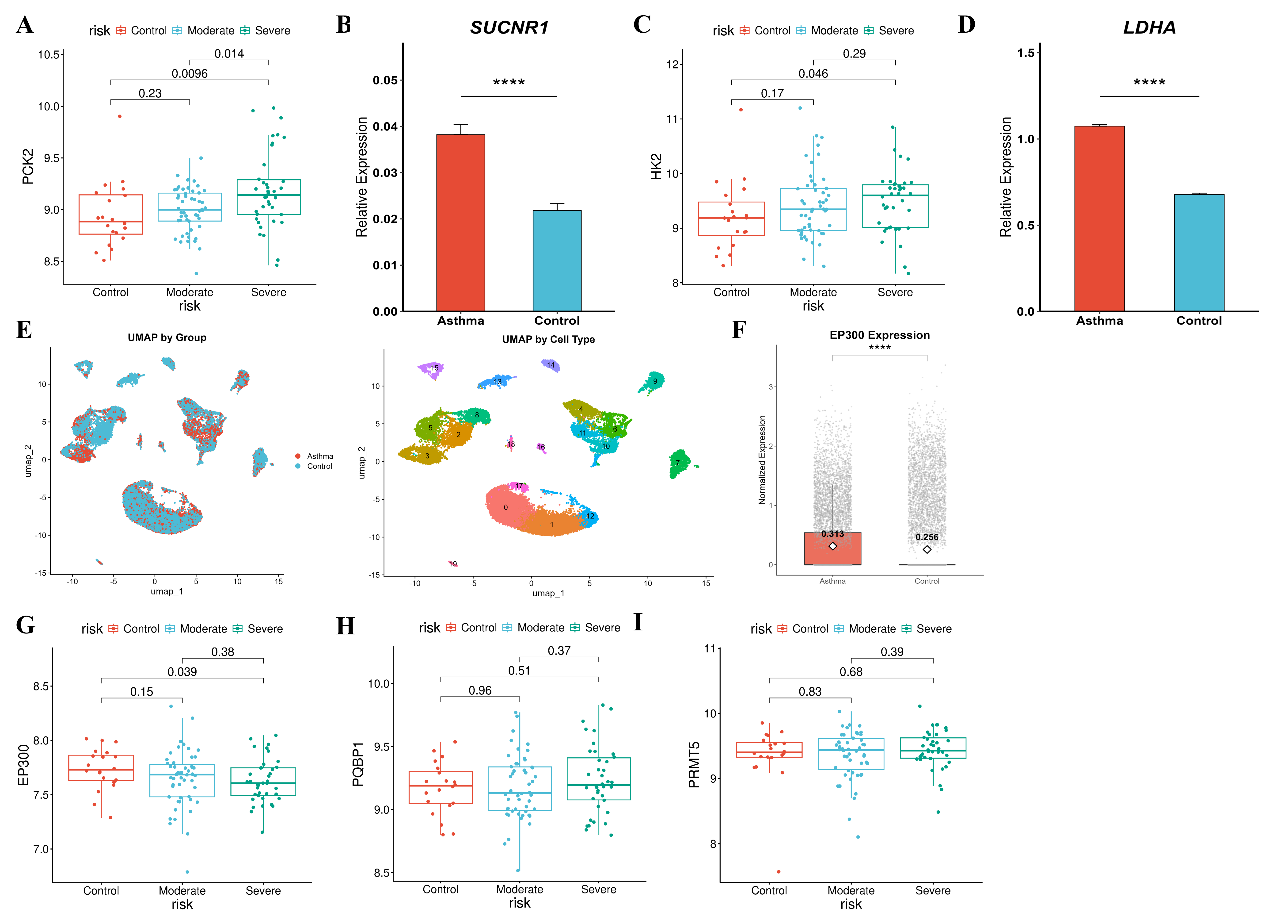


Fig. S19.

Multi-omics integration verifies the metabolic and epigenetic basis of the PCK2-Lactate-PQBP1 axis in clinical asthma. (A, C) Analysis of the bulk transcriptomic dataset (GSE43696). Box plots showing significant upregulation of the metabolic enzymes *PCK2* (A) and *HK2* (C) in severe asthma patients compared to controls. (B, D) Analysis of the single-cell RNA-seq dataset (GSE193816). Bar plots showing the specific upregulation of the succinate receptor *SUCNR1* (B) and the lactate-generating enzyme *LDHA* (D) in asthmatic airway epithelial cells. (E) UMAP visualization of bronchial epithelial cells (GSE193816) colored by group (Left) and cell type (Right). (F) Violin plot showing *EP300* upregulation in asthmatic epithelial cells (GSE193816). (G-H) Expression of downstream effectors in the bulk dataset (GSE43696). Box plots show that the mRNA levels of *PQBP1* (G) and *PRMT5* (H). Data are analyzed using unpaired t-test or Wilcoxon rank-sum test. *P < 0.05, **P < 0.01, ***P < 0.001, ****P < 0.0001; ns, not significant.


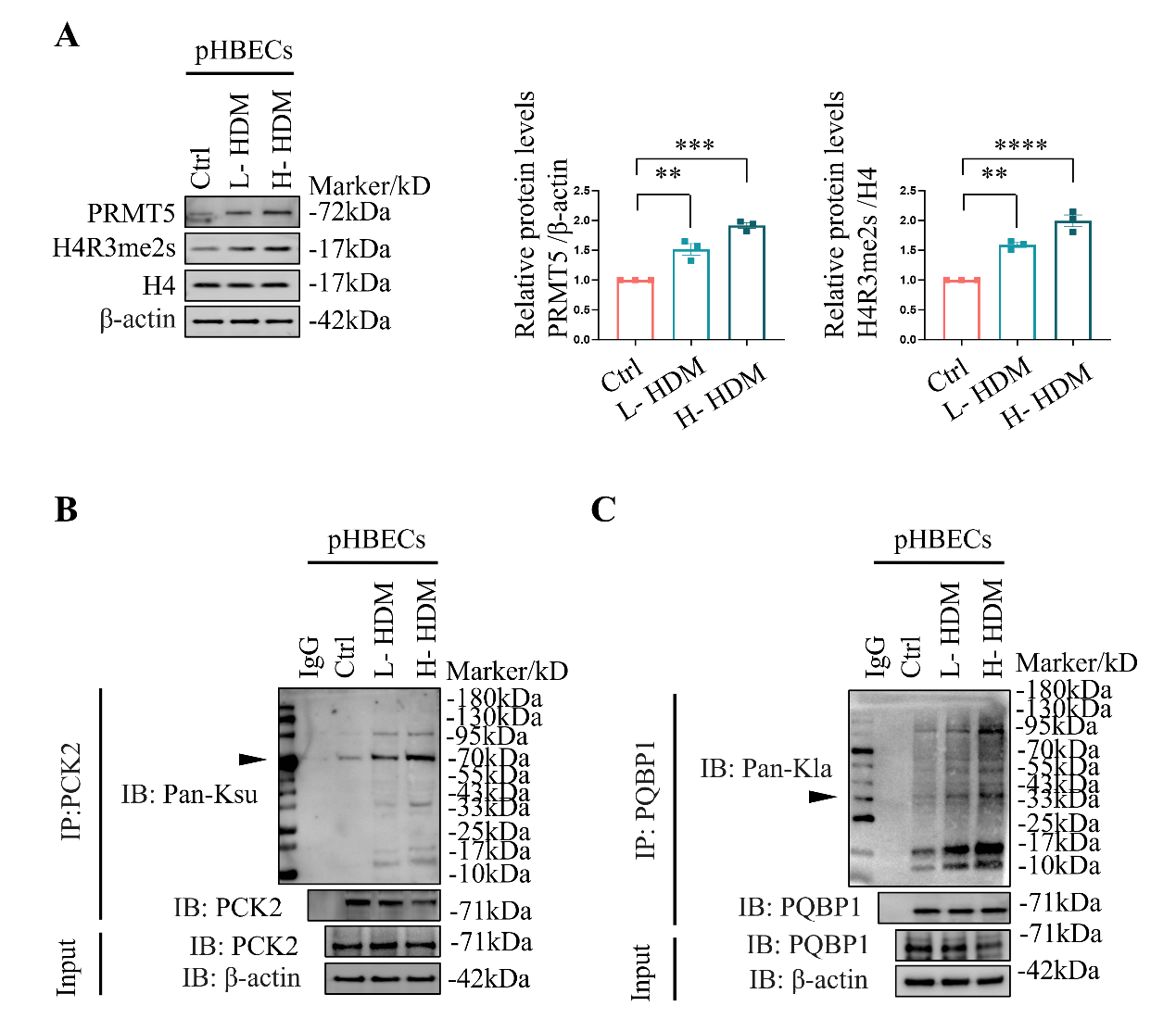


Fig. S20.

Validation of the PCK2/PQBP1/PRMT5 axis in pHBECs across an HDM-induced severity gradient. (A) Western blot analysis and quantification of PRMT5 and H4R3me2s protein levels in pHBECs treated with vehicle (Ctrl), low-dose HDM (L-HDM, 10 μg/mL), or high-dose HDM (H-HDM, 50 μg/mL) for 24 h. Histone H4 and β-actin served as loading controls. (B, C) Immunoprecipitation (IP) and Western blot analysis of PCK2 succinylation (Pan-Ksu) (B) and PQBP1 lactylation (Pan-Kla) (C) in pHBECs exposed to the indicated HDM gradient. Total PCK2 and PQBP1 in the input and IP complexes were verified. Black arrowheads indicate the corresponding target modification bands. All Western blots are representative of ≥3 independent experiments. Data are presented as mean ± SEM (n=3 independent experiments). Analyzed by one-way ANOVA. **P < 0.01, ***P < 0.001, ****P < 0.0001.
