# Supplementary figures and images for: PCK2-Mediated PQBP1 Lactylation Promotes Asthmatic Inflammation through PRMT5 Inhibition
Source: Research (Wash D C). 2026 Jun 19;9:1321. doi: 10.34133/research.1321 (PMC13280573; doi:10.34133/research.1321)

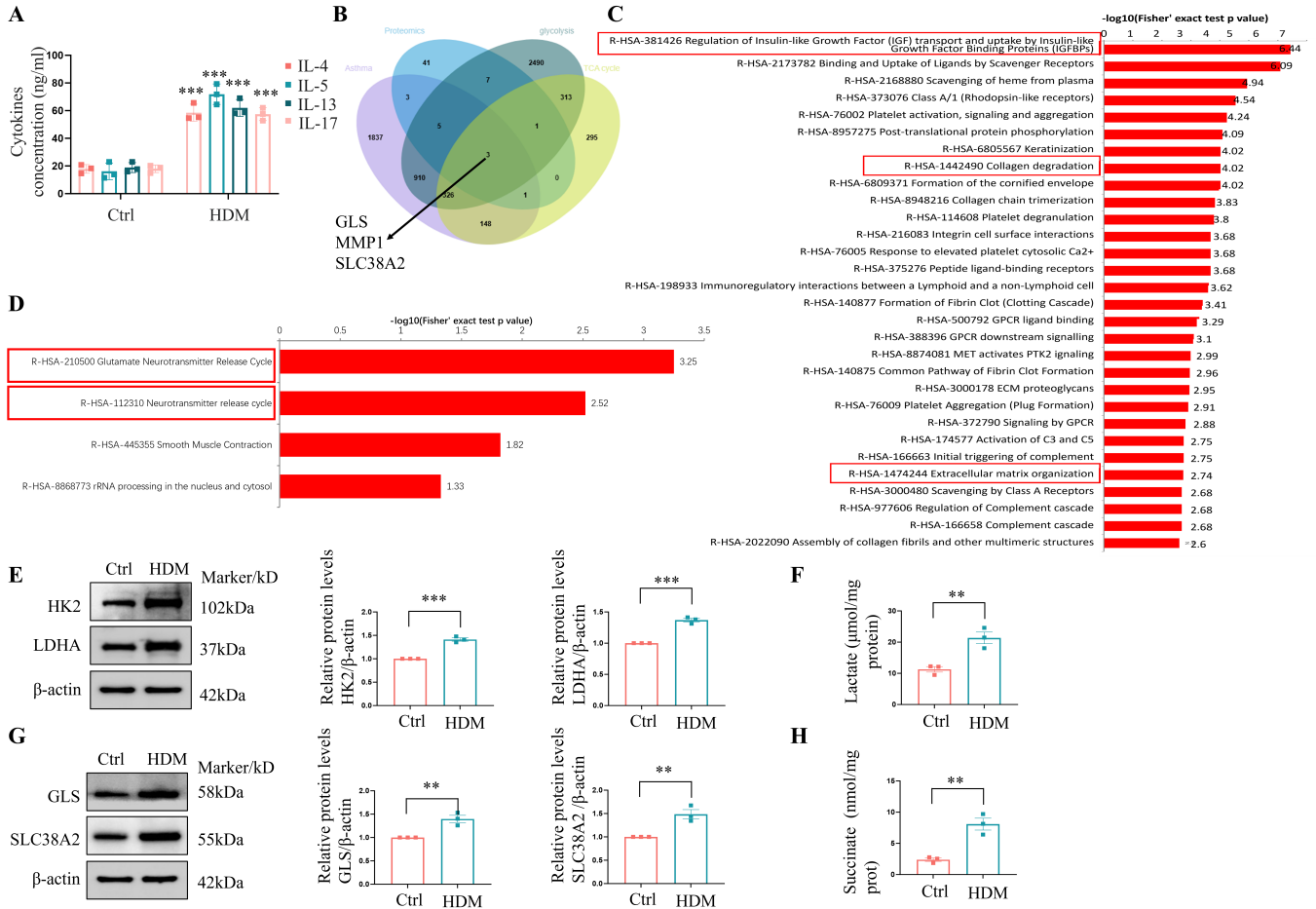

Supplement: Supplementary 1 — Figs. S1 to S20 Tables S1 and S2 [file research.1321.f1.zip › Figure S1.pdf]

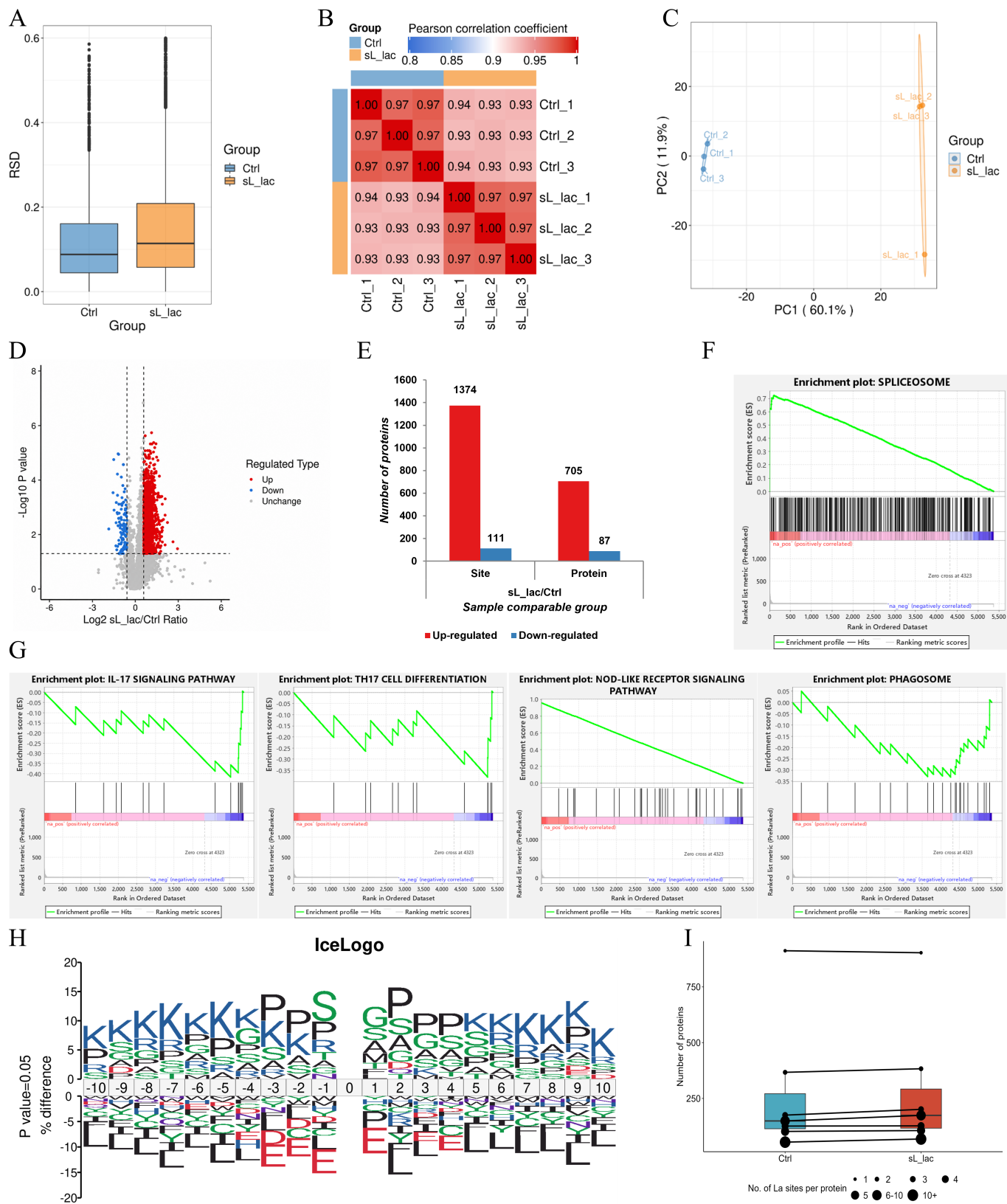

Supplement: Supplementary 1 — Figs. S1 to S20 Tables S1 and S2 [file research.1321.f1.zip › Figure S10.pdf]

A

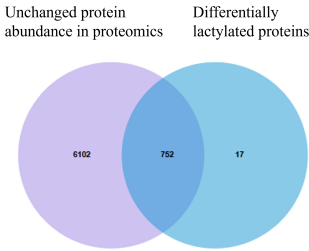

B

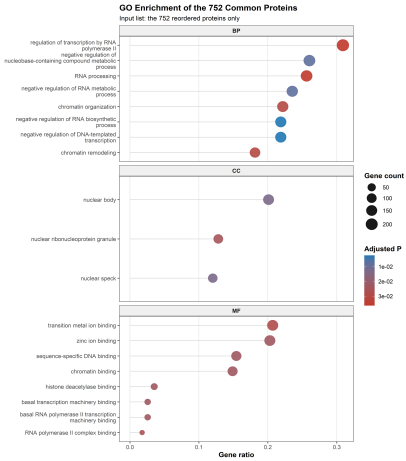

C

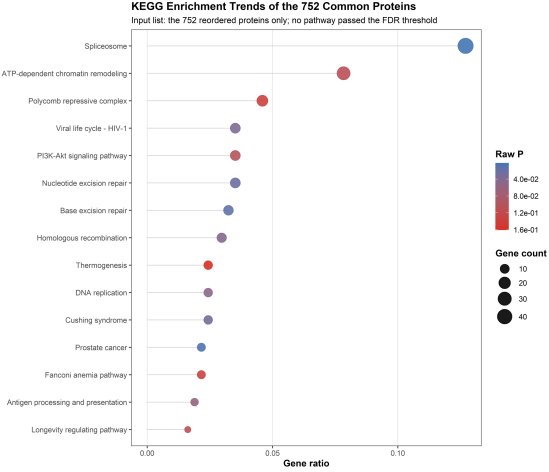

Supplement: Supplementary 1 — Figs. S1 to S20 Tables S1 and S2 [file research.1321.f1.zip › Figure S11.pdf]

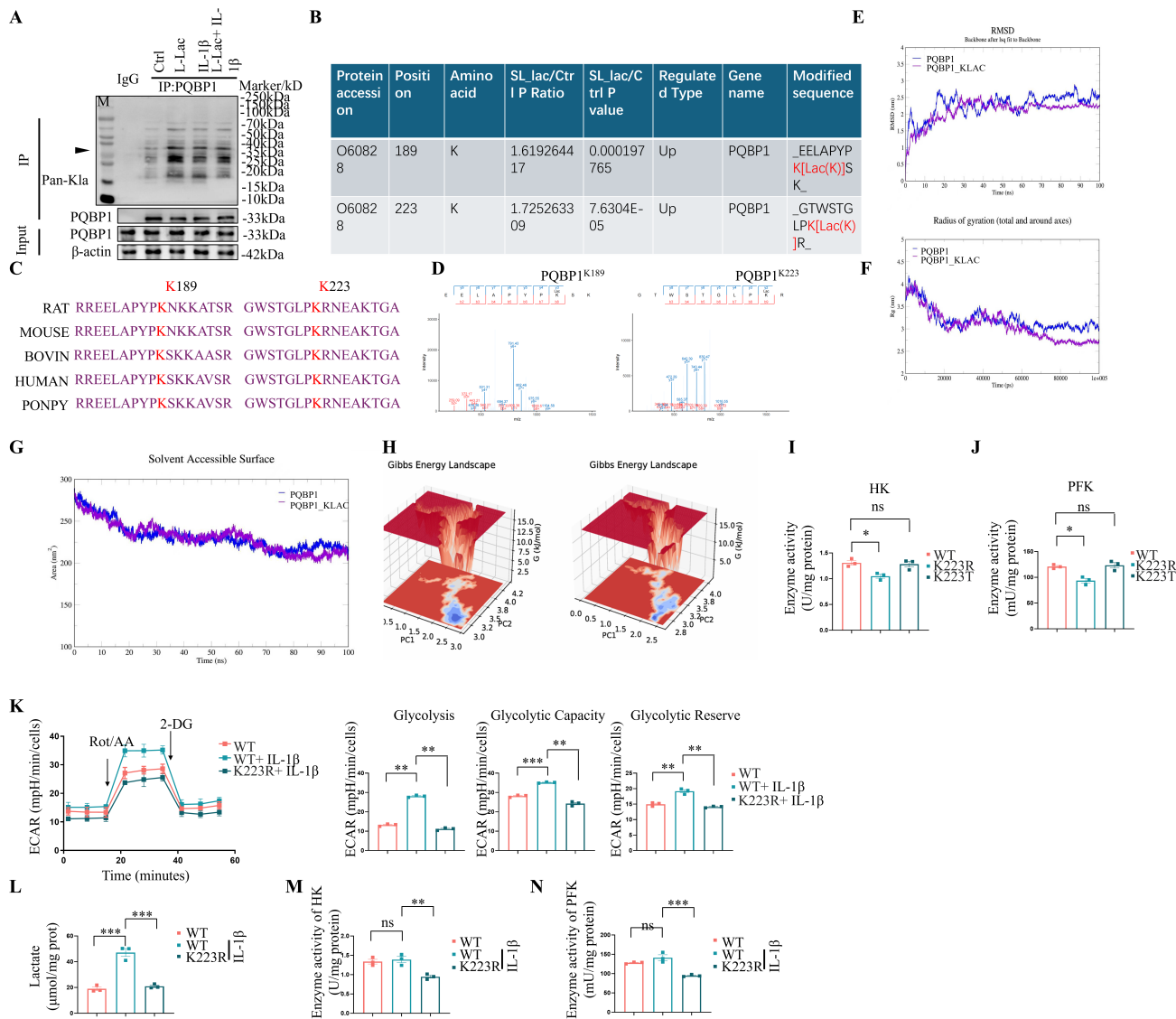

Supplement: Supplementary 1 — Figs. S1 to S20 Tables S1 and S2 [file research.1321.f1.zip › Figure S12.pdf]

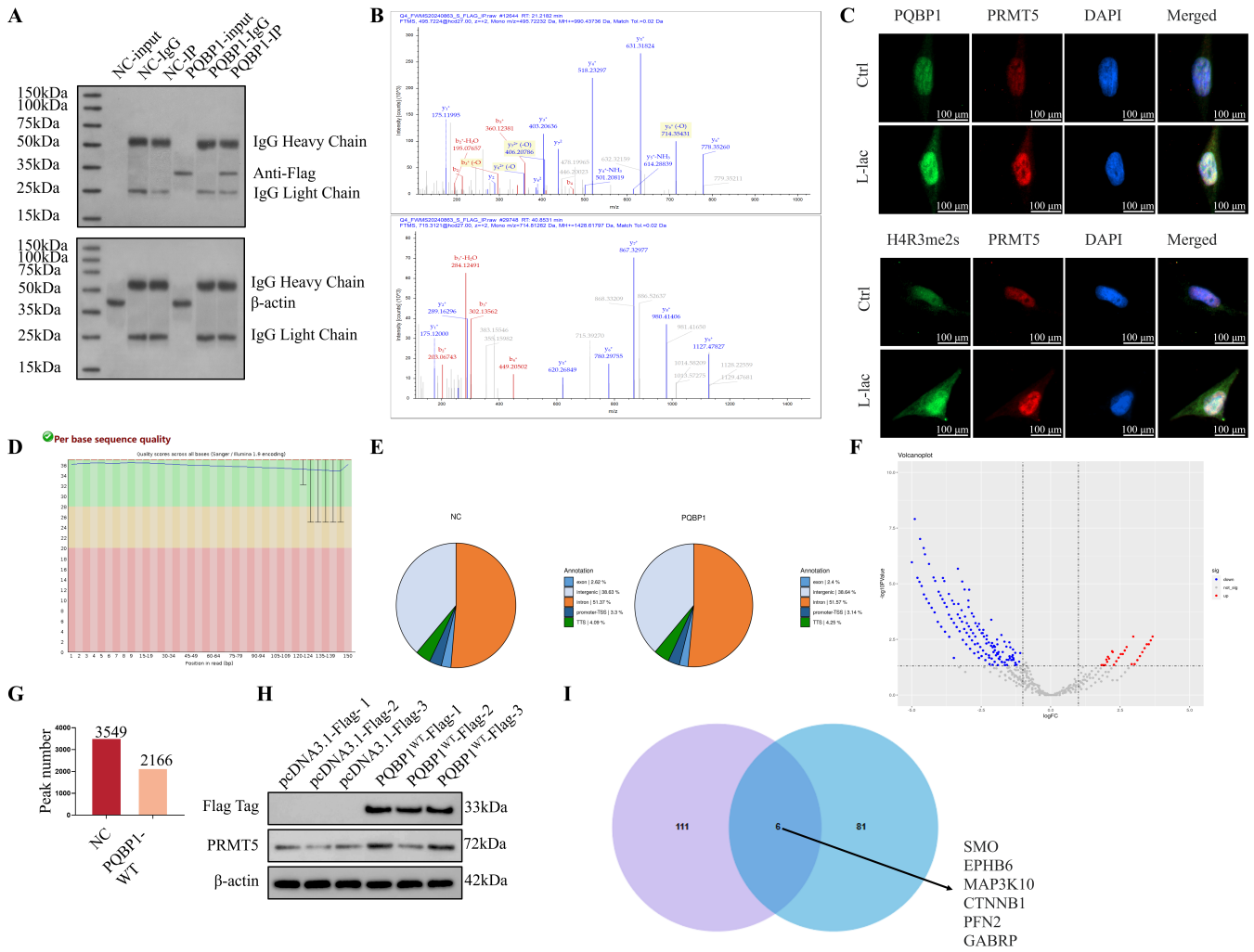

Supplement: Supplementary 1 — Figs. S1 to S20 Tables S1 and S2 [file research.1321.f1.zip › Figure S13.pdf]

A

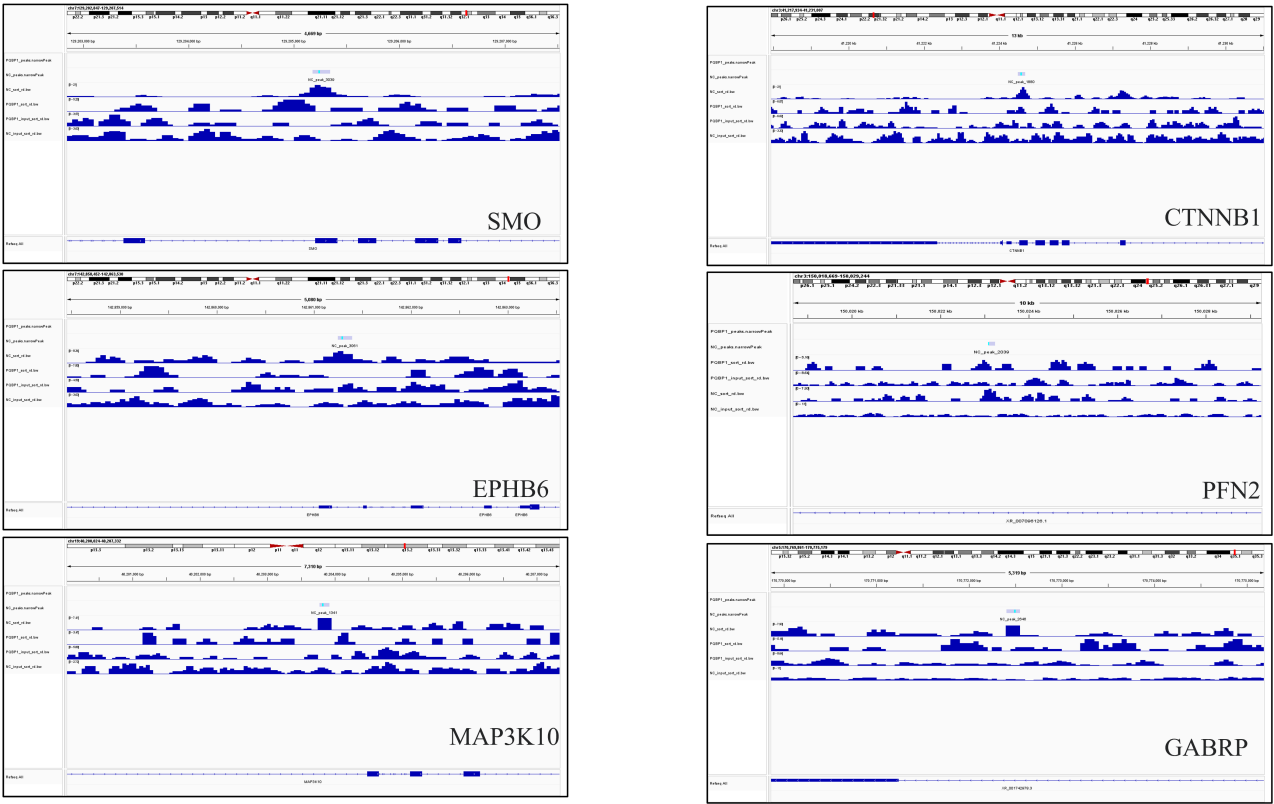

B

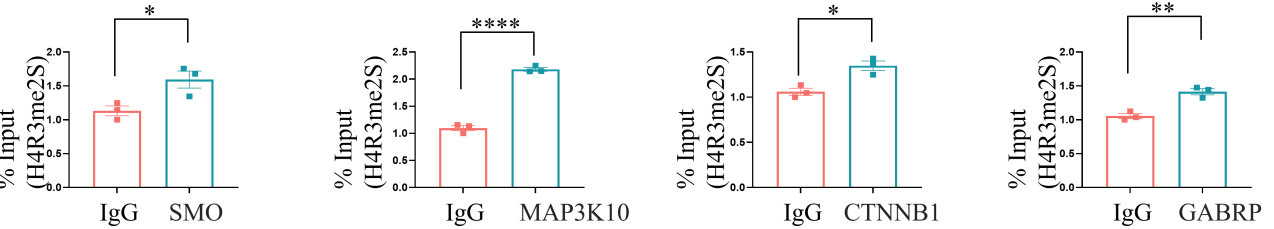

C

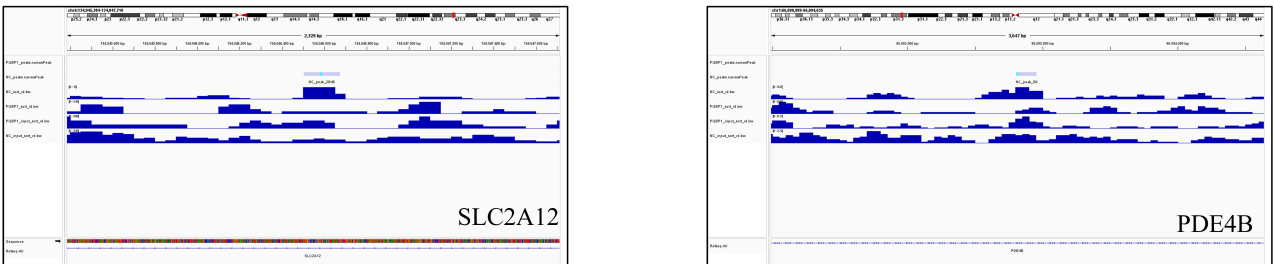

D

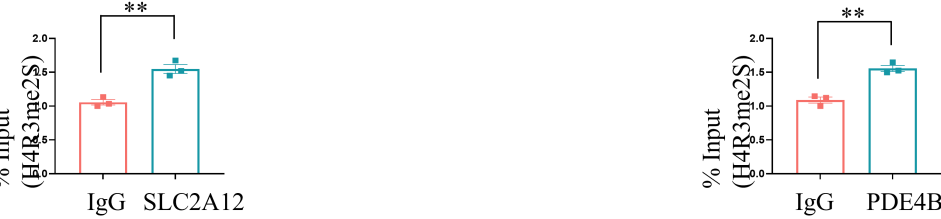

Supplement: Supplementary 1 — Figs. S1 to S20 Tables S1 and S2 [file research.1321.f1.zip › Figure S14(1).pdf]

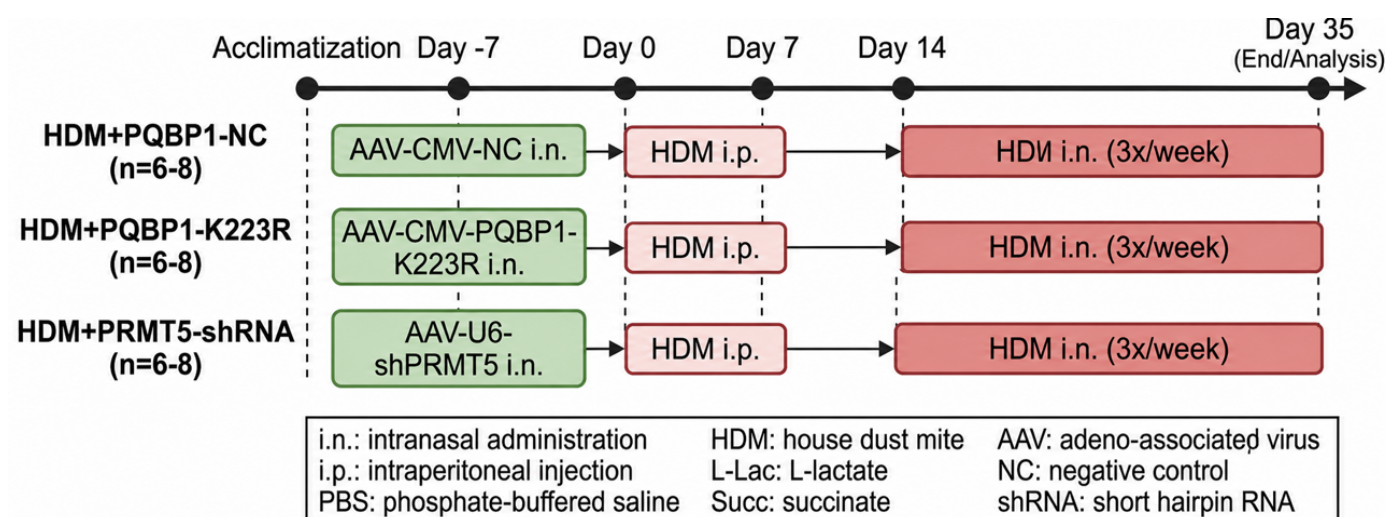

Supplement: Supplementary 1 — Figs. S1 to S20 Tables S1 and S2 [file research.1321.f1.zip › Figure S16.pdf]

**A**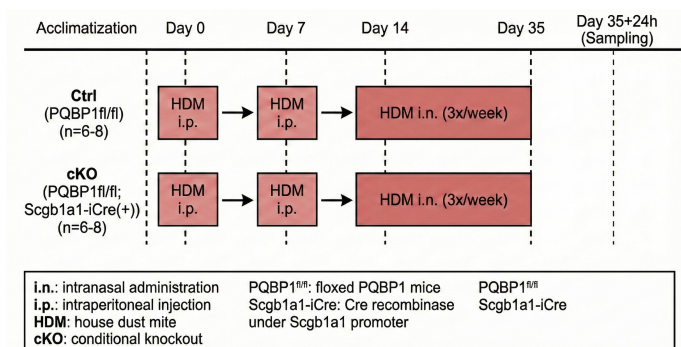**B**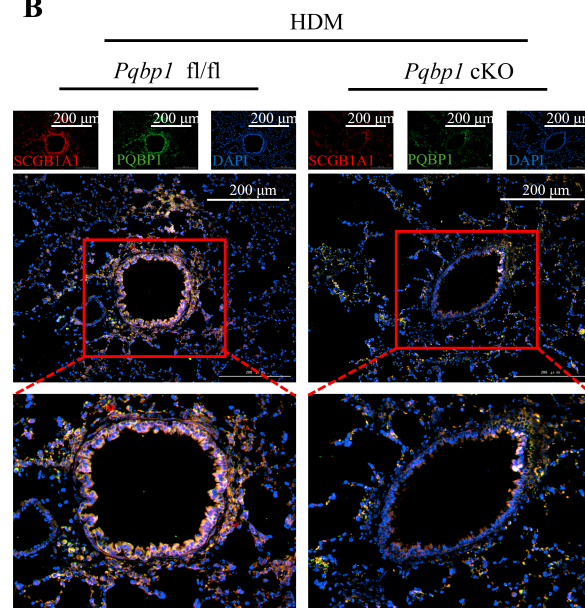**C**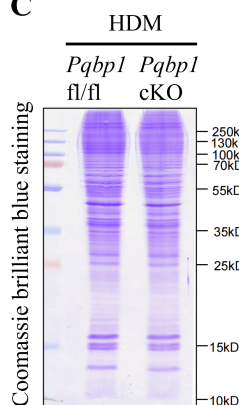**D**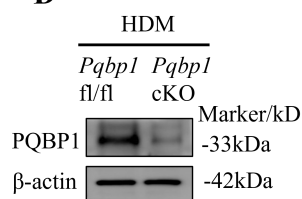**E**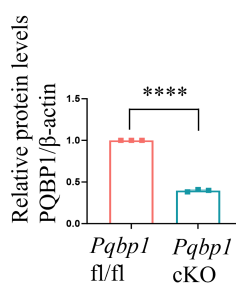**F**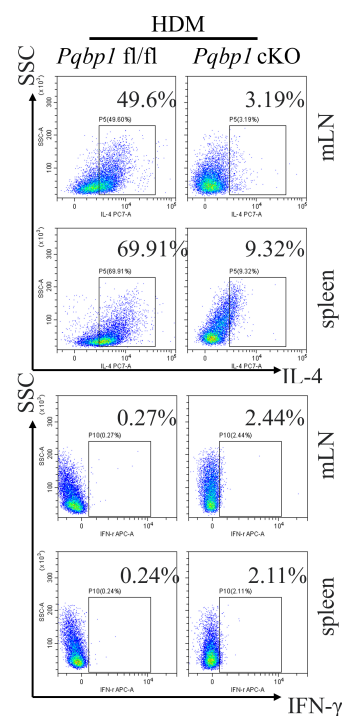**G**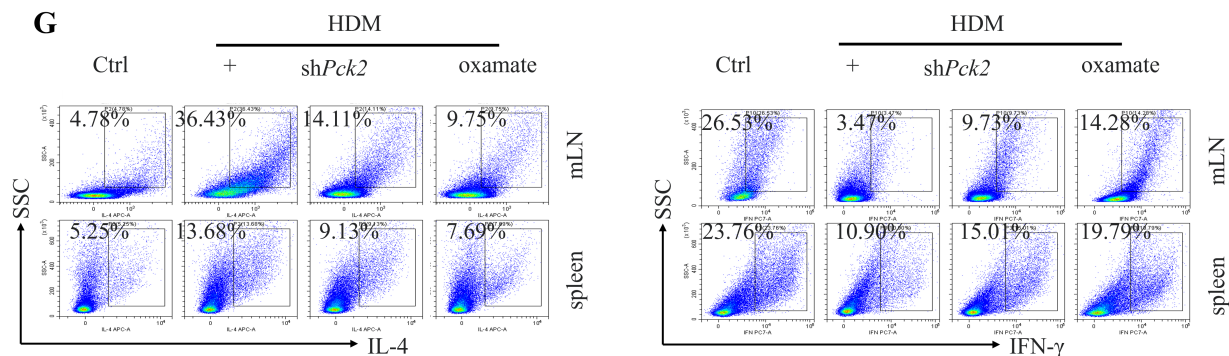

Supplement: Supplementary 1 — Figs. S1 to S20 Tables S1 and S2 [file research.1321.f1.zip › Figure S17(1).pdf]

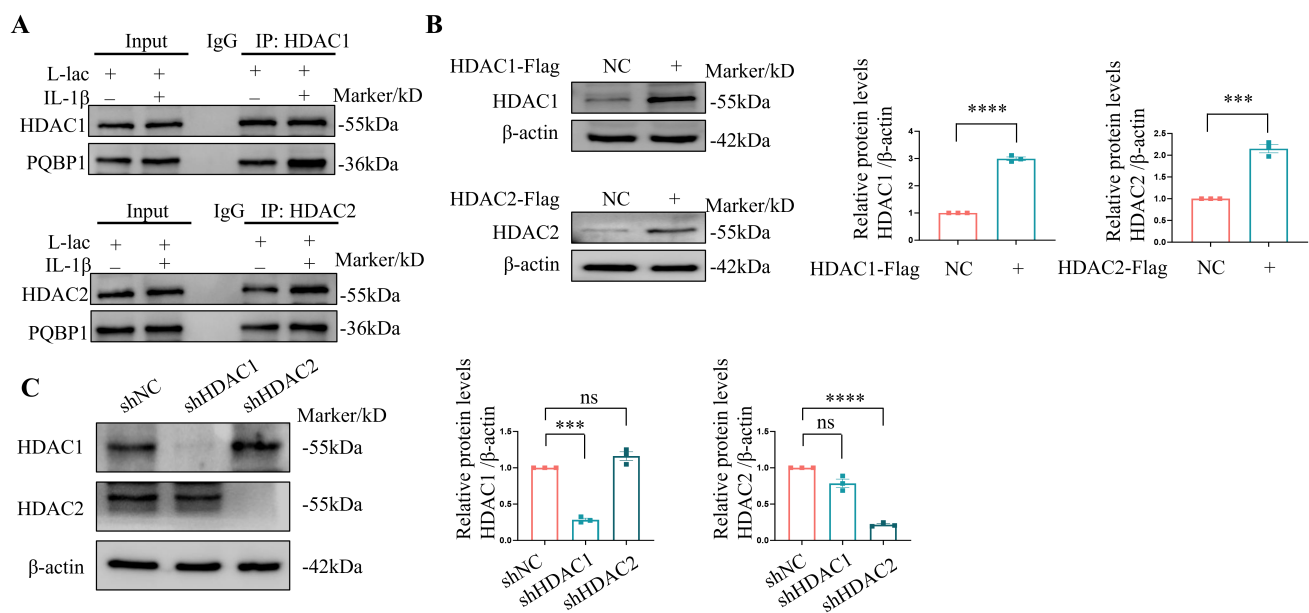

Supplement: Supplementary 1 — Figs. S1 to S20 Tables S1 and S2 [file research.1321.f1.zip › Figure S18.pdf]

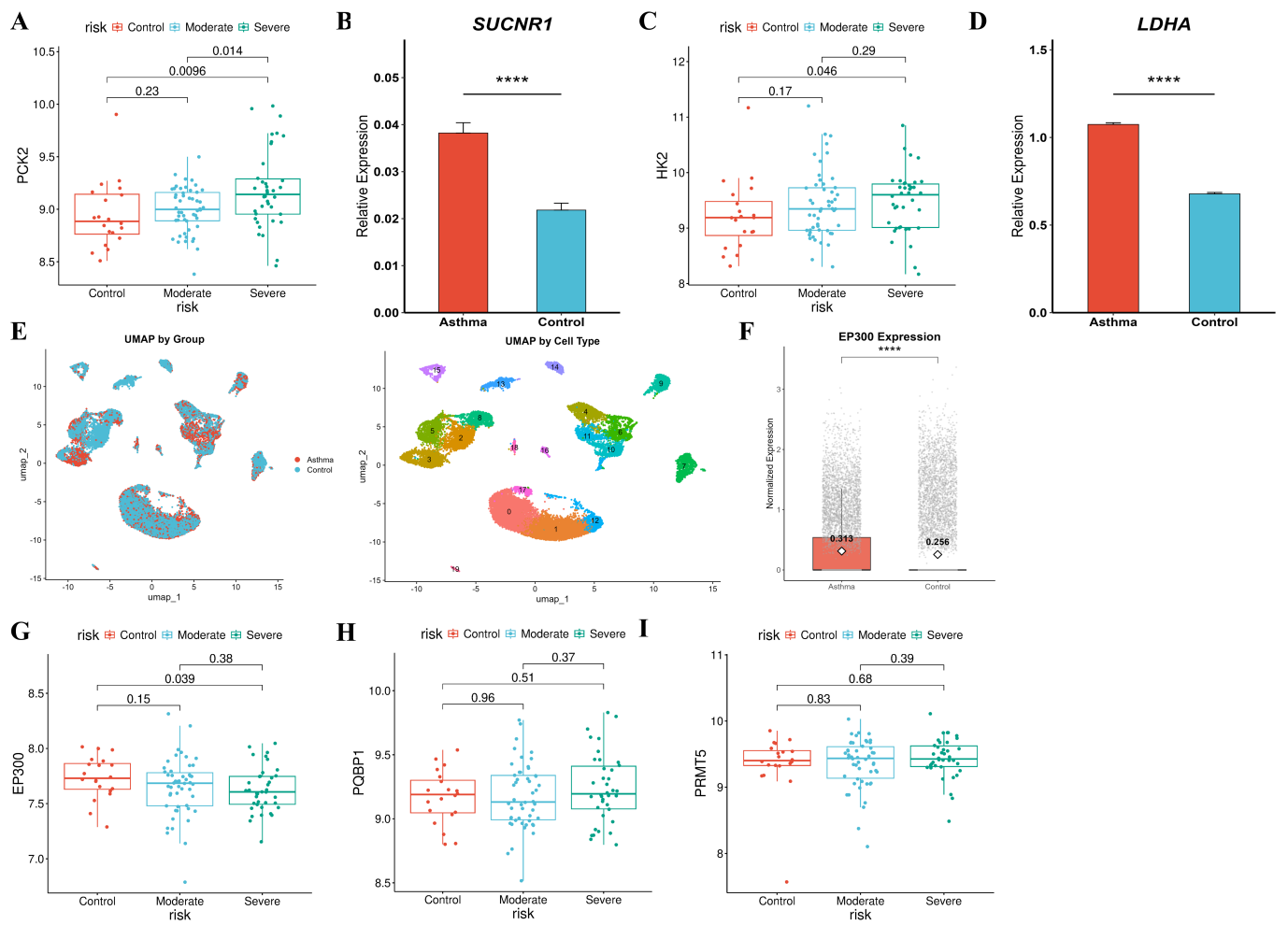

Supplement: Supplementary 1 — Figs. S1 to S20 Tables S1 and S2 [file research.1321.f1.zip › Figure S19.pdf]

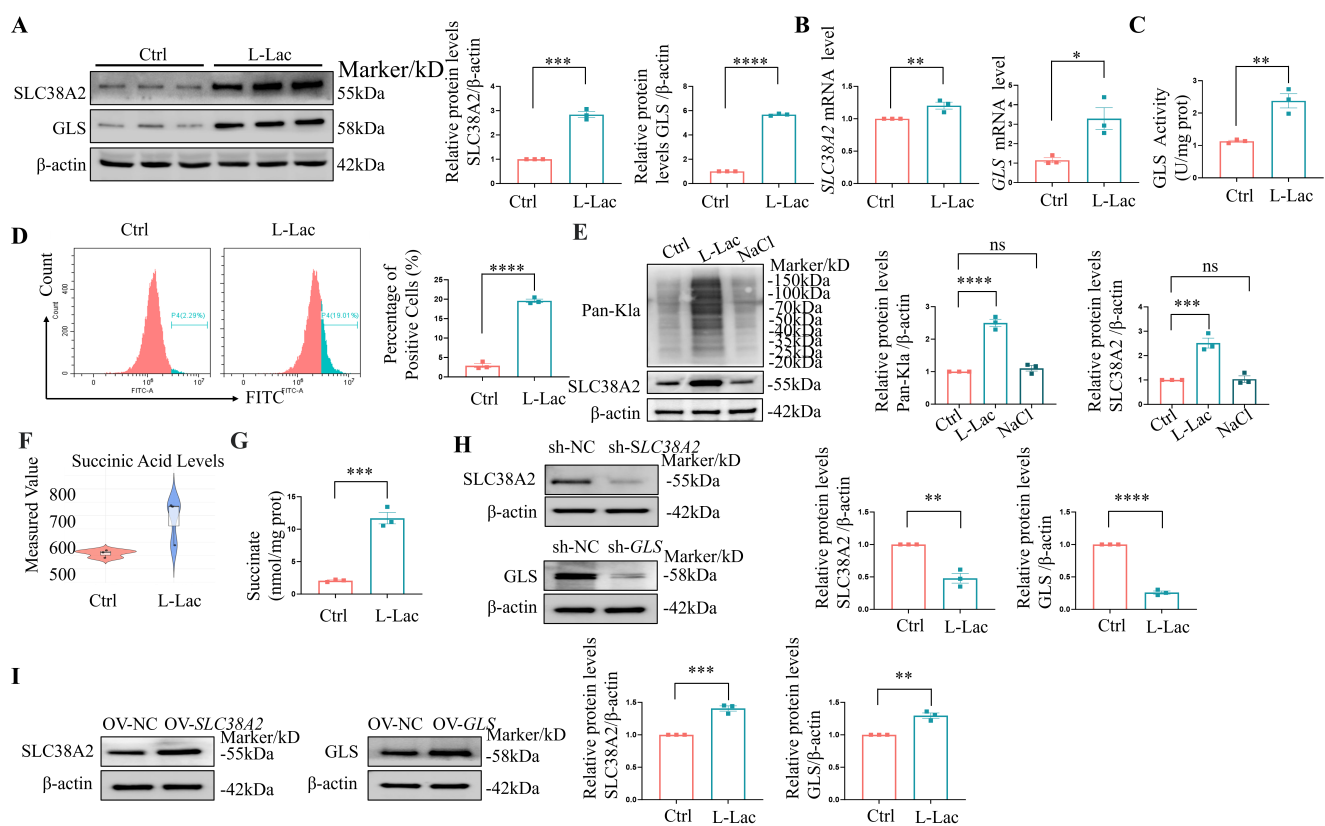

Supplement: Supplementary 1 — Figs. S1 to S20 Tables S1 and S2 [file research.1321.f1.zip › Figure S2(1).pdf]

**A**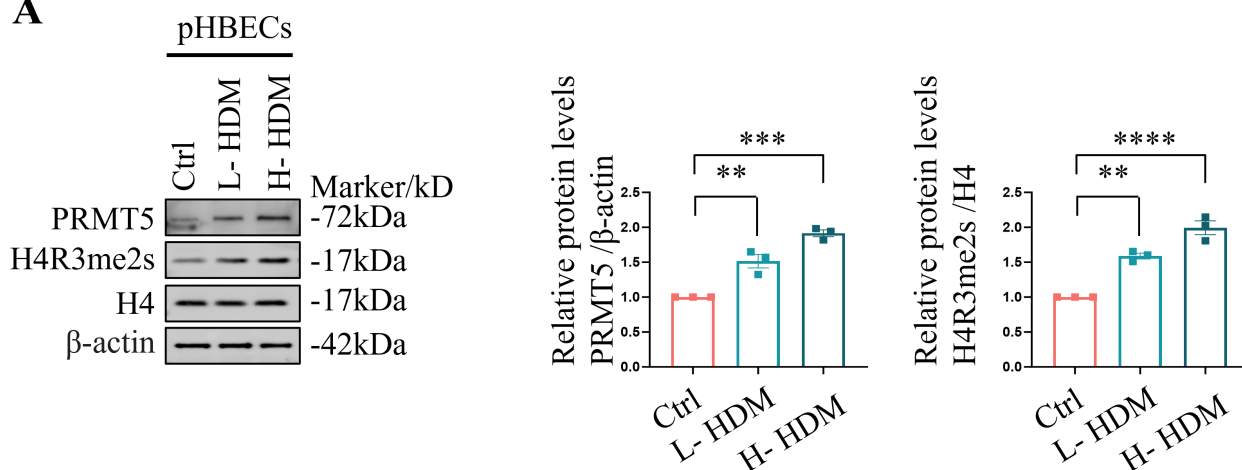**B**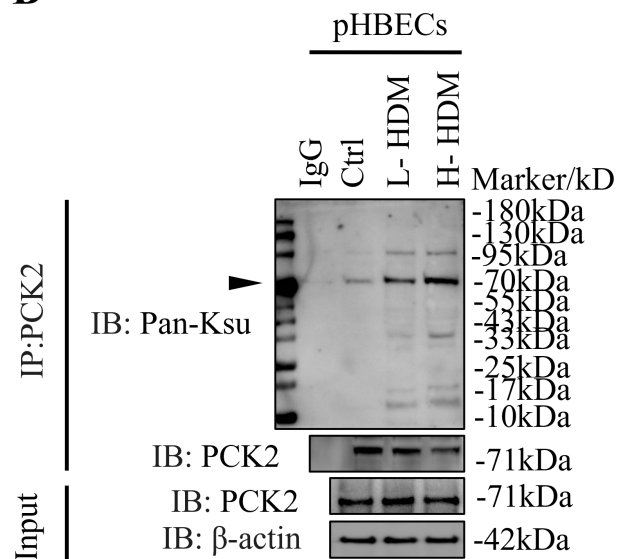**C**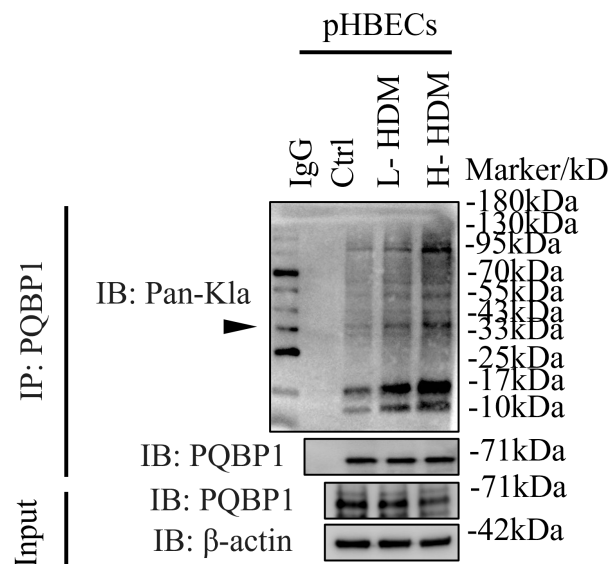

Supplement: Supplementary 1 — Figs. S1 to S20 Tables S1 and S2 [file research.1321.f1.zip › Figure S20.pdf]

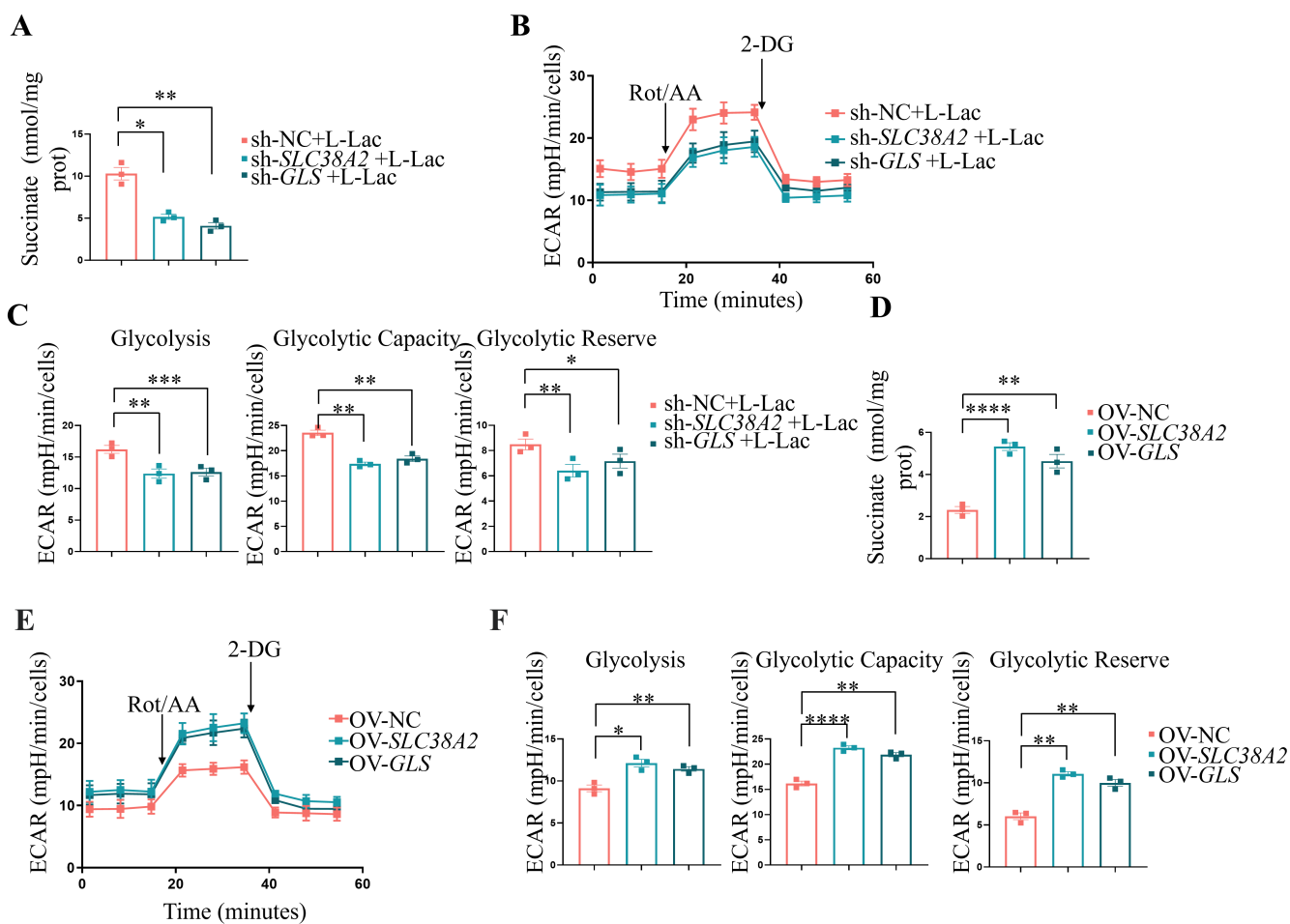

Supplement: Supplementary 1 — Figs. S1 to S20 Tables S1 and S2 [file research.1321.f1.zip › Figure S3(1).pdf]

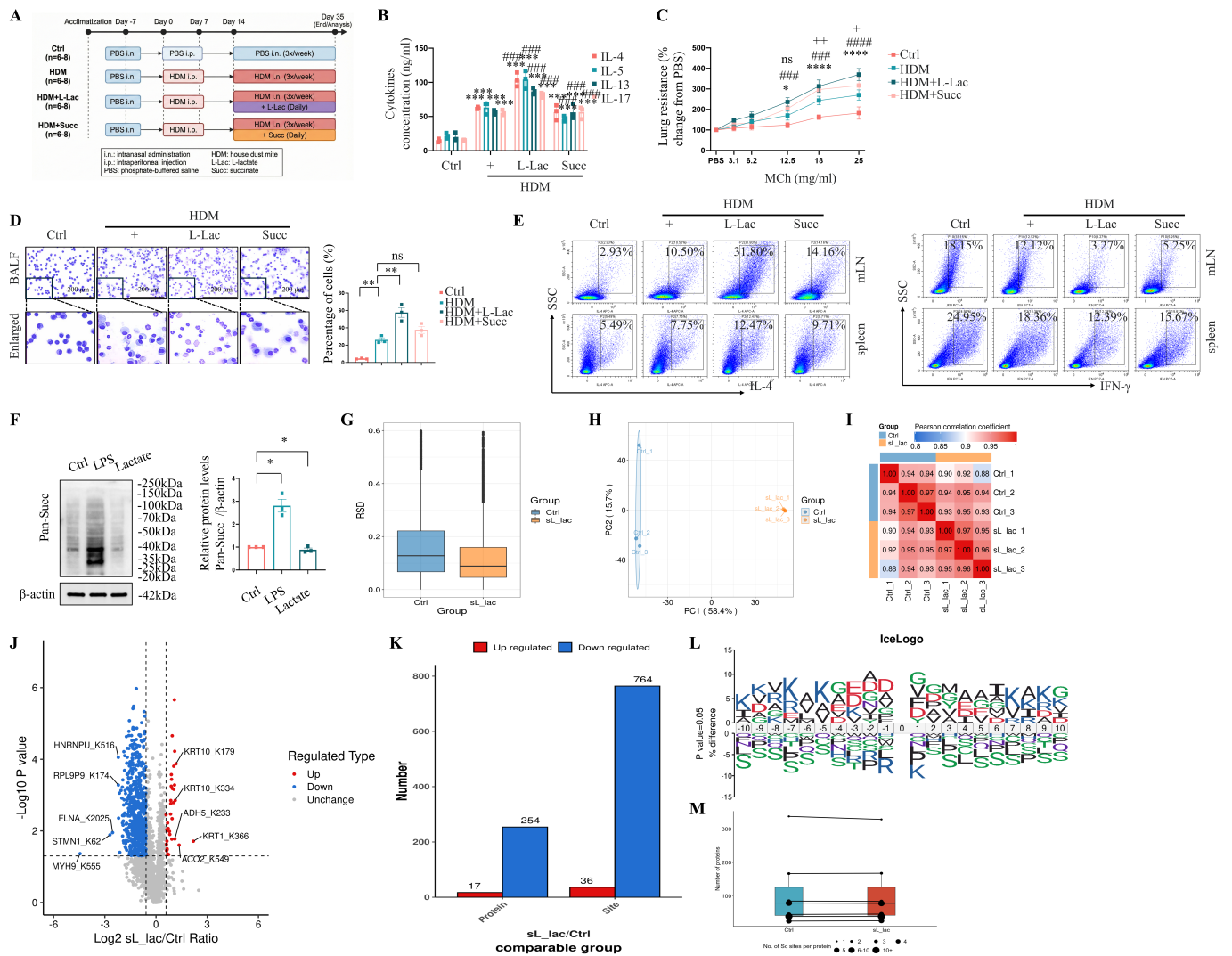

Supplement: Supplementary 1 — Figs. S1 to S20 Tables S1 and S2 [file research.1321.f1.zip › Figure S4(1).pdf]

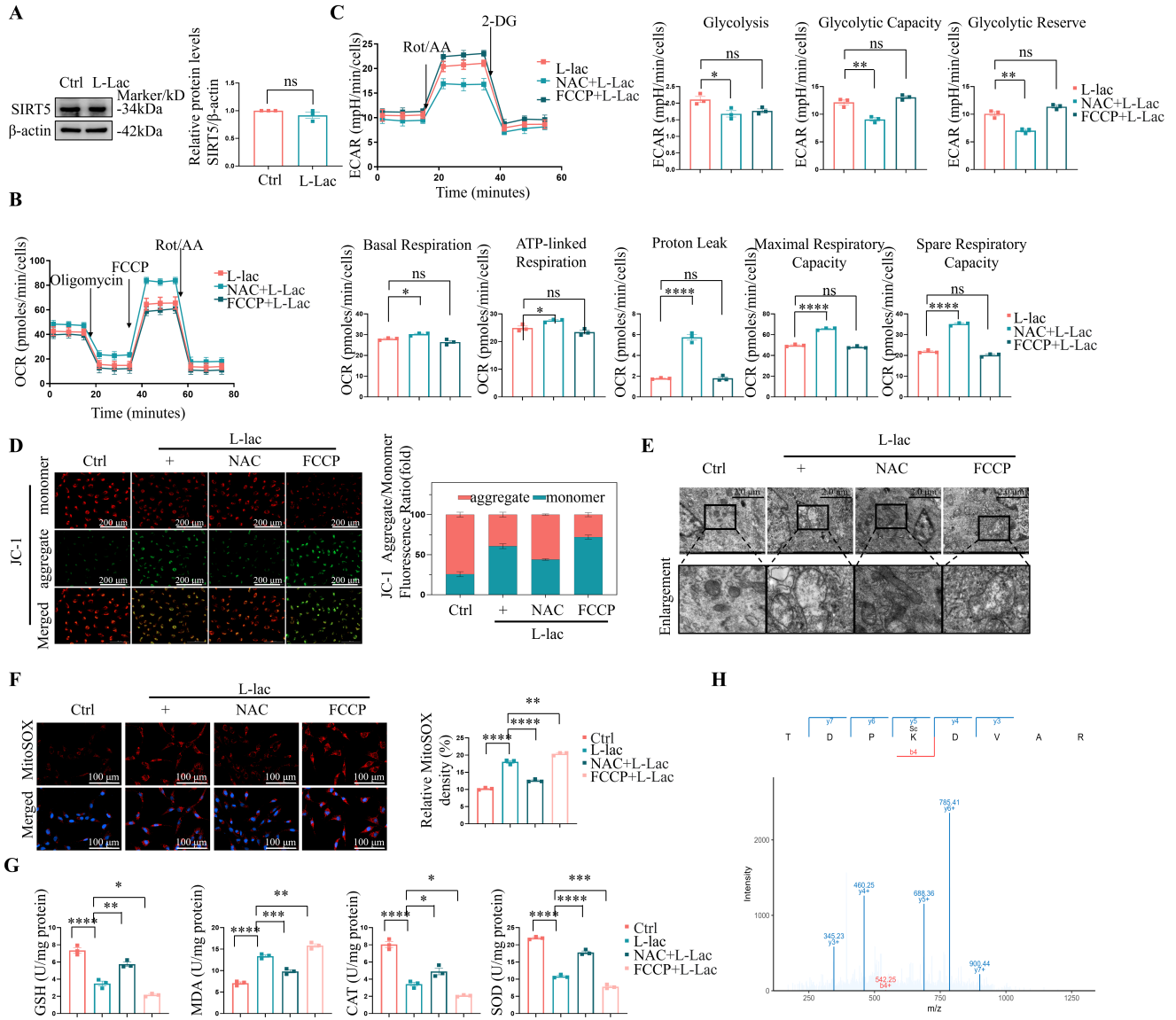

Supplement: Supplementary 1 — Figs. S1 to S20 Tables S1 and S2 [file research.1321.f1.zip › Figure S5(1).pdf]

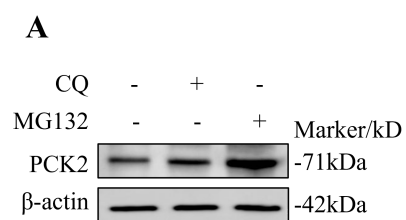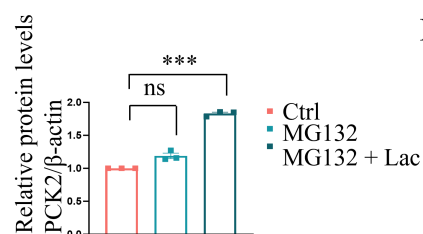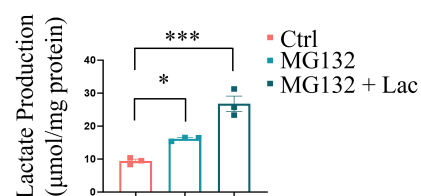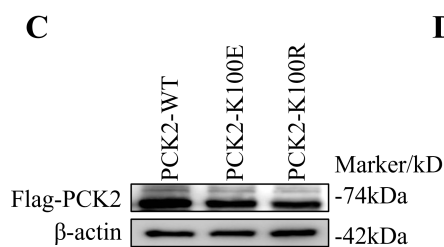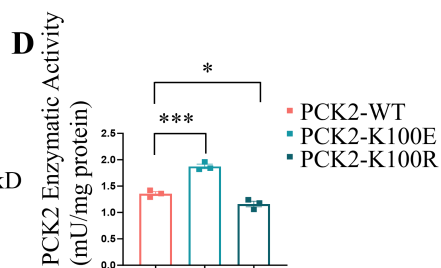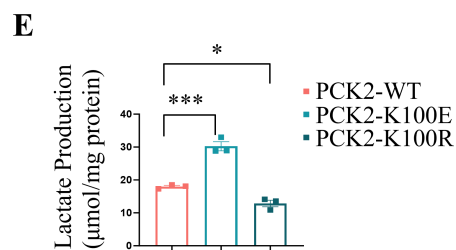

Supplement: Supplementary 1 — Figs. S1 to S20 Tables S1 and S2 [file research.1321.f1.zip › Figure S6.pdf]

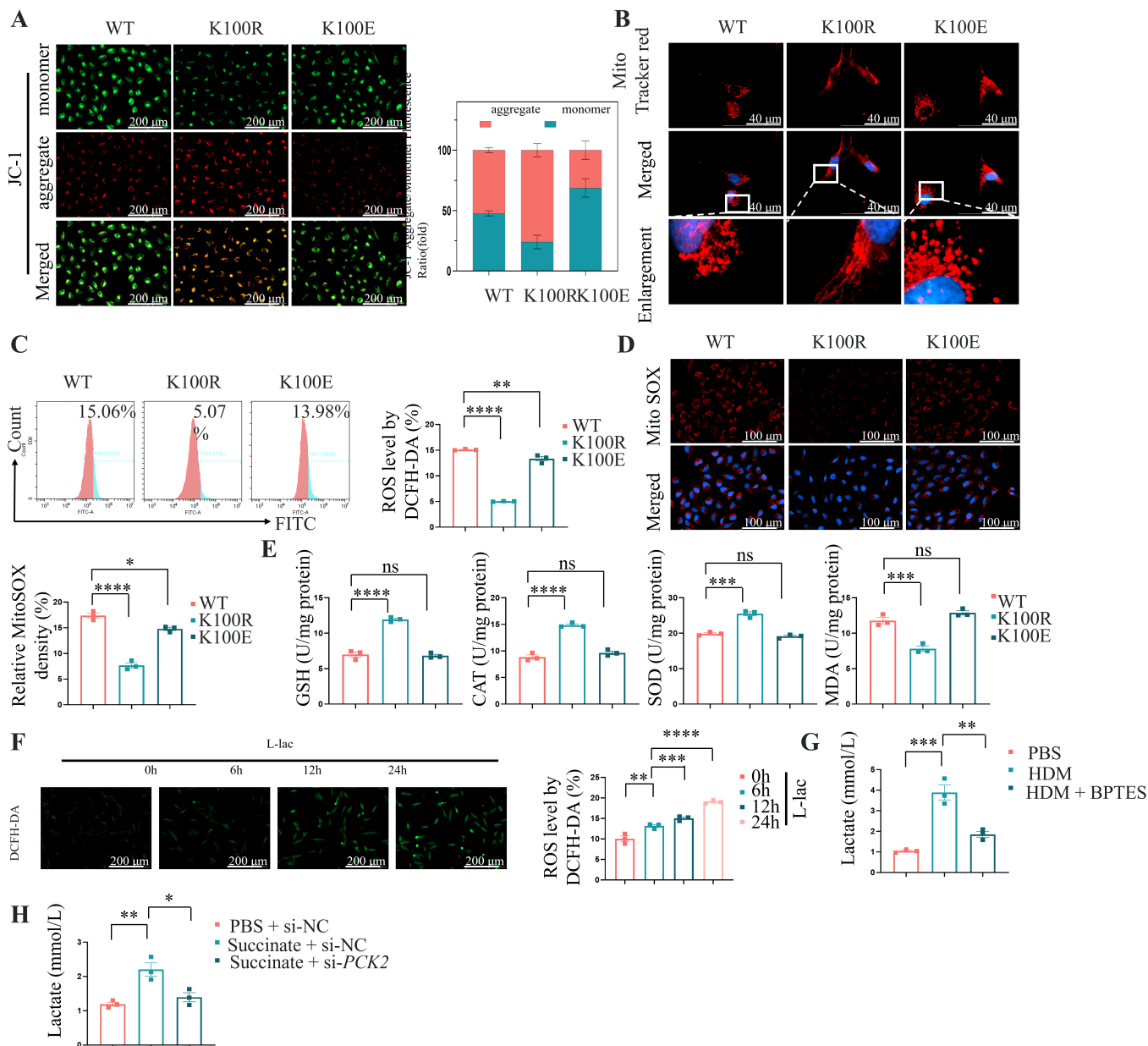

Supplement: Supplementary 1 — Figs. S1 to S20 Tables S1 and S2 [file research.1321.f1.zip › Figure S7.pdf]

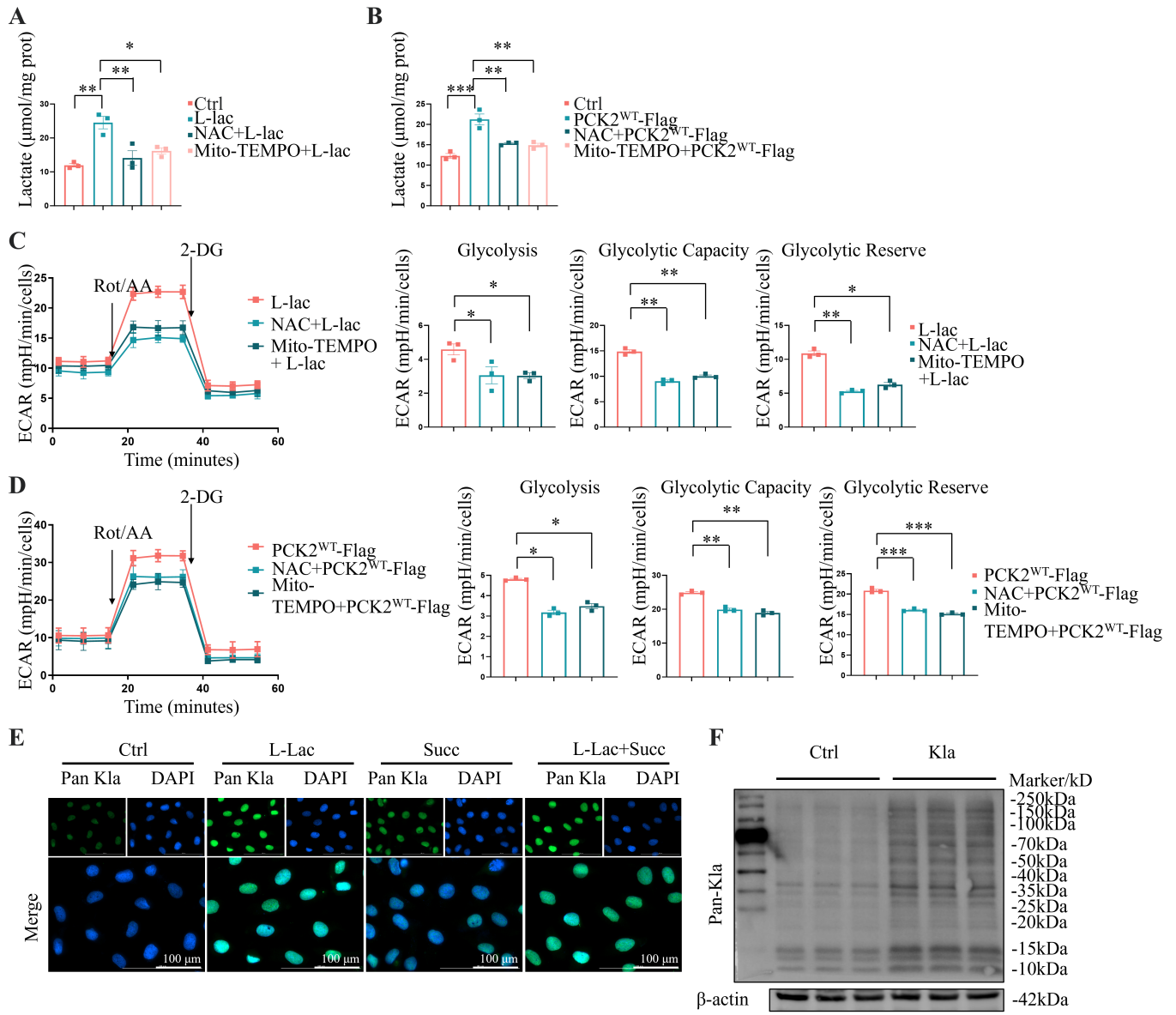

Supplement: Supplementary 1 — Figs. S1 to S20 Tables S1 and S2 [file research.1321.f1.zip › Figure S8.pdf]

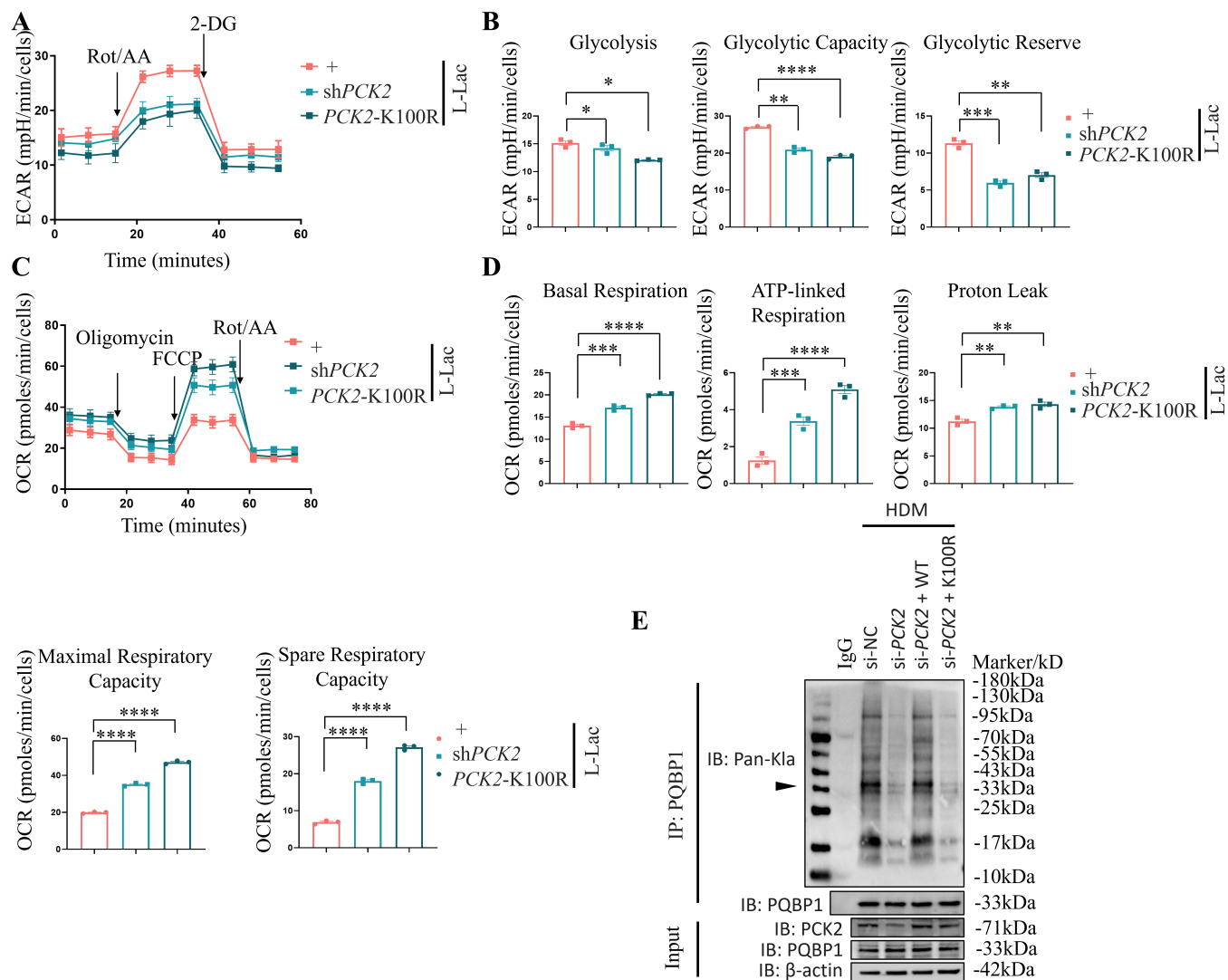

Supplement: Supplementary 1 — Figs. S1 to S20 Tables S1 and S2 [file research.1321.f1.zip › Figure S9.pdf]
